# Prostanoid Signaling in Cancers: Expression and Regulation Patterns of Enzymes and Receptors

**DOI:** 10.3390/biology11040590

**Published:** 2022-04-13

**Authors:** Pavel V. Ershov, Evgeniy O. Yablokov, Leonid A. Kaluzhskiy, Yuri V. Mezentsev, Alexis S. Ivanov

**Affiliations:** Institute of Biomedical Chemistry, 10 Building 8, Pogodinskaya Street, 119121 Moscow, Russia; evgeniy.yablokov@ibmc.msk.ru (E.O.Y.); leonid.kaluzhskiy@ibmc.msk.ru (L.A.K.); yuri.mezentsev@ibmc.msk.ru (Y.V.M.); alexei.ivanov@ibmc.msk.ru (A.S.I.)

**Keywords:** prostanoids, GPCR, tumors, cancers, gene expression, regulation, TCGA, CPTAC, overall survival, disease prognosis, predictive value

## Abstract

**Simple Summary:**

Neoplastic processes are accompanied by the reprogramming of cell metabolism and alteration of the balance between endogenous bioregulators with signaling functions. Prostanoid signaling is a part of the global arachidonic acid pathway and is associated with cancer progression. It includes prostanoids (prostacyclin, thromboxane, and prostaglandins E_2_, F_2α_, D_2_, H_2_), prostanoid metabolizing enzymes, and receptors. We focused on a comparative systematic analysis of expression patterns of target genes, encoding prostanoid enzymes and receptors. We also addressed the possible ways of their regulation at different levels in normal and tumor tissues (expression of genes and proteins, mutation and copy number landscape, promoter methylation status, prediction of tissue-specific master regulators, microRNAs). The results of the systematic analysis made it possible to suggest models of regulation of differentially expressed prostanoid enzymes and receptors. The associations between gene expression signatures and patients’ overall survival rates were established which can be valuable for translational biomedicine.

**Abstract:**

Cancer-associated disturbance of prostanoid signaling provides an aberrant accumulation of prostanoids. This signaling consists of 19 target genes, encoding metabolic enzymes and G-protein-coupled receptors, and prostanoids (prostacyclin, thromboxane, and prostaglandins E_2_, F_2α_, D_2_, H_2_). The study addresses the systems biology analysis of target genes in 24 solid tumors using a data mining pipeline. We analyzed differential expression patterns of genes and proteins, promoter methylation status as well as tissue-specific master regulators and microRNAs. Tumor types were clustered into several groups according to gene expression patterns. Target genes were characterized as low mutated in tumors, with the exception of melanoma. We found at least six ubiquitin ligases and eight protein kinases that post-translationally modified the most connected proteins PTGES3 and PTGIS. Models of regulation of *PTGIS* and *PTGIR* gene expression in lung and uterine cancers were suggested. For the first time, we found associations between the patient’s overall survival rates with nine multigene transcriptomics signatures in eight tumors. Expression patterns of each of the six target genes have predictive value with respect to cytostatic therapy response. One of the consequences of the study is an assumption of prostanoid-dependent (or independent) tumor phenotypes. Thus, pharmacologic targeting the prostanoid signaling could be a probable additional anticancer strategy.

## 1. Introduction

**Figure 1:**
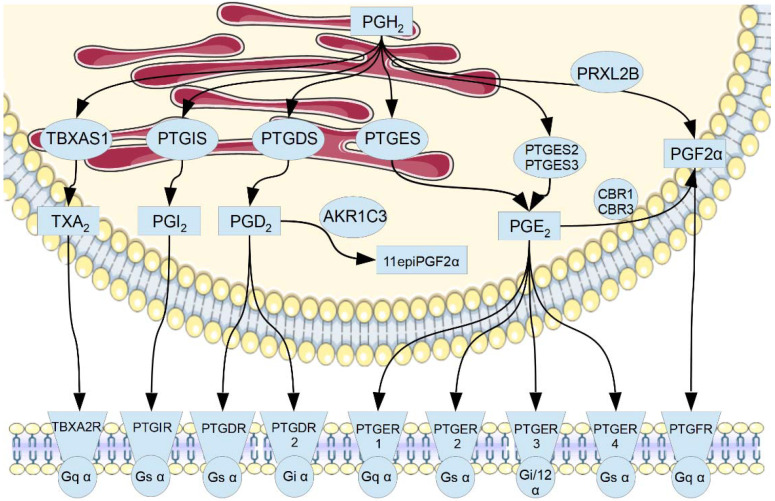
A scheme of prostanoid signaling: prostanoid-metabolizing enzymes (ellipses), G-protein-coupled prostanoid receptors (trapezes), type of G-subunit (circles), and prostanoids (rectangles). Proteins are shown according to their subcellular localization (endoplasmic reticulum, cytosol, and plasma membrane). Proteins’ and prostanoids’ names correspond to the list of abbreviations. Membrane and endoplasmic reticulum image templates were obtained from https://smart.servier.com/ (accessed on 3 February 2022).

Prostanoids (prostaglandins, prostacyclin, and thromboxane) are paracrine regulatory factors involved in signal transduction [1]. They have a systemic effect on many physiological processes under normal [2,3,4,5] and pathological conditions [6,7]. Determination of prostanoids in body fluids has diagnostic significance [8,9,10,11,12]. Prostanoid signaling is a functional cluster in the global arachidonic acid metabolism pathway [13] and consists of the enzymatic module (TBXAS1, PTGIS, PTGDS, PTGES1, PTGES2, PTGES3 and PRXL2B proteins) and the receptor module (TBXA2R, PTGIR, PTGDR, PTGFR, PTGDR2, PTGER1, PTGER2, PTGER3, PTGER4) (Figure 1).

Prostanoids are highly reactive factors and exported from “donor” cells via membrane transporters [14] and then, bind to surface receptors on “acceptor” cells [15,16,17]. The biomedical aspects of prostanoids in neoplastic transformation were thoroughly described in reviews [5,18,19] and confirmed in more recent experimental works [20,21,22,23,24,25,26,27,28,29,30,31,32,33,34,35,36,37] (Appendix A, Table A1). It can be noted that, firstly, correlations were established between the prostanoid levels in tumor tissues or in the blood (urinary) and the tumor growth, malignancy, and metastasis [22,23,24,26,28,29,30,32,33,34]. Secondly, prostanoids exert both tumor-suppressive and pro-neoplastic effects [25,37]. Thirdly, a correlation was found between the prostanoid content and tumor staging, histology, and patients’ survival rates [27,28,31]. Fourthly, inhibitors of prostanoid enzymes slowed down tumor growth and metastasis [21,35].

Data from integrative resources, for example, DisGeNET [38] and plenty of literature reports [39,40,41,42,43,44,45,46,47,48,49,50,51,52,53,54,55,56,57,58,59,60,61,62,63,64,65,66,67,68,69,70,71,72,73,74,75,76,77,78,79,80,81,82,83,84,85,86,87,88,89,90,91,92,93,94,95] (Table S1), clearly demonstrate the significance of genes, encoding prostanoid enzymes and receptors in cancers. It is to be noted that there are reports on correlations of gene expression and methylation patterns in different tumors with disease prognosis [45,46,47,48,52,58,64,65,66,69,70,74,75,79,80,81,85,86,89,90]. Tumors with over-expressed or under-expressed prostanoid enzymes and receptors [41,42,43,49,55,69,78,82,91] could determine the benefits of pharmacological correction of this metabolic cluster [44,77]. Indeed, exploration of tumor-specific gene expression and regulation patterns of prostanoid enzymes and receptors contributed to the discovery of new candidate biomarkers and drug targets [7,96,97,98], which are of great value for translational medicine. However, we did not find a bioinformatic pan-cancer and/or cancer-specific analysis of the expression and regulation patterns of the entire pool of prostanoid enzymes and receptors. Nowadays, despite the increased number of literature reports on the prostanoid signaling field, there is still a lack of a sophisticated understanding of the impact of transcriptomic, proteomic, and metabolomic factors on tumor promotion or suppression.

The aim of this work was to perform a comprehensive analysis of the gene expression and regulation patterns in prostanoid signaling in the most common human cancers. According to the expression patterns, prostanoid-dependent and independent tumors were conditionally selected. Models of tumor-specific regulation of gene expression including a repertoire of master regulators, microRNAs associated with cancers (oncomiRs), the methylation status of gene promoters, protein-protein interactions, and modifying proteins were predicted. The prognostic values of several transcriptomics signatures were also revealed.

## 2. Materials and Methods

### 2.1. Gene Expression Analysis

Differentially expressed genes (DEGs) were selected from the Cancer Genome Atlas (TCGA) [99] twenty-four tumor datasets. “TCGA tumor” and “TCGA normal and GTEx data” datasets were compared using web-based tool GEPIA2 [100] (accessed on 10 November 2021) at *p*-value < 0.01 and cut-off value log_2_FC = 1. Datasets of hematologic malignancies were not analyzed. Gene co-expression analysis was performed using GEPIA2. The UALCAN browser [101] (http://ualcan.path.uab.edu/, accessed on 13 November 2021) was used to retrieve the list of genes, which are co-expressed with target genes at Pearson correlation coefficient ≥ 0.75.

The prognostic value of gene expression patterns was explored using pan-cancer Kaplan-Meier plotter [102] (https://kmplot.com/analysis/, accessed on 9 December 2021) with the following settings: follow-up period—“60 months”; number of patients with available clinical data—“more than 100”, auto-select—“best cut-off”. Overall survival analysis was performed using the TCGA data. The predictive value of differentially expressed genes was explored using the ROC-plotter server (http://www.rocplot.org/, accessed on 5 December 2021) with breast, ovarian, glioblastoma, and colorectal tumor datasets [103,104,105] with outlier exclusion in plot settings.

### 2.2. Mutation Status Analysis

The somatic mutation frequency of genes in different tumors was analyzed using eight pan-cancer cBioPortal cohorts with a total of 47,942 clinical cases [106,107].

The web-based tool muTARGET [108] (accessed on 31 October 2021) was used to predict associations between the mutation status of target genes in tumors and the expression of other genes with the following options: mutation type—“all somatic mutations”; *p*-value cut-off “0.05”; fold change cut-off “2”; FDR cut-off “no FDR filter”.

### 2.3. Over-Representation Analysis

The WEB-based GEne SeT AnaLysis Toolkit (Webgestalt server) [109,110] was used to enrich gene sets with KEGG (Kyoto Encyclopedia of Genes and Genomes), Reactome, and Gene Ontology (GO) terms with the following settings: multiple test adjustment—“Benjamini-Yekutieli FDR-controlling method (FDR < 0.1)”, minimal number of gene for category—“5”, redundancy reduction—“affinity propagation”.

### 2.4. Master Regulators

The prediction of tissue-specific master regulators (MRs) for each DEG was performed in the hTFtarget database [111] (http://bioinfo.life.hust.edu.cn/hTFtarget, accessed on 3 December 2021). hTFtarget accumulates pairs of transcription factors/genes from human ChIP-Seq data (7190 experiment samples of 659 transcriptional factors) in 569 conditions (399 types of cell line, 129 classes of tissues or cells, and 141 kinds of treatments). Tumor-specific gene expression patterns and cancer hallmarks of MRs were explored using GEPIA2 and Cancer Gene Census portal [112], respectively.

### 2.5. Tumor-Specific Non-Coding microRNAs (oncomiRs)

The Condition-Specific miRNA Targets database CSmiRTar [113] (http://cosbi4.ee.ncku.edu.tw/CSmiRTar/, accessed on 17 November 2021) was used to predict microRNA that is associated with cancer (oncomiRs) that can potentially participate in post-transcriptional regulation of target genes in prostanoid signaling. This server is linked to the five databases: DIANA-microT, miRanda.org, miRDB, Targetscan, and miRTarBase. The selection of predicted oncomiRs was performed by following settings: species—“human”, class of disease—“cancer”, average normalized score > 0.4 (for data availability at least in 3 of 5 supported databases). The OncoMir cancer database (OMCD) (www.oncomir.umn.edu/omcd, accessed on 21 November 2021) [114] was further used to study differential expression of oncomiRs with *p*-value threshold < 0.05 and fold change cut-off values > 2 or <0.5.

### 2.6. Promoter Methylation Status

The promoter methylation status of target genes was analyzed using the UALCAN browser. The beta-value indicates the level of DNA methylation ranging from 0 (unmethylated) to 1 (fully methylated). Different beta value cut-offs have been considered to indicate hypermethylation (beta value: 0.7–0.5) or hypomethylation (beta-value: 0.3–0.25) [115,116].

### 2.7. Protein Expression Analysis

Tumor-specific protein expression was explored using The Human Protein Atlas (HPA) portal version 21.0 [117] (https://www.proteinatlas.org/, accessed on 26 November 2021). The UALCAN browser was used to search for differentially expressed proteins (DEPs) within CPTAC datasets (breast, ovarian, colon, lung tumors, and uterine corpus endometrial carcinoma) [118]. Z-values represent standard deviations from the median across samples for the given cancer type. Log_2_ spectral count ratio values from CPTAC were first normalized within each sample profile, then normalized across samples.

### 2.8. Post-Translational Modifications

Modifying proteins carrying out post-translational modifications (PTMs) of prostanoid enzymes and receptors were retrieved from the Biogrid portal [119] (accessed on 10 January 2022). Only physical PPIs and matched subcellular localization profiles (according to the COMPARTMENTS database [120]) were used for the final selection of modifying proteins.

### 2.9. Protein-Protein Interaction Network Analysis

A list of known protein partners of PTGIS и PTGES3 was retrieved from STRINGdb v. 11.5 [121] (https://string-db.org/, accessed on 8 January 2022) with the following settings: organism—Homo sapiens; interaction sources—experiments; cut-off interaction score (combined score) = 0.4. The compartment database [120] (https://compartments.jensenlab.org/Search, accessed on 10 January 2022) was used for the subcellular localization analysis of proteins. Cytoscape software v. 3.8.2 was used for PPI network visualization [122].

### 2.10. Cluster Analysis of Gene Expression Matrix

Principal Component Analysis (PCA) and Classification and Regression Trees (CRT) methods were used to cluster DEGs in different tumors selected by |log_2_FC| tumor/normal > 1 and *p* < 0.01. For the PCA method, the gene expression matrix was prepared by assigning integer values (+2) or (−2) to the up- or down-regulated genes, respectively. Genes with |log_2_FC| < 1 were assigned zero value. The ClustVis web-based tool was used for PCA analysis and result visualization [123]. Data preprocessing options were the following: data transformation—“no transformation”; row scaling—“unit variance scaling”; PCA methods—“SVD with imputation”. Heat map options: clustering distance for rows—“Manhattan”; clustering methods for rows—“average”.

The CRT clusterization was performed by IBM SPSS Statistics v. 23 software with the Decision Tree algorithm. CRT classifies cases into groups of dependent variables based on the values of independent variables. CRT splits the data into segments that are as homogeneous as possible with respect to the dependent variable. A terminal node in which all cases have the same value for the dependent variable is a homogenous, “pure” node. The variables used were: Expression, dependent, and Gene, independent. Expression values were: NEGATIVE, POSITIVE, and NODIFF. The definitions of Expression values were as below. NEGATIVE: the gene expression level in the tumor datasets was decreased compared to the normal tissue datasets. POSITIVE: the gene expression level in the tumor datasets was increased compared to the normal tissue datasets. NODIFF: no changes were shown for the gene expression level in tumor and normal tissue datasets. Gene values were gene names. The Decision Tree procedure was used with the settings below: validation method—“cross-validation”, minimum cases in parent node—“100”, minimum cases in child node—“50”, level growth limits—“5”, impurity measure—“twoing”.

### 2.11. Other

The alignment of gene lists was performed using Venn diagrams (https://bioinformatics.psb.ugent.be/cgi-bin/liste/Venn/, accessed on 10 January 2022).

### 2.12. Data Mining

**Figure 2:**
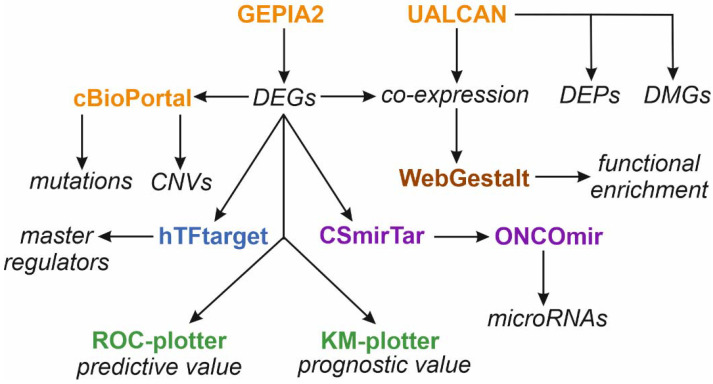
A flowchart of data mining using web-based bioinformatic tools: GEPIA2, UALCAN, and cBioPortal were used for the analysis of The Cancer Genome Atlas (TCGA) and Clinical Proteomic Tumor Analysis Consortium data (CPTAC); WebGestalt—WEB-based GEne SeT AnaLysis Toolkit; hTFtarget—database for regulations of human transcription factors and their targets; CSmirTar—Condition-Specific miRNA Targets database; ONCOmir—OncoMir Cancer Database. ROC-plotter—ROC-plotter server; KM-plotter—Kaplan-Meier plotter server. Abbreviations: DEGs—differentially expressed genes; DEPs—differentially expressed proteins; DMGs—differentially methylated genes; CNVs—copy number variations; mutations—cancer-specific mutation frequency of target genes.

Molecular profiling data on 19 target genes, encoding prostanoid enzymes and receptors, from 24 solid tumors were analyzed using a data mining pipeline (Figure 2). The Cancer Genome Atlas (TCGA) portal was the main data-source for the gene expression and co-expression patterns as well as promoter methylation status. Tumor-specific protein expression data were retrieved from Clinical Proteomic Tumor Analysis Consortium (CPTAC) portal. Several web-based tools (GEPIA2, UALCAN, cBioPortal) were adapted for comparative statistical analysis of tumor and normal TCGA or CPTAC datasets to identify DEGs and DEPs. hTFtarget and CSmirTar portals were used for the prediction of master regulators and microRNAs for a subset of target genes, respectively. Finally, predictive and prognostic values of gene expression patterns were investigated using ROC-plotter and KM-plotter, respectively. Over-representation analysis was performed in the WebGestalt server.

## 3. Results

### 3.1. Gene Expression Analysis

**Figure 3:**
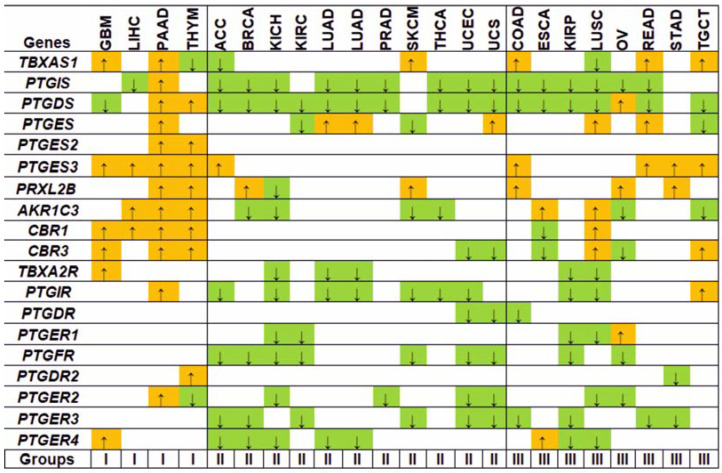
A landscape of differentially expressed genes, encoding prostanoid-metabolizing enzymes (*TBXAS1*, *PTGIS*, *PTGDS*, *PTGES*, *PTGES2*, *PTGES3*, *PRXL2B*, *AKR1C3*, *CBR1*, *CBR3*) and receptors (*TBXA2R*, *PTGIR*, *PTGDR*, *PTGFR*, *PTGDR2*, *PTGER1*, *PTGER2*, *PTGER3*, *PTGER4*), in different tumors. Statistically significant changes (fold change cutoff = 2) in tumor/normal tissues are shown by arrows. “Groups” correspond to groups of tumors distinguished according to a similar pattern of DEGs. Up- and down-regulated genes are highlighted with orange and green colors, respectively. Genes’ and tumors’ names correspond to the list of abbreviations.

The gene expression landscape of the prostanoid signaling in different tumors is shown in Figure 3. At least four groups of tumors can be conditionally distinguished according to a similar pattern of DEGs. In Figure 3, it was shown that group I consists of predominantly up-regulated DEGs in GBM, LIHC, PAAD, and THYM tumors. Group II has predominantly down-regulated DEGs in ACC, BRCA, KICH, KIRC, LUAD, PRAD, SKCM, THCA, UCES, and UCS tumors. Group III consists of both up- and down-regulated genes in COAD, ESCA, KIRP, LUSC, OV, READ, STAD, and TGCT. Group IV with HNSC and LGG tumors (not shown in Figure 3) does not contain DEGs.

Groups I and II with contrasted gene expression patterns are the most interesting for analysis. Up-regulation of genes, encoding enzymes, which produce pro-neoplastic prostanoids, can lead to tumor promotion and prostanoid-dependent tumor phenotype. Down-regulation of such genes may indicate tissue-specific responses to neoplastic transformation or cancer-driven metabolism reprogramming aimed to reduce the accumulation of tumor-suppressive prostanoids. To test these assumptions indirectly, correlations between the DEGs and disease prognosis were explored. We also considered the theory that the overexpressed genes (Figure 3) could be critical for tumor cell viability. In other words, how sensitive tumor cells are to knockouts and knockdowns of genes in prostanoid signaling. We analyzed the data of pan-cancer Clustered Regularly Interspaced Short Palindromic Repeats (CRISPR) and RNA interference (RNAi) screens on the DepMap portal [124]. A general rule is that the lower the gene effect score, the more its dependency in a cell line, so a score close to −1 corresponds to genes that can be essential for a cell line viability [124]. Table S2 shows the gene effect scores. Knockouts/knockdowns of several target genes with scores below −0.5 (an accepted cutoff value) can be related to tumor cell viability (Table S2): *TBXAS1* (gallbladder adenocarcinoma, OCUG1 cell culture), *PTGDR* (lung cancer, CORL279), *PTGER4* (lymphoma, C8166) and *PTGES3* (brain cancer, ONS76). Incidentally, *PTGES3*, being the most common DEG in various tumors (Figure 3), tends to be an essential gene in tumor cell lines (Table S2). At the same time, there is an absence of a significant effect in cell lines caused by the “separate switching off” of most of the target genes. Thus, it allows us to consider these genes as non-essential for tumor cell viability.

**Figure 4:**
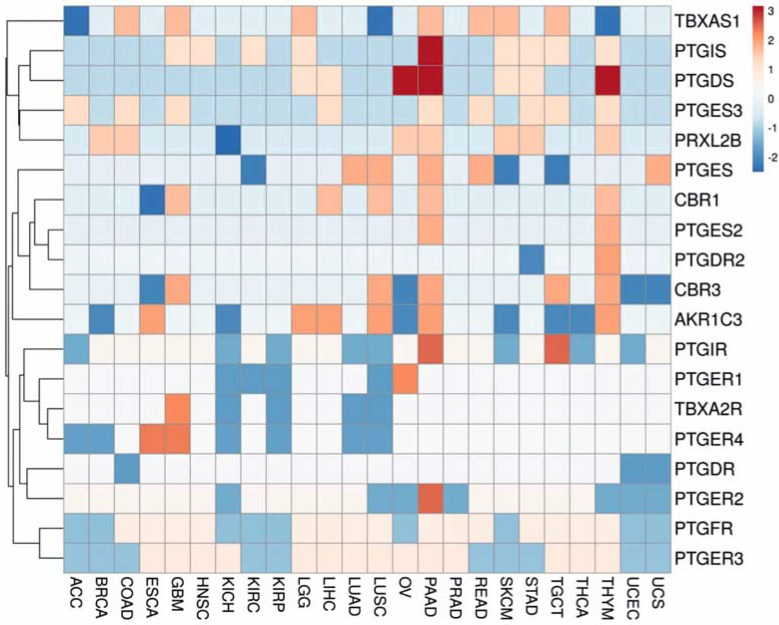
Principal component analysis of differentially expressed genes, encoding prostanoid metabolizing enzymes and prostanoid receptors, in different tumors; color scale shows cluster distances.

**Figure A1:**
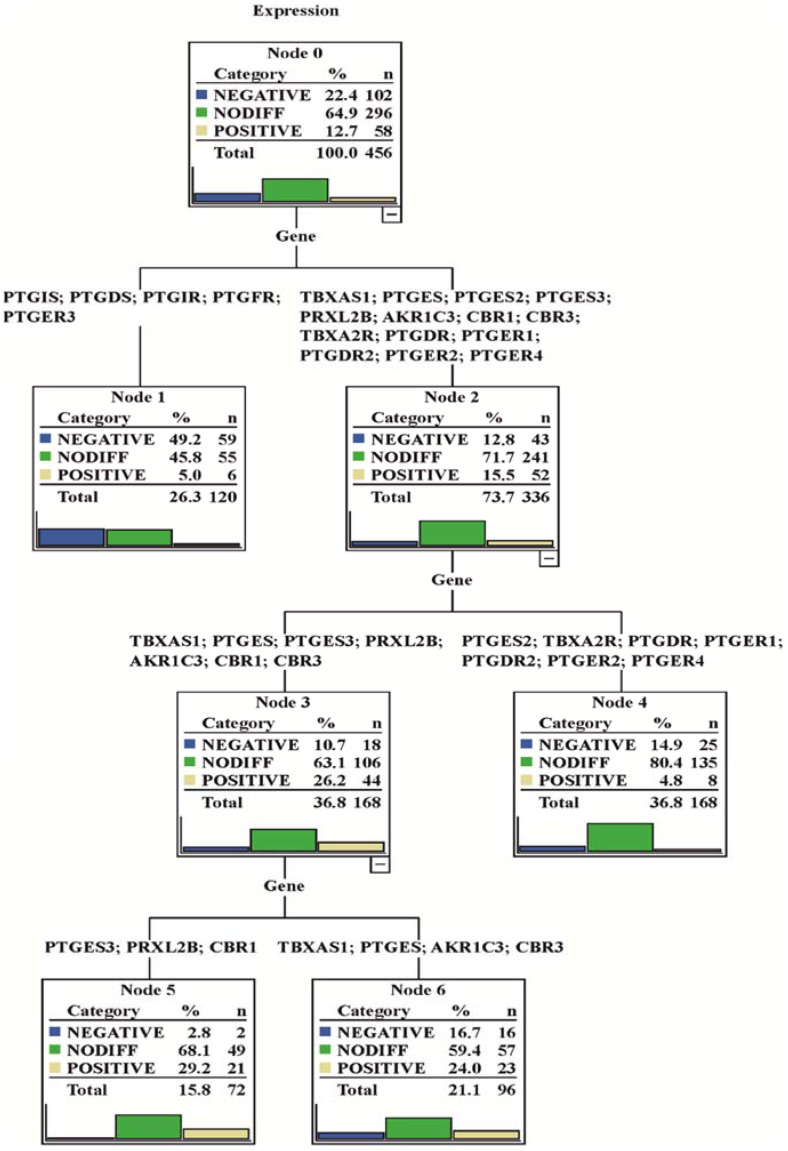
Classification and Regression Trees cluster analysis of differentially expressed genes, encoding prostanoid metabolizing enzymes and prostanoid receptors in tumors.

Cluster analysis was used to find outmatched expression patterns of target DEGs within a set of tumors. Comparing the results obtained by two clustering methods, the Classification and Regression Trees (CRT) (Appendix B, Figure A1) and the Principal Component Analysis (PCA) (Figure 4), subclusters with matched gene expression patterns (*PTGIS-PTGDS*, *PTGES2-PTGDR2*, *TBXA2R-PTGER4-PTGER1-PTGER-PTGDR*, and *PTGES3-PRXL2B*) were identified.

PCA method indicated three large clusters. The first and second expression clusters consist of genes encoding prostanoid enzymes: *TBXAS1*, *PTGIS*, *PTGDS*, *PTGES3*, *PRXL2B*, and *PTGES*, *PTGES2*, *AKR1C3*, *CBR1*, *CBR3*, respectively. The third cluster is represented exclusively by genes encoding prostanoid receptors. As for PCA clusters, high Pearson correlation coefficients of gene expression within the cluster were only in case of *AKR1C3*, *CBR1*, *CBR3* genes (r = 0.64 − 0.77, *p*-value < 0.05) in LUSC tumor as well as CBR1 and CBR3 (r = 0.72 − 0.86, *p*-value < 0.05) in ESCA tumor. There was no significant correlation in respective normal tissues. In the third PCA cluster, co-expression between the *PTGIR* and *TBXAR2* genes (r = 0.67 − 0.82, *p*-value < 0.01) was found in ESCA, KIRP, LICH and READ tumors. Other pairs of co-expressed genes encoding prostanoid receptors were as follows: *PTGIR-PTGER2*, r = 0.72 − 0.75, TGCT tumor; *PTGER4-PTGER2*, 0.68–0.78, SKCM tumor; *PTGDR-PTGER2*, r = 0.66 − 0.74, SKCM tumor; *PTGDR-PTGER3*, 0.59–0.67, PAAD tumor.

Next, we searched for other genes whose expression patterns correlate with the target genes in each of the three PCA clusters (Figure 4). It should be said that the total number of found co-expressed genes for the first cluster was about 10 times higher than for the second cluster. Gene sets co-expressed with *TBXAS1* and *PTGDS* in a subset of tumors (KIRC, LIHC, GBM, PCPG, OV, ACC, LGG, UVM, ESCA, CHOL) are enriched in immune reaction and phagocytosis pathway terms (Table S3). Gene sets co-expressed with *PTGIS* in DLCA, CHOL, COAD, READ, PRAD, TGCT tumors, and *PTGES* in ACC tumors are enriched in “muscle contraction” and “extracellular matrix organization” terms. Gene sets co-expressed with *PTGES3* in THCA and SKCM tumors are enriched with the “mRNA processing” term (Table S3). Gene sets co-expressed with genes, encoding prostanoids receptors TBXA2R, PTGFR, and PTGER3, are involved in immune responses and structural processes (elastic fibers formation, collagen polymerization/depolymerization) as well as the organization of the extracellular matrix (Table S3) in COAD and TGCT tumors. These findings indicate the leading cellular processes that accompany prostanoid signaling in different tumors. These terms are in good agreement with the known effects of prostanoids on blood vessel smooth muscle proliferation in the regulation of cardiovascular homeostasis [125,126], actin cytoskeleton reorganization via stimulation of stress fiber formation [127], and the immune response modulation [127,128,129]. Enrichment analysis also shows the association of prostanoid enzymes and receptors with focal adhesion kinase 1 (PTK2), which participates in the neoplastic transformation via activation of Wnt/β-catenin signaling [130].

### 3.2. Regulation Patterns of Differentially Expressed Genes

#### 3.2.1. Promoter Methylation

Differences in the transcript accumulation in normal and tumor tissues may be the result of several competing factors at the transcriptional and post-transcriptional levels such as the methylation status of gene promoters, combination of transcriptional master regulators, and transcript stability. Analysis of promoter methylation status of the target genes show *PTGDR*, *PTGER3*, *PTGIR*, and *TBXA2R* down-regulation in a number of tumors which may be partly due to the elevation of promoter methylation (Table S4). On the other hand, for up-regulated *CBR3* and *AKR1C3* genes in LUSC tumors, there is an agreement with promoter hypomethylation. The down-regulation of *PTGDS* in ESCA and HNSC tumors was accompanied by a statistically significant decrease in promoter methylation while remaining in the hypermethylation range.

#### 3.2.2. Master Regulators

Transcriptional master regulators (MRs) mean DNA-binding and chromatin remodeling proteins [131], which are capable of regulating the expression of genes-of-interest. We selected only tissue-specific MRs that were predicted for ≥80% of all the target DEGs. Table 1 shows the distribution of 21 MRs that could potentially be responsible for the differential expression of target genes in tumors. Master regulators such as BRD4, CTCF, EP300, FOXA1, and SPI1 are expressed in breast, brain, colorectal, kidney, pancreatic, prostate, skin, and stomach tumors (Table 1) enriched with cancer hallmarks and encoded by cancer driver genes. It is noteworthy that most of the found MRs are overexpressed in tumors. This speaks in favor of the involvement of the predicted MRs in the neoplastic transformation that is also stressed by the participation of AR, EP300, MAX, RELA, SP1, and SPI1 proteins in cancer-related pathways (Table S5).

#### 3.2.3. OncomiRs

**Figure 5:**
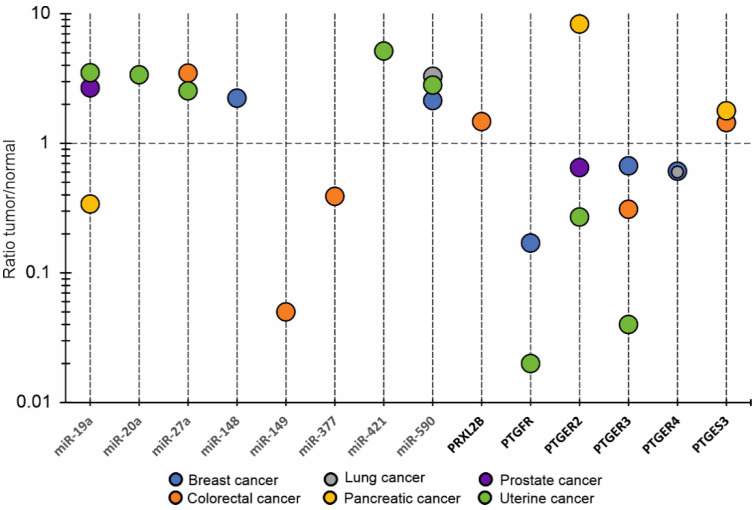
Comparative analysis of expression patterns of oncomiRs and genes, encoding prostanoid-metabolizing enzymes and prostanoid receptors, in different tumors. Genes’ names correspond to the list of abbreviations.

A list of regulatory oncomiRs was also predicted for all of the target DEGs (Table S6) and oncomiRs expression patterns in tumors were explored. The number of oncomiRs for prostanoid receptors was much higher than for prostanoid enzymes, decreasing in the following line: *PTGER4-PTGER3-PTGER2-PTGDR-TBXAR2-PTGFR*. Table S7 shows the tumor/normal ratios of “master oncomiRs”, which can potentially regulate at least two target transcripts. It turned out unexpectedly that some oncomiRs were predicted as universal post-transcriptional regulators for both prostanoid enzymes and receptors such as miR-149-3p (*PRXL2B*, *PTGES*, *TBXA2R*), miR-19a-3p (*PTGES3*, *PTGER2*), miR-338-5p (*PTGES3*, *PTGDR*), miR-421 (*PTGES3*, *PTGER2*); miR-423-5p (*PRXL2B*, *PTGFR*), miR-508-5p (*PTGES*, *TBXA2R*). Most oncomiRs are up-regulated in tumors as compared to corresponding normal tissues. Findings on changes in tumor-specific expression of oncomiRs and target transcripts are shown in Figure 5.

Thus, it can be assumed that the accumulation of oncomiRs may be in inverse proportion with that of target transcripts. This can be traced in contrasting pairs such as miR-19a/*PTGER2* (pancreatic tumor); miR-421/*PTGER2* (uterine tumor); miR-590/*PTGFR* (uterine and breast tumor); miR-20a/*PTGER3* (uterine tumor) (Figure 5).

### 3.3. Protein Expression Patterns

According to The Human Proteome Atlas (HPA), positive immunohistochemical staining (IHC) is shown for the majority of prostanoid enzymes in tumors (Table S8). IHC protein expression data on prostanoid receptors, with the exception of PTGER4, are not yet available in HPA (Table S8). This group can be characterized as low expressed in normal and tumor tissues. *PTGDR*, *PTGDR2*, *PTGER1*, *PTGER3*, and *PTGFR* genes have the median expression value of about 1 FPKM (Fragments Per Kilobase Million), while other genes are expressed in the range of 1–5 FPKM, regardless of cancer specificity. A concordance between the transcripts and protein accumulation in normal and tumor tissues was performed by comparing the TCGA and CPTAC data in BRCA, COAD, LUAD, OV, and UCES tumors (Table 2). From Table 2, it follows that there is a concordance between the transcripts and total protein accumulation. However, up-regulation of *PRXL2B* is not accompanied by an increase in the protein content in BRCA and OV tumors. There is an inverse relationship between the up-regulated *PRXL2B* and *PTGDS* genes and the protein accumulation in COAD and OV tumors, respectively. The general observation is that a decrease in mRNA accumulation leads to a decrease in total protein, which is quite logical. It is assumed that when transcript level increases, but protein content remains unchanged or reduced, there is a translational and/or post-translational regulation.

### 3.4. Prognostic Value of Transcriptomic Signatures

We explored the target genes, encoding the prostanoid enzymes and receptors, in terms of their disease prognostic value. Survival analysis was performed, and all relevant results obtained are presented in Table 3. In particular, Figure S1 shows overall survival curves in groups with high and low gene expression (without subgroup analysis) followed by an assessment of tumor-specific signatures (Table 3). A “pure” tumor specificity of signatures is achieved in the subgroups stratified by gender, stage, grade, and mutation burden. This, however, is balanced by a reduction of clinical cases in a subgroup and the power of a statistical test. Most of the transcriptomic signatures (without subgroup analysis) still show acceptable tumor specificity with the exception of the signature containing PTGDS, CBR3, PTGIR, PTGFR, PTGDR2, and PTGER3 genes in HNSC tumor, which is similar to other tumors (BRCA, CESC, LUAD, SARC, and UCES).

### 3.5. Predictive Value of Prostanoid Enzymes and Receptors Genes

We explored the associations between gene expression of each target gene and responses to anticancer drug treatment of breast, ovarian, glioblastoma, and colorectal tumors using the Receiver Operating Characteristic plotter (ROC-plotter) [103]. It was found that only for breast or ovarian tumors, ROC curves for (*PTGIS*, *PTGES*, and *PTGER4*) or (*TBXAS1*, *PTGES*, *TBXA2R*, and *PTGDR2*), respectively, had Area Under Curve (AUC) values > 0.75 at the tumor/normal fold changes ≥ 2 (Figure S2A,B). *PTGIS*, *PTGES*, and *PTGER4* up-regulation in the group of responders with HER2-positive breast cancer, known by its aggressive behavior [132,133], is associated with complete tumor response to treatment with fluorouracil, epirubicin, and cyclophosphamide (FEC). On the other hand, the same amplitude of down-regulation of *PTGES* and *PTGDR2* gene expression in the group with serous ovarian cancer grade G3 correlates with relapse-free survival at 6 months and the response to taxanes and platinum treatment, respectively (Figure S2B). Thus, we observed the associations between the gene expression patterns and responses to cytostatic therapy, while there were no associations in the case of targeted therapy (Trastuzumab, Tamoxifen, Avastin, and aromatase inhibitors).

### 3.6. Mutation Status Analysis

It was found that the highest frequency of somatic mutations in target genes was in melanoma (cut-off = 0.5%, *n* > 100). Next, cBioPortal cohorts “Skin Cutaneous Melanoma TCGA PanCancer Atlas” (*n* = 448), “TCGA Firehose Legacy” (*n* = 479) and “DFCI Nature Medicine 2019 metastatic melanoma” (*n* = 144) were analyzed. Mutation frequency rates at 5–6%, 5–7%, 2.8–5%, 3–4%, 7–9%, 2.1–5% were observed in *TBXAS1*, *PTGIS*, *AKR1C3*, *PTGDR*, *PTGFR* and *PTGER3* genes, respectively. muTARGET web-based tool allowed us to predict genes, which expression patterns can be associated with genetic polymorphism in group #1 (*TBXAS1*, *PTGIS*, and *AKR1C3*) or group #2 (*PTGDR*, *PTGFR*, and *PTGER3*) in melanoma. Thirty six down- and three up-regulated genes were predicted for group #1, and 14 and 6 for group #2, respectively (Table S9). Down-regulated genes were enriched with the following GO terms: “extracellular region”, “extracellular exosome”, “heparin binding” (*BMP7*, *FMOD*, *PCSK6*, and *THBS4*), “cell adhesion” (*ATP1B2*, *BCAN*, *CNTN4*, *PKP1*, *RELN*, *ROBO2*, *SPON1*, *SVEP1*, and *THBS4*), “epidermal growth factor-like domain” (*BCAN*, *PCSK6*, *RELN*, *SCUBE3*, *SVEP1*, and *THBS4*), “fibronectin type III domain” (*CNTN4*, *ROBO2*, *SDK2*, and *SORL1*). A subset of up-regulated genes was represented by *ILDR2*, *CDH2*, *RNF128*, *GBP1*, *PDCD1LG2*, *ALDH1A2*, *ADAMTS14*, *RN7SK*, and *RGS5*, however, statistically significant GO terms were not found.

## 4. Discussion

Pan-cancer analysis showed several interesting gene expression patterns in prostanoid signaling that were used to model tissue-specific regulation patterns (Table S10). Transcriptomic data were retrieved from the TCGA portal, which actually contains data on most tumor types and subtypes. At the same time, the availability of proteomic information in the CPTAC portal is more limited. For this reason, we compared the gene expression patterns of target genes, their predicted master regulators, and oncomiRs in several tumors. It could be suggested that the up-regulation of *AKR1C3*, *CBR1*, and *CBR3* genes from PCA cluster 2 (Figure 4) in LUSC tumors may occur due to promoter hypomethylation and gene expression changes of master regulators *FOXA2*, *LMNB1*, *SPI1*, miR-511 (Table S10). In contrast, down-regulation of co-expressed genes *CBR1* and *CBR3* in ESCA tumors can be associated with elevated accumulation of miR-335 (for *CBR1*) and a decrease in promoter methylation status similar to that in LUSC. The up-regulation of all ten genes, encoding prostanoid enzymes, in PAAD tumors compared to normal tissue is noteworthy. This expression pattern is not found in any other tumors, except for THYM tumors, where only seven of ten genes are up-regulated. It follows from Table S10 that at least half of DEGs are characterized by an increase in copy number variations (amplification type) indexed in the cBioPortal UTSW Nat. Commun. 2015 cohort. We have found no statistically significant changes in the promoter methylation status in PAAD tumors compared to normal tissue. However, up-regulated *PTGDS*, *AKR1C3*, and *CBR3* genes were hypermethylated, an epigenetic situation that also occurs in tumors [134]. The accumulation of *PTGES3* and *PRXL2B* transcripts in tumor tissue correlates with a simultaneous reduction of oncomiRs miR-223, miR-19a, miR-605, and miR-486, miR-211, miR-423, respectively. It should be noted that some of those are tumor suppressors [135,136,137]. Up-regulation of ten DEGs in PAAD tumor is in concordance with the up-regulation of master regulators *CTCF*, *IRF1*, and *KLF4*, while *POLR2A* and *STAG1* remain unchanged. It is well known that transcriptional activation of master regulators is critical for tumor progression, in particular, for pancreatic [138] and colorectal cancers [139,140].

Prostacyclin, a metabolite produced by prostacyclin synthase (PTGIS), is historically believed to exert tumor-suppressive effects [141,142] and lowers its level along with down-regulation of PTGIS associated with an aggressive tumor phenotype and a poor disease prognosis [43]. Figure 3 shows a simultaneous decrease in the expression of both *PTGIS* and *PTGIR* in eight tumor types, and therefore, we analyzed the possible causes of such changes (Table S10). It can be pointed out that down-regulation of *PTGIS* in KIRP, LUAD, THCA, and UCES tumors is accompanied by an increase in miR-34a levels, which mainly plays a considerable role in inhibiting tumor progression in thyroid tumors [143]. In LUSC tumors, there is a decrease in miR-34c, which, like miR-34a, possesses antitumor activity [144]. On the other hand, miR-326 expression is suppressed in lung cancer tissues. This oncomiR, as shown in [145], inhibits lung cancer cell proliferation, and colony formation and provokes apoptosis. It should also mention that the down-regulation of *PTGIS* is comparable to the lowering of a corresponding protein product in LUAD and UCEC tumors (Table 2). As for the predicted transcriptional master regulators of PTGIS and PTGIR (Table S10), there are fundamentally distinct tumor-specific patterns. Transcript and total protein accumulation of master regulator ZBTB7A as well as PTGIS and PTGIR were reduced in UCES tumors, which was markedly related to the stage and prognosis of this tumor type [146]. Thus, the down-regulation of PTGIS and PTGIR in eight different tumors in our model may be due to the contribution of master regulators and oncomiR combinations at the transcriptional and post-transcriptional levels under conditions of unchanged promoter methylation status and copy number variations (deletion type) of target genes.

### Protein-Protein Interactions and Post-Translational Modifications

Since some of the prostanoids are quite metastable (short-lived) metabolites, spatial clustering or compartmentalization of prostanoid enzymes can be required. It is realized through either direct PPIs between enzymes or the involvement of common protein partners as well as post-translational modifications (PTMs). Previously, using the affinity purification and mass-spectrometry approach, we revealed that the PTGES3 protein could be a potential protein partner of PTGIS [147]. PPIs subnetworks with PTGIS and PTGES3 and their protein partners retrieved from STRINGdb are shown in Figure S3. Overrepresentation analysis (ORA) indicates that a subset of the PTGIS’s protein partners is enriched with steroid and cholesterol biosynthesis (FDFT1, HSD17B7, LSS, SC5D proteins) pathway terms. Functional “bridges” between cholesterol synthesis and prostanoid pathways (changes in prostacyclin levels in the presence of statins), as described in [148,149], can be mediated via modulation of *PTGIS* gene expression. But so far, we have not found studies that would evidence the functional value of PPIs with enzymes involved in prostanoid and cholesterol synthesis. The PTGES3′s protein partner subset is enriched with pathway terms such as “protein processing in endoplasmic reticulum”, “pathways in cancer” (KEGG); “cellular responses to external stimuli”, “aryl hydrocarbon receptor signaling” and “TNF alpha Signaling Pathway” (Reactome). In addition, comparing the data obtained in [147] and STRINGdb, we found that at least heat shock 70 kDa protein 4L (HSPA4L, Uniprot ID: O95757) and calreticulin (CALR, Uniprot ID: P27797) with chaperone activity are common protein partners of PTGIS and PTGES3 proteins.

**Figure 6:**
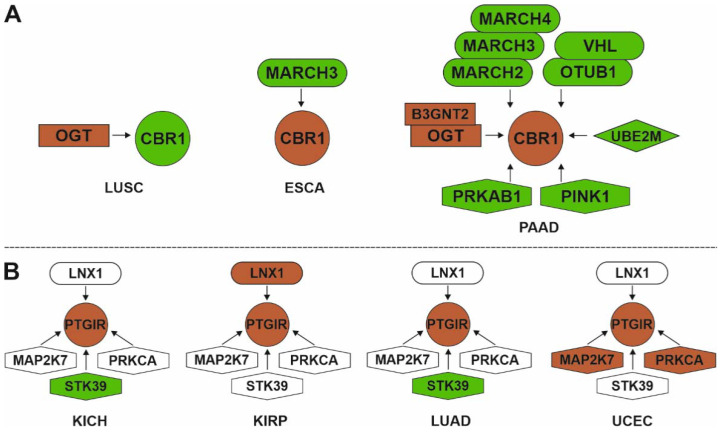
Associations between tumor-specific gene expression patterns of modifying enzymes and proteins in prostanoid signaling: (**A**)—CBR1; (**B**) PTGIR. Up- and down-regulated genes are highlighted with green and red colors, respectively. Ubiquitination enzymes, protein kinases, neddylation, and glycosylation enzymes are represented in oval, polygon, diamond, and rectangle shapes, respectively. Abbreviation: B3GNT2—(N-acetyllactosaminide beta-1,3-N-acetylglucosaminyltransferase 2); CBR1—(carbonyl reductase [NADPH] 1); LNX1—(E3 ubiquitin-protein ligase LNX); MAP2K7—(dual specificity mitogen-activated protein kinase 7); MARCH2—(E3 ubiquitin-protein ligase MARCHF2); MARCH3—(E3 ubiquitin-protein ligase MARCHF3); MARCH4—(E3 ubiquitin-protein ligase MARCHF4); OGT—(UDP-N-acetylglucosamine--peptide N-acetylglucosaminyltransferase 110 kDa subunit); OTUB1—(uUbiquitin thioesterase OTUB1); PINK1—(serine/threonine-protein kinase PINK1, mitochondrial); PRKAB1—(5′-AMP-activated protein kinase subunit beta-1); PTGIR—(prostacyclin receptor); STK39—(STE20/SPS1-related proline-alanine-rich protein kinase); UBE2M—(NEDD8-conjugating enzyme Ubc12); VHL—(von Hippel-Lindau disease tumor suppressor).

Amino acid sequences of prostanoid enzymes and receptors contain multiple sites for reversible post-translational modifications such as ubiquitination, phosphorylation/dephosphorylation, and glycosylation. In that context, the gene expression patterns of modifying proteins (ubiquitin ligases, protein kinases/phosphatases, and glycosyltransferases) were analyzed. The spectrum of potential modifying proteins, which physically interact with prostanoid enzymes and receptors, and their tumor-specific gene expression patterns are shown in Table S11. Up-regulation of ubiquitin-protein ligases SIAH2, MARCH2, MARCH3, UBE2W, OTUB1, and VHL, which regulate the stability of mature proteins, as well as the protein kinases STK24, MAP4K1, MAP4K4, PRKCD, CSK, PINK1, PRKAB1, and STK39, was found. It is known that mitogen-activated protein kinases (MAP4K1 and MAP4K4) and the insulin resistance pathway (PRKCD and PRKAB1) may be associated with tumor progression through modulation of gene expression responsible for cell cycle, proliferation, and growth [150]. We have schematically shown in Figure 6 the associations between gene expression patterns of CBR1 and PTGIR proteins and their modifying enzymes.

These examples demonstrate tumor-specific multiple modes of post-translational regulation for prostanoid enzymes and receptors, so the presence of “active combinations” of modifying enzymes depending on their expression levels in tumors can be suggested. However, the direct involvement of modifying proteins in post-translational modifications of target enzymes or receptors is still not sufficiently studied. It is known that PTGES protein stability is positively regulated through interaction with ubiquitin-specific peptidase 9 X-Linked (USP9X) [57]. Post-translational regulation via phosphorylation has been described for PTGES [151]. Prostanoid receptors have rather long cytoplasmic tails with potential phosphorylation sites [152], and protein kinase C-dependent phosphorylation has been described for the prostacyclin receptor [153].

The limitations of the study are related to the use of publicly available data from TCGA, CPTAC, and other repositories and web-based tools for the analysis of datasets. The results of the study have more fundamental rather than translational value. The identified transcriptional signatures, with the participation of prostanoid signaling genes with differential expression in tumor/normal tissues, are exploratory. Thus, to further establish the clinical relevance of such signatures, additional rounds of experimental validation should be required.

## 5. Conclusions

We investigated the highly heterogeneous gene and protein expression landscape of prostanoid enzymes and receptors in 24 different tumors and suggested the models of tumor-specific regulation. Nine bioinformatic web-based tools (GEPIA2, UALCAN, cBioPortal, hTFtarget, CSmirTar, ONCOmir, muTARGET, Biogrid, and ClustVis) were used for the analysis of differentially expressed genes, proteins, microRNAs, methylation and mutation patterns, as well as protein-protein interactions. Four groups of tumors were prioritized according to the profiling of the entire pool of differentially expressed target genes. The high correlation of co-expression was shown in the sub-cluster with *AKR1C3*, *CBR1*, and *CBR3* genes. Down-regulation of *PTGDR*, *PTGER3*, *PTGIR*, and *TBXA2R* genes in a number of tumors can be linked with promoter methylation status. Tissue-specific master regulators BRD4, CTCF, EP300, FOXA1, and SPI1, overexpressed in tumors, were found for target genes. Predicted microRNAs such as miR-149-3p, miR-19a-3p, miR-338-5p, miR-421, miR-423-5p, and miR-508-5p can be involved in the post-transcriptional regulation of at least two different target RNAs. The highest concordance between expression data of TCGA and CPTAC databases was achieved for *PTGIS* and *PTGES* genes in four tumors: BRCA, COAD, LUAD, and UCES. Mutation frequency of *TBXAS1*, *PTGIS*, *AKR1C3*, *PTGDR*, *PTGFR* and *PTGER3* genes in melanoma were 5–6%, 5–7%, 2.8–5%, 3–4%, 7–9%, 2.1–5%, respectively. One of the conclusions of the study is the assumption of the presence of prostanoid-dependent tumor phenotypes. It can be demonstrated by the total up-regulation of prostanoid synthesis enzymes in GBM, PAAD, and THYM tumors. Down-regulation of the *PTGIS* and *PTGIR* genes in eight different tumors may be associated with a more aggressive tumor phenotype due to the abolishment of prostacyclin’s tumor-suppressive effects. Models of CBR1 and PTGIR post-translational regulation models were mediated via neddylation and ubiquitination/deubiquitination as well as phosphorylation depending on tumor types. We have found several multigene cancer-specific transcriptomic signatures (in BLCA, CESK, SARC, LUAD, LIHC, and KIRP tumors) associated with patients’ overall survival rates (prognostic value). There are associations between the expression pattern of five target genes and the tumor response to cytostatic therapy. For example, differential expression of the PTGES gene was predictive in BRCA and OV tumors. From our point of view, for the first time, a systemic pan-cancer analysis of the molecular features of the expression of genes involved in prostanoid signaling was carried out. The new results obtained will be contributed to the insight into prostanoid signaling in a cancer context.

## Figures and Tables

**Table 1:** Tissue-specific master regulators of the expression of genes, encoding prostanoids enzymes and receptors in different cancers.

Cancer	Tissue-Specific Master Regulators
Breast cancer	***BRD4*** ▬, ***CTCF*** ▬, ***EP300*** ▬, ***FOXA1*** ▲▲, *SPI1* ▬
Brain cancer	*POLR2A*▬, *SPI1 *▲▲▲
Colorectal cancer	***CTCF*** ▬, *SP1*▬
Esophageal cancer	*KDM4C* ▬
Kidney cancer	*AR*▲, *RNF2*▬, *SPI1* ▲, *ZNF263*▬
Liver cancer	*FOXA2*▬, *HNF4A*▬, *MAX*▬
Lung cancer	*LMNB1*▲, *MAZ*▬, *RELA*▬, *SPI1* ▼
Pancreatic cancer	***CTCF*** ▲, *POLR2A*▬
Prostate cancer	*AR*▬, ***FOXA1*** ▲
Skin cancer	***CTCF*** ▬, *SPI1* ▲
Stomach cancer	*KLF5*▲▲▲, *SPI1* ▲
Uterine cancer	*NFIC*▼, *ZBTB7A* ▼

Note: Cancer driver genes and cancer hallmarks are highlighted with bold and underlined, respectively. Up- and down-regulated genes (1 < log2FC < 2, tumor/normal) are marked with arrows ▲ and ▼, respectively; 2 < log2FC < 3, ▲▲; log2FC > 3, ▲▲▲. No significant changes of gene expression (▬).

**Table 2:** A concordance between transcript and protein accumulation in tumors.

Tumor	BRCA		COAD		LUAD		OV		UCES	
Gene Names	TCGA	CPTAC	TCGA	CPTAC	TCGA	CPTAC	TCGA	CPTAC	TCGA	CPTAC
*TBXAS1*	▬	▬	▲	▲	▬	▬	▬	▬	▬	▬
*PTGIS*	▼	▼	▼	▼	▼	▼	▼	▼	▼	▼
*PTGDS*	▼	▼	▼	▼	▼	▼	▲	▼	▼	▼
*PTGES*	▬	▬	▬	▬	▲	▲	▬	▬	▬	▬
*PTGES3*	▬	▬	▲	▲	▬	▬	▬	▬	▬	▬
*PRXL2B*	▲	▬	▲	▼	▬	▬	▲	▬	▬	▬
*AKR1C3*	▼	▼	▬	▬	▬	▬	▼	▼	▬	▬
*CBR3*	▬	▬	▬	▬	▬	▬	▼	▼	▼	▼

▲ and▼, significant (*p*-value < 0.05) increase and decrease in transcript (TCGA datasets) or protein (CPTAC datasets) levels (tumor/normal tissue), respectively; ▬ no significant changes.

**Table 3:** Prognostic value of transcriptomics signatures of genes, encoding prostanoids enzymes and receptors.

Gene Expression Signature	Tumors, Subgroups	Hazard Ratio (CI), Logrank *p*-Value	Quartile, Survival (Months) Low-High Expression Cohorts	Signature Specificity Compared to Different Tumors
*PTGIS*, *PTGDS*, *PTGFR*, *PTGER3*	BLCA	2.1 (1.4–3.2), 9.2 × 10^−5^	Q1, 21–12	KIRP
	BLCA, male gender	2.5 (1.6–4.0), 7.3 × 10^−5^	Q1, 22–12	not found
	BLCA, stage 3	2.2 (1.0–4.7), 0.034	Q1, 21–13	not found
*PTGDS*, *CBR3*, *PTGIR* *PTGFR*, *PTGDR2*, *PTGER3*	HNSC	0.6 (0.4–0.8), 0.00032	Q1, 26–59	BRCA, CESC, LUAD, SARC, UCES
	HNSC, male gender, high mutation burden	0.4 (0.2–0.8), 0.0058	Q1, 13–47	not found
	HNSC, male gender, low mutation burden	0.5 (0.3–0.8), 0.0031	Q1, 12–23	not found
	HNSC, stage 3	0.2 (0.1–0.6), 0.00058	Q1, 11–57	not found
*PTGDS*, *AKR1C3*, *CBR1*, *CBR3*, *PTGDR*, *PTGDR2*, *PTGER2*, *PTGER4*	CESK	0.5 (0.3–0.8), 0.0067	Median, NA–NA	SARC
	CESK, female gender, white race	0.4 (0.2–0.8), 0.0027	Q1, 21–42	not found
*TBXAS1*, *PTGDS*, *AKR1C3*, *PTGIS*, *CBR3*, *TBXA2R*, *PTGDR*, *PTGFR*, *PTGER3*, *PTGER4*	SARC	0.5 (0.3–0.7), 00026	Q1, 16–37	BRCA, CESK,
	SARC, high mutation burden	0.4 (0.2–0.7), 0.00043	Q1, 11–37	not found
	SARC, low mutation burden	0.4 (0.2–0.7), 0.0023	Q1, 17–41	not found
*PTGER1*, *PTGER3*, *PTGER4*	UCES	2.3 (1.5–3.5), 0.00012	Median, NA–NA	not found
	UCES, grade 3	2.0 (1.2–3.3), 0.0075	Q1, 60–29	not found
*PTGDS*, *PTGDR2*	LUAD	0.4 (0.3–0.6), 4 × 10^−5^	Q1, 21–42	CESK, SARC
*TBXAS1*, *PTGDS*, *AKR1C3*, *PTGIS*, *CBR3*, *TBXA2R*, *PTGDR*, *PTGFR*, *PTGER3*, *PTGER4*	LIHC	2.2 (1.5–3.1), 1.5 × 10^−5^	Q1, 28–10	KIRP
*PTGES2*, *PTGES3*, *PRXL2B*, *AKR1C3*, *PTGES*	LIHC	2.2 (1.5–3.1), 1.3 × 10^−5^	Q1, 27–11	LUAD, PAAD
	LIHC, female gender	2.7 (1.5–5.0), 0.00059	Q1, 30–9	not found
	LIHC, male gender	2.4 (1.5–3.7), 9.2 × 10^−5^	Q1, 28–10	not found
	LIHC, grade 3	2.9 (1.5–5.8), 0.0014	Q1, 50–12	not found
	LIHC, high mutation burden	2.7 (1.6–4.4), 5.8 × 10^−5^	Q1, 60–14	not found
	LIHC, low mutation burden	2.0 (1.1–3.4), 0.015	Q1, 26–20	not found
*PTGIS*, *PTGDS*, *PTGES3*, *AKR1C3*, *CBR3*, *TBXA2R*, *PTGDR*, *PTGER1*, *PTGFR*, *PTGER3*	KIRP	5.1 (2.7–9.7), 3.4 × 10^−10^	Median, NA–NA	not found
	KIRP, low mutation burden	10.1 (3.6–28.3), 5.4 × 10^−8^	Median, NA–NA	not found

## Data Availability

Not applicable.

## Supplementary Materials

The following supporting information can be downloaded at: https://www.mdpi.com/article/10.3390/biology11040590/s1, Table S1. The associations of prostanoid metabolizing enzymes and prostanoid receptors with neoplastic transformation. Table S2. Cell lines dependency on target genes’ knockouts and knockdowns. Table S3. Over-representation analysis of genes showing similar expression patterns with target genes involved in prostanoid signaling. Table S4. Concordance between changes of gene expression and promoter methylation patterns. Table S5. Over-representation analysis of master regulators using the KEGG database as a data source. Table S6. A list of predicted regulatory oncomiRs for target genes. Table S7. Expression ratio (tumor/normal) of predicted master oncomiRs that can regulate mRNAs of genes involved in prostanoid signaling. Table S8. Antibody staining of proteins involved in prostanoid signaling according to the Human Proteome Atlas portal. Table S9. Prediction of genes, which expression patterns can be associated with genetic polymorphism in target genes in melanoma tumors. Table S10. Cancer-specific expression and regulation patterns of prostanoid-metabolizing enzymes and receptors. Table S11. Gene expression changes by modifying proteins retrieved from the Biogrid database and physically interacting with the proteins involved in prostanoid signaling. Figure S1. Kaplan-Meier plots: overall survival vs. expression levels of target genes in several tumors. Figure S2. Predictive value of gene expression of prostanoid-metabolizing enzymes and prostanoid receptors in breast (Figure S2A) and ovarian (Figure S2B) cancers. Figure S3. Protein-protein interaction subnetworks with PTGIS and PTGES3 and their physically interacting protein partners sharing similar subcellular localization patterns with target proteins. Edges selected with dotted dashed, and solid lines indicate 0.4–0.5, 0.5–0.7, and 0.7–0.99 STRINGdb combined scores (experimentally_determined_interactions), respectively.

## Abbreviations

TXA_2_	thromboxane A_2_
TXB_2_	thromboxane B_2_
PGE_2_	prostaglandin E_2_
PGF_2α_	prostaglandin F_2α_
PGD_2_	prostaglandin D_2_
PGI_2_	Prostacyclin
PGH_2_	prostaglandin H_2_
*TBXAS1*	thromboxane A synthase 1
*PTGIS*	prostaglandin I_2_ synthase
*PTGDS*	prostaglandin D_2_ synthase
*PTGES*	prostaglandin E synthase
*PTGES2*	prostaglandin E synthase 2
*PTGES3*	prostaglandin E synthase 3
*PRXL2B*	peroxiredoxin like 2B
*AKR1C3*	aldo-keto reductase family 1 member C3
*CBR1*	carbonyl reductase 1
*CBR3*	carbonyl reductase 3
*TBXA2R*	thromboxane A_2_ receptor
*PTGIR*	prostaglandin I_2_ receptor
*PTGDR*	prostaglandin D_2_ receptor
*PTGFR*	prostaglandin F receptor
*PTGDR2*	prostaglandin D_2_ receptor 2
*PTGER1*	prostaglandin E receptor 1
*PTGER2*	prostaglandin E receptor 2
*PTGER3*	prostaglandin E receptor 3
*PTGER4*	prostaglandin E receptor 4
Gq α	Gq protein alpha subunit
Gs α	Gs protein alpha subunit
Gi α	Gi protein alpha subunit
Gi/12 α	Gi & G12 protein subunit
ACC	adrenocortical carcinoma
BLCA	bladder urothelial carcinoma
BRCA	breast invasive carcinoma
CESC	cervical squamous cell carcinoma and endocervical adenocarcinoma
CHOL	cholangiocarcinoma
COAD	colon adenocarcinoma
DLBC	lymphoid neoplasm diffuse large B-cell lymphoma
ESCA	esophageal carcinoma
GBM	glioblastoma multiforme
HNSC	head and neck squamous cell carcinoma
KICH	kidney chromophobe renal cell carcinoma
KIRC	kidney renal clear cell carcinoma
KIRP	kidney renal papillary cell carcinoma
LAML	scute myeloid leukemia
LGG	brain lower grade glioma
LIHC	liver hepatocellular carcinoma
LUAD	lung adenocarcinoma
LUSC	lung squamous cell carcinoma
MESO	mesothelioma
OV	ovarian serous cystadenocarcinoma
PAAD	pancreatic adenocarcinoma
PCPG	pheochromocytoma and paraganglioma
PRAD	prostate adenocarcinoma,
READ	rectum adenocarcinoma
SARC	sarcoma
SKCM	skin cutaneous melanoma
STAD	stomach adenocarcinoma
TGCT	testicular germ cell tumors
THCA	thyroid carcinoma
THYM	thymoma
UCEC	uterine corpus endometrial carcinoma
UCS	uterine carcinosarcoma
UVM	uveal melanoma

## Appendix A

**Table A1:** Literature reports on the involvement of prostanoids in neoplastic transformation.

Prostanoids	Description	References
TXA_2_	TXA_2_ impacts the interface of platelet-tumor cell crosstalk and serves as a link between platelets in ovarian cancer.	[20]
TXA_2_	Inhibition of TXA_2_ synthesis reduced human umbilical vein endothelial cells migration stimulated by VEGF or bFGF. The development of lung metastasis in mice models was significantly inhibited by thromboxane synthase inhibitors.	[21]
TXB_2_	High TXB_2_ urinary level was associated with (i) prostate cancer in African American men (OR 1.50, 1.13–2.00), but not European American men (OR 1.07, 0.82–1.40); (ii) metastatic prostate cancer (OR 2.60, 1.08–6.28) compared with low levels of TXB_2_.	[22]
TXB_2_	TXB_2_ was much higher in the non-small cell lung carcinoma tissue than in normal tissues and advanced-stage cancers had higher levels of TXB_2_ thus supporting the role of TXB_2_ in tumor growth promotion.	[23]
11-dihydro-TXB_2_	In 10 patients with colorectal cancer, the urinary excretion of 11-dehydro-TXB_2_ was significantly higher than in 10 controls. Enhanced platelet activation occurs in colorectal cancer patients and low-dose aspirin might restore anti-tumor reactivity.	[24]
PGE_2_	PGE_2_ promotes gastrointestinal tumor progression and metastasis by (i) direct effect on tumor cell proliferation, survival, and migration/invasion; (ii) tumor-associated immunosuppression; (iii) by silencing certain tumor suppressor and DNA repair genes via DNA methylation.	[25]
PGE_2_	PGE_2_ promotes resistance to apoptosis, metastasis, angiogenesis, and drug resistance in colon cancer. Increased levels of PGE_2_ are associated with cancer progression. Pharmacology targeting PGE_2_ receptors may be a potent therapeutic anti-cancer strategy.	[26]
PGF_2α_	13,14-dihydro-15-keto PGF_2α_ was significantly reduced in type II endometrial cancer (EC) compared with normal endometrium, however, PGF_2α_ level increased in case of endometrium hyperplasia.	[27]
PGF_2α_	Urinary 8-epi-PGF_2α_ levels were correlated with tumor histologic subtype of ovarian cancer.	[28]
PGF_2α_	Serum 8-iso-PGF_2α_ showed high diagnostic performance in breast cancer (AUC = 0.99, sensitivity = 100%, specificity = 99% at a cutoff value of 36 pg/mL) thus providing evidence that the high level of serum 8-iso-PGF_2α_ helps to distinguish breast cancer and benign tumors (*p* < 0.001).	[29]
PGF_2α_	Urinary 8-iso-PGF_2α_ and 2,3-dinor-8-iso-PGF_2α_ were increased in the carcinogenesis phase of colitis-associated colon cancer.	[30]
TXB_2_, PGD_2_, PGE_2_, PGF_2α_	Glioblastomas had higher concentrations of TXB_2_, PGD_2_, PGE_2_, and PGF_2α_ versus grade II/III tumors. A significant decrease in survival rates was correlated with high levels of PGE_2_ and PGF_2α_ in the tumor.	[31]
PGF_1α_-iso-prostanoids, TXB_2_	Peripheral plasma levels of 6-keto-PGF_1α_ and TXB_2_ were higher in patients with breast malignant tumors than in healthy controls. The high levels of 6-keto-PGF_1α_ and TXB_2_ did not correlate with clinical and histopathological data.	[32]
PGF_1α_-iso-prostanoids	Patients with ovarian cancer excreted increased amounts of urinary 6-keto-PGF_1α_ with no relation to tumor histology or stage.	[33]
PGA_2_, PGB_2_, PGE_1_, PGE_2_, TXB_2_, PGD_2_, PGI_2_, 6-keto PGF_1α_	Higher levels of PGA_2_, PGB_2_, PGE_1_, PGE_2_, and TXB_2_ were observed in muscle invasive bladder cancer in contrast to both normal urothelium and non-MIBC, whereas PGD_2_, PGI_2_, and 6-keto PGF_1α_ were decreased in urothelial carcinoma. That points to different implications in cancer of up-regulated cyclooxygenase, PTGES and TBXAS,1 and down-regulated PTGDS as well as PTGIS.	[34]
PGI_2_	Iloprost, a stable PGI_2_ analog, inhibited migration and invasion of ovarian cancer cells as well as downregulated the expression of metastasis-associated matrix metallopeptidase-2 and -9 (MMP-2 and MMP-9) via the prostacyclin receptor-mediated protein kinase A pathway.	[35]
PGI_2_	Hyperproduction of intracellular PGI2 promotes apoptosis by activating peroxisome proliferator-activated receptor δ (PPARδ), acting as a second signaling pathway that controls cell apoptosis.	[36]
PGD_2_	Signaling between PGD_2_ and PTGDR2 has the ability to restrict the self-renewal of gastric cancer cells in vitro and suppress tumor growth and metastasis in vivo. The study showed a novel function of PGD_2_/PTGDR2 signaling in cancer stem cells regulation that is critical for tumor neovascularization and invasiveness.	[37]

## References

[1] Bos C.L., Richel D.J., Ritsema T., Peppelenbosch M., Versteeg H. (2004). Prostanoids and prostanoid receptors in signal transduction. Int. J. Biochem. Cell Biol..

[2] Zhu L., Zhang Y., Guo Z., Wang M. (2020). Cardiovascular Biology of Prostanoids and Drug Discovery. Arter. Thromb. Vasc. Biol..

[3] Iñiguez M.A., Cacheiro-Llaguno C., Cuesta N., Díaz-Muñoz M.D., Fresno M. (2008). Prostanoid function and cardiovascular disease. Arch. Physiol. Biochem..

[4] Rahnama’I M.S., Van Kerrebroeck P.E.V., De Wachter S.G., Van Koeveringe G.A. (2012). The role of prostanoids in urinary bladder physiology. Nat. Rev. Urol..

[5] Jara-Gutiérrez Á., Baladrón V. (2021). The Role of Prostaglandins in Different Types of Cancer. Cells.

[6] Zhao S., Cheng C.K., Zhang C.-L., Huang Y. (2021). Interplay between Oxidative Stress, Cyclooxygenases, and Prostanoids in Cardiovascular Diseases. Antioxid. Redox Signal..

[7] Wang B., Wu L., Chen J., Dong L., Chen C., Wen Z., Hu J., Fleming I., Wang D.W. (2021). Metabolism pathways of arachidonic acids: Mechanisms and potential therapeutic targets. Signal Transduct. Target. Ther..

[8] Diakowska D., Markocka-Mączka K., Nienartowicz M., Lewandowski A., Grabowski K. (2014). Increased level of serum prostaglandin-2 in early stage of esophageal squamous cell carcinoma. Arch. Med. Sci..

[9] D’Eufemia P., Finocchiaro R., Celli M., Zambrano A., Tetti M., Villani C., Persiani P., Mari E., Zicari A. (2008). High Levels of Serum Prostaglandin E2 in Children with Osteogenesis Imperfecta Are Reduced by Neridronate Treatment. Pediatr. Res..

[10] Mitchell M.D., Flint A.P., Bibby J., Brunt J., Arnold J.M., Anderson A.B.M., Aturnbull A.C. (1978). Plasma Concentrations of Prostaglandins during Late Human Pregnancy: Influence of Normal and Preterm Labor. J. Clin. Endocrinol. Metab..

[11] Brun C., Daali Y., Combescure C., Zufferey A., Michelson A.D., Fontana P., Reny J.-L., Iii A.L.F. (2016). Aspirin response: Differences in serum thromboxane B2 levels between clinical studies. Platelets.

[12] Gachet M.S., Rhyn P., Bosch O.G., Quednow B.B., Gertsch J. (2015). A quantitiative LC-MS/MS method for the measurement of arachidonic acid, prostanoids, endocannabinoids, N-acylethanolamines and steroids in human plasma. J. Chromatogr. B.

[13] Kanehisa M., Goto S. (2000). KEGG: Kyoto Encyclopedia of Genes and Genomes. Nucleic Acids Res..

[14] Nakanishi T., Nakamura Y., Umeno J. (2021). Recent advances in studies of SLCO2A1 as a key regulator of the delivery of prostaglandins to their sites of action. Pharmacol. Ther..

[15] Norel X., Sugimoto Y., Ozen G., Abdelazeem H., Amgoud Y., Bouhadoun A., Bassiouni W., Goepp M., Mani S., Manikpurage H.D. (2020). International Union of Basic and Clinical Pharmacology. CIX. Differences and Similarities between Human and Rodent Prostaglandin E2Receptors (EP1–4) and Prostacyclin Receptor (IP): Specific Roles in Pathophysiologic Conditions. Pharmacol. Rev..

[16] Biringer R.G. (2021). A Review of Prostanoid Receptors: Expression, Characterization, Regulation, and Mechanism of Action. J. Cell Commun. Signal..

[17] Kobsar A.L., Koessler J., Rajkovic M.S., Brunner K.P., Steigerwald U., Walter U. (2010). Prostacyclin receptor stimulation facilitates detection of human platelet P2Y(12)receptor inhibition by the PFA-100 system. Platelets.

[18] Wang D., Cabalag C.S., Clemons N.J., DuBois R.N. (2021). Cyclooxygenases and Prostaglandins in Tumor Immunology and Microenvironment of Gastrointestinal Cancer. Gastroenterology.

[19] Cathcart M.-C., Reynolds J.V., O’Byrne K.J., Pidgeon G.P. (2010). The role of prostacyclin synthase and thromboxane synthase signaling in the development and progression of cancer. Biochim. Biophys. Acta.

[20] Serhan K., Gartung A., Panigrahy D. (2018). Drawing a link between the thromboxane A2 pathway and the role of platelets and tumor cells in ovarian cancer. Prostaglandins Other Lipid Mediat..

[21] Nie D., Lamberti M., Zacharek A., Li L., Szekeres K., Tang K., Chen Y., Honn K.V. (2000). Thromboxane A2 Regulation of Endothelial Cell Migration, Angiogenesis, and Tumor Metastasis. Biochem. Biophys. Res. Commun..

[22] Kiely M., Milne G.L., Minas T.Z., Dorsey T.H., Tang W., Smith C.J., Baker F., Loffredo C.A., Yates C., Cook M.B. (2021). Urinary Thromboxane B2 and Lethal Prostate Cancer in African American Men. JNCI J. Natl. Cancer Inst..

[23] Chen G.G., Lee T.W., Yip J.H., Xu H., Lee I.K., Mok T.S., Warner T.D., Yim A.P. (2006). Increased thromboxane B2 levels are associated with lipid peroxidation and Bcl-2 expression in human lung carcinoma. Cancer Lett..

[24] Sciulli M., Filabozzi P., Tacconelli S., Padovano R., Ricciotti E., Capone M., Grana M., Carnevale V., Patrignani P. (2005). Platelet activation in patients with colorectal cancer. Prostaglandins Leukot. Essent. Fat. Acids.

[25] Wang D., Dubois R.N. (2018). Role of prostanoids in gastrointestinal cancer. J. Clin. Investig..

[26] Karpisheh V., Nikkhoo A., Farsangi M.H., Namdar A., Azizi G., Ghalamfarsa G., Sabz G., Yousefi M., Yousefi B., Jadidi-Niaragh F. (2019). Prostaglandin E2 as a potent therapeutic target for treatment of colon cancer. Prostaglandins Other Lipid Mediat..

[27] Cummings M., Massey K.A., Mappa G., Wilkinson N., Hutson R., Munot S., Saidi S., Nugent D., Broadhead T., Wright A.I. (2019). Integrated eicosanoid lipidomics and gene expression reveal decreased prostaglandin catabolism and increased 5-lipoxygenase expression in aggressive subtypes of endometrial cancer. J. Pathol..

[28] Caglayan A., Katlan D.C., Selcuk Tuncer Z., Yüce K., Sayal H.B., Coskun Salman M., Kocer-Gumusel B. (2017). Impaired antioxidant enzyme functions with increased lipid peroxidation in epithelial ovarian cancer. IUBMB Life.

[29] Eldin E.E.M.N., Eldein M.M.N., El-Readi M.Z., Mirza A.A., Fatani S.H., Al-Amodi H.S., Althubiti M.A., Al-Ezzi E.M., Eid S.Y., Kamel H.F.M. (2020). Evaluation of the Diagnostic and Predicative Values of 8-Iso-Prostaglandin F2α as a Biomarker of Breast Cancer. Oncol. Res. Treat..

[30] Miyazaki Y., Nakamura T., Takenouchi S., Hayashi A., Omori K., Murata T. (2021). Urinary 8-iso PGF2α and 2,3-dinor-8-iso PGF2α can be indexes of colitis-associated colorectal cancer in mice. PLoS ONE.

[31] Panagopoulos A.T., Gomes R.N., Almeida F.G., Souza F.D.C., Veiga J.C.E., Nicolaou A., Colquhoun A. (2018). The prostanoid pathway contains potential prognostic markers for glioblastoma. Prostaglandins Other Lipid Mediat..

[32] Nigam S., Becker R., Rosendahl U., Hammerstein J., Benedetto C., Barbero M., Slater T. (1985). The concentration of 6-keto-PGF1α and TXB2 in plasma samples from patients with benign and malignant tumours of the breast. Prostaglandins.

[33] Aitokallio-Tallberg A., Viinikka L., Ylikorkala O. (1987). Urinary 6-keto-prostaglandin F1a in patients with gynaecological tumours. Cancer Lett..

[34] Sahu D., Lotan Y., Wittmann B., Neri B., Hansel D.E. (2017). Metabolomics analysis reveals distinct profiles of nonmuscle-invasive and muscle-invasive bladder cancer. Cancer Med..

[35] Ahn J.-H., Lee K.-T., Choi Y.S., Choi J.-H. (2018). Iloprost, a prostacyclin analog, inhibits the invasion of ovarian cancer cells by downregulating matrix metallopeptidase-2 (MMP-2) through the IP-dependent pathway. Prostaglandins Other Lipid Mediat..

[36] Hatae T., Wada M., Yokoyama C., Shimonishi M., Tanabe T. (2001). Prostacyclin-dependent Apoptosis Mediated by PPAR Delta. J. Biol. Chem..

[37] Zhang B., Bie Q., Wu P., Zhang J., You B., Shi H., Qian H., Xu W. (2018). PGD2/PTGDR2 Signaling Restricts the Self-Renewal and Tumorigenesis of Gastric Cancer. Stem Cells.

[38] Piñero J., Bravo À., Queralt-Rosinach N., Gutiérrez-Sacristán A., Deu-Pons J., Centeno E., García-García J., Sanz F., Furlong L.I. (2016). DisGeNET: A comprehensive platform integrating information on human disease-associated genes and variants. Nucleic Acids Res..

[39] Deng Q.-P., Wang M.-J., Zeng X., Chen G.G., Huang R.-Y. (2017). Effects of Glycyrrhizin in a Mouse Model of Lung Adenocarcinoma. Cell. Physiol. Biochem..

[40] Liu Y., Yang S., Li M.-Y., Huang R., Ng C.S., Wan I.Y., Long X., Wu J., Wu B., Du J. (2015). Tumorigenesis of smoking carcinogen 4-(methylnitrosamino)-1-(3-pyridyl)-1-butanone is related to its ability to stimulate thromboxane synthase and enhance stemness of non-small cell lung cancer stem cells. Cancer Lett..

[41] Cathcart M.C., Gately K., Cummins R., Drakeford C., Kay E.W., O’Byrne K.J., Pidgeon G.P. (2014). Thromboxane synthase expression and correlation with VEGF and angiogenesis in non-small cell lung cancer. Biochim. Biophys. Acta.

[42] Li H., Lee M.-H., Liu K., Wang T., Song M., Han Y., Yao K., Xie H., Zhu F., Grossmann M. (2017). Inhibiting breast cancer by targeting the thromboxane A2 pathway. NPJ Precis. Oncol..

[43] Sasaki Y., Ochiai T., Takamura M., Kondo Y., Yokoyama C., Hara S. (2017). Role of prostacyclin synthase in carcinogenesis. Prostaglandins Other Lipid Mediat..

[44] Li H.Y., McSharry M., Walker D., Johnson A., Kwak J., Bullock B., Neuwelt A., Poczobutt J.M., Sippel T.R., Keith R.L. (2018). Targeted overexpression of prostacyclin synthase inhibits lung tumor progression by recruiting CD4+ T lymphocytes in tumors that express MHC class II. OncoImmunology.

[45] Kwon O.K., Ha Y.-S., Na A.-Y., Chun S.Y., Kwon T.G., Lee J.N., Lee S. (2020). Identification of Novel Prognosis and Prediction Markers in Advanced Prostate Cancer Tissues Based on Quantitative Proteomics. Cancer Genom. Proteom..

[46] Lichao S., Liang P., Chunguang G., Fang L., ZhiHua Y., Yuliang R. (2011). Overexpression of PTGIS Could Predict Liver Metastasis and is Correlated with Poor Prognosis in Colon Cancer Patients. Pathol. Oncol. Res..

[47] Jin R., Strand D.W., Forbes C.M., Case T., Cates J.M.M., Liu Q., Ramirez-Solano M., Milne G.L., Bs S.S., Wang Z.Y. (2021). The prostaglandin pathway is activated in patients who fail medical therapy for benign prostatic hyperplasia with lower urinary tract symptoms. Prostate.

[48] Zou R., Zheng M., Tan M., Xu H., Luan N., Zhu L. (2020). Decreased PTGDS Expression Predicting Poor Survival of Endometrial Cancer by Integrating Weighted Gene Co-Expression Network Analysis and Immunohistochemical Validation. Cancer Manag. Res..

[49] He L.P., Chen Y.F., Yang J. (2017). Investigation on the role and mechanism of prostagland in D2 synthase in non-small cell lung cancer. Zhonghua Yi Xue Za Zhi.

[50] Pan J., Zhang L., Huang J. (2021). Prostaglandin D2 synthase/prostaglandin D2/TWIST2 signaling inhibits breast cancer proliferation. Anti Cancer Drugs.

[51] Hu S., Ren S., Cai Y., Liu J., Han Y., Zhao Y., Yang J., Zhou X., Wang X. (2021). Glycoprotein PTGDS promotes tumorigenesis of diffuse large B-cell lymphoma by MYH9-mediated regulation of Wnt-β-catenin-STAT3 signaling. Cell Death Differ..

[52] Jiang P., Cao Y., Gao F., Sun W., Liu J., Ma Z., Xie M., Fu S. (2021). SNX10 and PTGDS are associated with the progression and prognosis of cervical squamous cell carcinoma. BMC Cancer.

[53] Bie Q., Li X., Liu S., Yang X., Qian Z., Zhao R., Zhang X., Zhang B. (2020). YAP promotes self-renewal of gastric cancer cells by inhibiting expression of L-PTGDS and PTGDR2. Int. J. Clin. Oncol..

[54] Liu C., Yang J.C., Armstrong C.M., Lou W., Liu L., Qiu X., Zou B., Lombard A.P., D’Abronzo L.S., Evans C.P. (2019). AKR1C3 Promotes AR-V7 Protein Stabilization and Confers Resistance to AR-Targeted Therapies in Advanced Prostate Cancer. Mol. Cancer Ther..

[55] Penning T.M. (2019). AKR1C3 (type 5 17β-hydroxysteroid dehydrogenase/prostaglandin F synthase): Roles in malignancy and endocrine disorders. Mol. Cell. Endocrinol..

[56] Russo A., Biselli-Chicote P.M., Kawasaki-Oyama R.S., Castanhole-Nunes M.M., Maniglia J.V., Neto D.D.S., Pavarino É.C., Goloni-Bertollo E.M. (2018). Differential Expression of Prostaglandin I2 Synthase Associated with Arachidonic Acid Pathway in the Oral Squamous Cell Carcinoma. J. Oncol..

[57] Wang T., Jing B., Sun B., Liao Y., Song H., Xu D., Guo W., Li K., Hu M., Liu S. (2019). Stabilization of PTGES by deubiquitinase USP9X promotes metastatic features of lung cancer via PGE2 signaling. Am. J. Cancer Res..

[58] Nagaraja A., Dorniak P.L., Sadaoui N.C., Kang Y., Lin T., Armaiz-Pena G.N., Wu S.Y., Rupaimoole R., Allen J.K., Gharpure K. (2016). Sustained adrenergic signaling leads to increased metastasis in ovarian cancer via increased PGE2 synthesis. Oncogene.

[59] Wang T., Jing B., Xu D., Liao Y., Song H., Sun B., Guo W., Xu J., Li K., Hu M. (2020). PTGES/PGE2 signaling links immunosuppression and lung metastasis in Gprc5a-knockout mouse model. Oncogene.

[60] Stamatakis K., Jimenez-Martinez M., Jimenez-Segovia A., Chico-Calero I., Conde-Moreno E., Martínez J.G., Ruiz J., Pascual A., Barrocal B., López B.B. (2015). Prostaglandins induce early growth response 1 transcription factor mediated microsomal prostaglandin E2 synthase up-regulation for colorectal cancer progression. Oncotarget.

[61] Kim S.-H., Roszik J., Cho S.-N., Ogata D., Milton D.R., Peng W., Menter D.G., Ekmekcioglu S., Grimm E.A. (2019). The COX2 Effector Microsomal PGE2 Synthase 1 is a Regulator of Immunosuppression in Cutaneous Melanoma. Clin. Cancer Res..

[62] Kim S., Lee E.S., Jung J.Y., Lee S.B., Lee H.J., Kim J., Kim H.J., Lee J.W., Son B.H., Gong G. (2021). Targeted eicosanoids profiling reveals a prostaglandin reprogramming in breast Cancer by microRNA-155. J. Exp. Clin. Cancer Res..

[63] Garo L.P., Ajay A.K., Fujiwara M., Gabriely G., Raheja R., Kuhn C., Kenyon B., Skillin N., Kadowaki-Saga R., Saxena S. (2021). MicroRNA-146a limits tumorigenic inflammation in colorectal cancer. Nat. Commun..

[64] Ke J., Shen Z., Li M., Peng C., Xu P., Wang M., Zhu Y., Zhang X., Wu D. (2018). Prostaglandin E2 triggers cytochrome P450 17α hydroxylase overexpression via signal transducer and activator of transcription 3 phosphorylation and promotes invasion in endometrial cancer. Oncol. Lett..

[65] Kong J., Shen S., Zhang Z., Wang W. (2020). Identification of hub genes and pathways in cholangiocarcinoma by coexpression analysis. Cancer Biomark..

[66] Gu Y., Chen G., Du Y. (2020). Screening of Prognosis-Related Genes in Primary Breast Carcinoma Using Genomic Expression Data. J. Comput. Biol..

[67] Zhou L., Yang C., Zhong W., Wang Q., Zhang D., Zhang J., Xie S., Xu M. (2021). Chrysin induces autophagy-dependent ferroptosis to increase chemosensitivity to gemcitabine by targeting CBR1 in pancreatic cancer cells. Biochem. Pharmacol..

[68] Osawa Y., Yokoyama Y., Shigeto T., Futagami M., Mizunuma H. (2014). Decreased expression of carbonyl reductase 1 promotes ovarian cancer growth and proliferation. Int. J. Oncol..

[69] Yun M., Choi A.J., Woo S.R., Noh J.K., Sung J.-Y., Lee J.-W., Eun Y.-G. (2020). Inhibition of Carbonyl Reductase 1 Enhances Metastasis of Head and Neck Squamous Cell Carcinoma through β-catenin-Mediated Epithelial-Mesenchymal Transition. J. Cancer.

[70] Yamanouchi R., Harada K., Ferdous T., Ueyama Y. (2018). Low carbonyl reductase 1 expression is associated with poor prognosis in patients with oral squamous cell carcinoma. Mol. Clin. Oncol..

[71] Zhang M., Wang Y., Jiang L., Song X., Zheng A., Gao H., Wei M., Zhao L. (2021). LncRNA CBR3-AS1 regulates of breast cancer drug sensitivity as a competing endogenous RNA through the JNK1/MEK4-mediated MAPK signal pathway. J. Exp. Clin. Cancer Res..

[72] Liu S., Zhan N., Gao C., Xu P., Wang H., Wang S., Piao S., Jing S. (2021). Long noncoding RNA CBR3-AS1 mediates tumorigenesis and radiosensitivity of non-small cell lung cancer through redox and DNA repair by CBR3-AS1 /miR-409-3p/SOD1 axis. Cancer Lett..

[73] Zhou Q., Ding W., Weng Y., Ding G., Xia G., Xu J., Xu K., Ding Q. (2018). NOS3 895G>T and CBR3 730G>A Are Associated with Recurrence Risk in Non-Muscle-Invasive Bladder Cancer with Intravesical Instillations of THP. Chemotherapy.

[74] Misawa K., Mima M., Satoshi Y., Imai A., Mochizuki D., Ishikawa R., Kita J., Yamaguchi Y., Endo S., Misawa Y. (2020). Prostanoid receptor genes confer poor prognosis in head and neck squamous cell carcinoma via epigenetic inactivation. J. Transl. Med..

[75] Misawa K., Imai A., Kanazawa T., Mima M., Yamada S., Mochizuki D., Yamada T., Shinmura D., Ishikawa R., Kita J. (2020). G Protein-Coupled Receptor Genes, PTGDR1, PTGDR2, and PTGIR, are Candidate Epigenetic Biomarkers and Predictors for Treated Patients with HPV-Associated Oropharyngeal Cancer. Microorganisms.

[76] O’Sullivan A.G., Mulvaney E.P., Kinsella B.T. (2017). Regulation of protein kinase C-related kinase (PRK) signalling by the TPα and TPβ isoforms of the human thromboxane A 2 receptor: Implications for thromboxane- and androgen- dependent neoplastic and epigenetic responses in prostate cancer. Biochim. Biophys. Acta.

[77] Werfel T.A., Hicks D.J., Rahman B., Bendeman W.E., Duvernay M.T., Maeng J.G., Hamm H., Lavieri R.R., Joly M.M., Pulley J.M. (2020). Repurposing of a Thromboxane Receptor Inhibitor Based on a Novel Role in Metastasis Identified by Phenome-Wide Association Study. Mol. Cancer Ther..

[78] Orr K., Buckley N., Haddock P., James C., Parent J.-L., McQuaid S., Mullan P.B. (2016). Thromboxane A2 receptor (TBXA2R) is a potent survival factor for triple negative breast cancers (TNBCs). Oncotarget.

[79] Mulvaney E.P., Shilling C., Eivers S.B., Perry A.S., Bjartell A., Kay E.W., Watson R.W., Kinsella B.T. (2016). Expression of the TPα and TPβ isoforms of the thromboxane prostanoid receptor (TP) in prostate cancer: Clinical significance and diagnostic potential. Oncotarget.

[80] Dash P., Ghatak S., Topi G., Satapathy S.R., Ek F., Hellman K., Olsson R., Mehdawi L.M., Sjölander A. (2021). High PGD2 receptor 2 levels are associated with poor prognosis in colorectal cancer patients and induce VEGF expression in colon cancer cells and migration in a zebrafish xenograft model. Br. J. Cancer.

[81] Lv J., Li L. (2019). Hub Genes and Key Pathway Identification in Colorectal Cancer Based on Bioinformatic Analysis. Biomed Res. Int..

[82] Fu C., Mao W., Gao R., Deng Y., Gao L., Wu J., Zhang S., Shen Y., Liu K., Li Q. (2020). Prostaglandin F2α-PTGFR signaling promotes proliferation of endometrial epithelial cells of cattle through cell cycle regulation. Anim. Reprod. Sci..

[83] Alkhateeb A., Rezaeian I., Singireddy S., Cavallo-Medved D., Porter L.A., Rueda L. (2019). Transcriptomics Signature from Next-Generation Sequencing Data Reveals New Transcriptomic Biomarkers Related to Prostate Cancer. Cancer Inform..

[84] Akiyama K., Ohga N., Maishi N., Hida Y., Kitayama K., Kawamoto T., Osawa T., Suzuki Y., Shinohara N., Nonomura K. (2013). The F-prostaglandin receptor is a novel marker for tumor endothelial cells in renal cell carcinoma. Pathol. Int..

[85] Anderson K.S., Cramer D.W., Sibani S., Wallstrom G., Wong J., Park J., Qiu J., Vitonis A., LaBaer J. (2015). Autoantibody Signature for the Serologic Detection of Ovarian Cancer. J. Proteome Res..

[86] Jiménez-Segovia A., Mota A., Rojo-Sebastián A., Barrocal B., Rynne-Vidal A., García-Bermejo M.-L., Gómez-Bris R., Hawinkels L.J., Sandoval P., Garcia-Escudero R. (2019). Prostaglandin F2α-induced Prostate Transmembrane Protein, Androgen Induced 1 mediates ovarian cancer progression increasing epithelial plasticity. Neoplasia.

[87] Masato M., Miyata Y., Kurata H., Ito H., Mitsunari K., Asai A., Nakamura Y., Araki K., Mukae Y., Matsuda T. (2021). Oral administration of E-type prostanoid (EP) 1 receptor antagonist suppresses carcinogenesis and development of prostate cancer via upregulation of apoptosis in an animal model. Sci. Rep..

[88] Asting A.G., Iresjö B.-M., Nilsberth C., Smedh U., Lundholm K. (2016). Host knockout of E-prostanoid 2 receptors reduces tumor growth and causes major alterations of gene expression in prostaglandin E2-producing tumors. Oncol. Lett..

[89] Heidegger H., Dietlmeier S., Ye Y., Kuhn C., Vattai A., Aberl C., Jeschke U., Mahner S., Kost B. (2017). The Prostaglandin EP3 Receptor Is an Independent Negative Prognostic Factor for Cervical Cancer Patients. Int. J. Mol. Sci..

[90] Czogalla B., Kuhn C., Heublein S., Schmöckel E., Mayr D., Kolben T., Trillsch F., Burges A., Mahner S., Jeschke U. (2019). EP3 receptor is a prognostic factor in TA-MUC1-negative ovarian cancer. J. Cancer Res. Clin. Oncol..

[91] Linares G.D.P., Opperman R., Majumder M., Lala P. (2021). Prostaglandin E2 Receptor 4 (EP4) as a Therapeutic Target to Impede Breast Cancer-Associated Angiogenesis and Lymphangiogenesis. Cancers.

[92] Schotten L.M., Darwiche K., Seweryn M., Yildiz V., Kneuertz P.J., Eberhardt W.E., Eisenmann S., Welter S., Sisson B.E., Pietrzak M. (2021). DNA methylation of PTGER4 in peripheral blood plasma helps to distinguish between lung cancer, benign pulmonary nodules and chronic obstructive pulmonary disease patients. Eur. J. Cancer.

[93] Zhang Y., Huang J., Zou Q., Che J., Yang K., Fan Q., Qian D., Wu J., Bao E., Song L. (2020). Methylated PTGER4 is better than CA125, CEA, Cyfra211 and NSE as a therapeutic response assessment marker in stage IV lung cancer. Oncol. Lett..

[94] Hiken J.F., McDonald J.I., Decker K.F., Sanchez C., Hoog J., VanderKraats N.D., Jung K.L., Akinhanmi M., Rois L.E., Ellis M.J. (2016). Epigenetic activation of the prostaglandin receptor EP4 promotes resistance to endocrine therapy for breast cancer. Oncogene.

[95] Sinha N., Gaston D., Manders D., Goudie M., Matsuoka M., Xie T., Huang W.-Y. (2018). Characterization of genome-wide copy number aberrations in colonic mixed adenoneuroendocrine carcinoma and neuroendocrine carcinoma reveals recurrent amplification of PTGER4 and MYC genes. Hum. Pathol..

[96] Bruno A., Di Francesco L., Coletta I., Mangano G., Alisi M.A., Polenzani L., Milanese C., Anzellotti P., Ricciotti E., Dovizio M. (2010). Effects of AF3442 [N-(9-ethyl-9H-carbazol-3-yl)-2-(trifluoromethyl)benzamide], a novel inhibitor of human microsomal prostaglandin E synthase-1, on prostanoid biosynthesis in human monocytes in vitro. Biochem. Pharmacol..

[97] Powell W.S. (2021). Eicosanoid receptors as therapeutic targets for asthma. Clin. Sci..

[98] Pang J., Qi X., Luo Y., Li X., Shu T., Li B., Song M., Liu Y., Wei D., Chen J. (2021). Multi-omics study of silicosis reveals the potential therapeutic targets PGD2 and TXA2. Theranostics.

[99] Weinstein J.N., Collisson E.A., Mills G.B., Shaw K.R.M., Ozenberger B.A., Ellrott K., Shmulevich I., Sander C., Stuart J.M., The Cancer Genome Atlas Research Network (2013). The Cancer Genome Atlas Pan-Cancer analysis project. Nat. Genet..

[100] Tang Z., Kang B., Li C., Chen T., Zhang Z. (2019). GEPIA2: An enhanced web server for large-scale expression profiling and interactive analysis. Nucleic Acids Res..

[101] Chandrashekar D.S., Bashel B., Balasubramanya S.A.H., Creighton C.J., Ponce-Rodriguez I., Chakravarthi B.V.S.K., Varambally S. (2017). UALCAN: A portal for facilitating tumor subgroup gene expression and survival analyses. Neoplasia.

[102] Nagy Á., Munkácsy G., Győrffy B. (2021). Pancancer survival analysis of cancer hallmark genes. Sci. Rep..

[103] Fekete J.T., Győrffy B. (2019). ROCplot.org: Validating predictive biomarkers of chemotherapy/hormonal therapy/anti-HER2 therapy using transcriptomic data of 3,104 breast cancer patients. Int. J. Cancer.

[104] Fekete J.T., Ősz Á., Pete I., Nagy G.R., Vereczkey I., Győrffy B. (2020). Predictive biomarkers of platinum and taxane resistance using the transcriptomic data of 1816 ovarian cancer patients. Gynecol. Oncol..

[105] Menyhárt O., Fekete J.T., Győrffy B. (2021). Gene expression-based biomarkers designating glioblastomas resistant to multiple treatment strategies. Carcinogenesis.

[106] Gao J., Aksoy B.A., Dogrusoz U., Dresdner G., Gross B.E., Sumer S.O., Sun Y., Jacobsen A., Sinha R., Larsson E. (2013). Integrative Analysis of Complex Cancer Genomics and Clinical Profiles Using the cBioPortal. Sci. Signal..

[107] Cerami E., Gao J., Dogrusoz U., Gross B.E., Sumer S.O., Aksoy B.A., Jacobsen A., Byrne C.J., Heuer M.L., Larsson E. (2012). The cBio cancer genomics portal: An open platform for exploring multidimensional cancer genomics data. Cancer Discov..

[108] Nagy Á., Győrffy B. (2021). muTarget: A platform linking gene expression changes and mutation status in solid tumors. Int. J. Cancer.

[109] Wang J., Duncan D., Shi Z., Zhang B. (2013). WEB-based GEne SeT AnaLysis Toolkit (WebGestalt): Update 2013. Nucleic Acids Res..

[110] Liao Y., Wang J., Jaehnig E.J., Shi Z., Zhang B. (2019). WebGestalt 2019: Gene set analysis toolkit with revamped UIs and APIs. Nucleic Acids Res..

[111] Zhang Q., Liu W., Zhang H.-M., Xie G.-Y., Miao Y.-R., Xia M., Guo A.-Y. (2020). hTFtarget: A Comprehensive Database for Regulations of Human Transcription Factors and Their Targets. Genom. Proteom. Bioinform..

[112] Sondka Z., Bamford S., Cole C.G., Ward S.A., Dunham I., Forbes S.A. (2018). The COSMIC Cancer Gene Census: Describing genetic dysfunction across all human cancers. Nat. Rev. Cancer.

[113] Wu W.-S., Tu B.-W., Chen T.-T., Hou S.-W., Tseng J.T. (2017). CSmiRTar: Condition-Specific microRNA targets database. PLoS ONE.

[114] Sarver A.L., Sarver A.E., Yuan C., Subramanian S. (2018). OMCD: OncomiR Cancer Database. BMC Cancer.

[115] Men C., Chai H., Song X., Li Y., Du H., Ren Q. (2017). Identification of DNA methylation associated gene signatures in endometrial cancer via integrated analysis of DNA methylation and gene expression systematically. J. Gynecol. Oncol..

[116] Shinawi T., Hill V.K., Krex D., Schackert G., Gentle D., Morris M.R., Wei W., Cruickshank G., Maher E.R., Latif F. (2013). DNA methylation profiles of long- and short-term glioblastoma survivors. Epigenetics.

[117] Sjöstedt E., Zhong W., Fagerberg L., Karlsson M., Mitsios N., Adori C., Oksvold P., Edfors F., Limiszewska A., Hikmet F. (2020). An atlas of the protein-coding genes in the human, pig, and mouse brain. Science.

[118] Chen F., Chandrashekar D.S., Varambally S., Creighton C.J. (2019). Pan-cancer molecular subtypes revealed by mass-spectrometry-based proteomic characterization of more than 500 human cancers. Nat. Commun..

[119] Oughtred R., Rust J., Chang C., Breitkreutz B., Stark C., Willems A., Boucher L., Leung G., Kolas N., Zhang F. (2021). The BioGRID database: A comprehensive biomedical resource of curated protein, genetic, and chemical interactions. Protein Sci..

[120] Binder J.X., Pletscher-Frankild S., Tsafou K., Stolte C., O’Donoghue S., Schneider R., Jensen L.J. (2014). Compartments: Unification and visualization of protein subcellular localization evidence. Database.

[121] Szklarczyk D., Gable A.L., Nastou K.C., Lyon D., Kirsch R., Pyysalo S., Doncheva N.T., Legeay M., Fang T., Bork P. (2021). The STRING database in 2021: Customizable protein–protein networks, and functional characterization of user-uploaded gene/measurement sets. Nucleic Acids Res..

[122] Shannon P., Markiel A., Ozier O., Baliga N.S., Wang J.T., Ramage D., Amin N., Schwikowski B., Ideker T. (2003). Cytoscape: A software environment for integrated models of Biomolecular Interaction Networks. Genome Res..

[123] Metsalu T., Vilo J. (2015). ClustVis: A web tool for visualizing clustering of multivariate data using Principal Component Analysis and heatmap. Nucleic Acids Res..

[124] Tsherniak A., Vazquez F., Montgomery P.G., Weir B.A., Kryukov G., Cowley G.S., Gill S., Harrington W.F., Pantel S., Krill-Burger J. (2017). Defining a Cancer Dependency Map. Cell.

[125] Falcetti E., Hall S.M., Phillips P.G., Patel J., Morrell N.W., Haworth S.G., Clapp L.H. (2010). Smooth Muscle Proliferation and Role of the Prostacyclin (IP) Receptor in Idiopathic Pulmonary Arterial Hypertension. Am. J. Respir. Crit. Care Med..

[126] Fetalvero K.M., Shyu M., Nomikos A.P., Chiu Y.-F., Wagner R.J., Powell R.J., Hwa J., Martin K.A. (2006). The prostacyclin receptor induces human vascular smooth muscle cell differentiation via the protein kinase A pathway. Am. J. Physiol. Heart. Circ. Physiol..

[127] Kawada N., Klein H., Decker K. (1992). Eicosanoid-mediated contractility of hepatic stellate cells. Biochem. J..

[128] Kabashima K., Murata T., Tanaka H., Matsuoka T., Sakata D., Yoshida N., Katagiri K., Kinashi T., Tanaka T., Miyasaka M. (2003). Thromboxane A2 modulates interaction of dendritic cells and T cells and regulates acquired immunity. Nat. Immunol..

[129] Moalli F., Cupovic J., Thelen F., Halbherr P., Fukui Y., Narumiya S., Ludewig B., Stein J.V. (2014). Thromboxane A2 acts as tonic immunoregulator by preferential disruption of low-avidity CD4+ T cell–dendritic cell interactions. J. Exp. Med..

[130] Fan Z., Duan J., Wang L., Xiao S., Li L., Yan X., Yao W., Wu L., Zhang S., Zhang Y. (2019). PTK2 promotes cancer stem cell traits in hepatocellular carcinoma by activating Wnt/β-catenin signaling. Cancer Lett..

[131] Wilson S., Filipp F.V. (2018). A network of epigenomic and transcriptional cooperation encompassing an epigenomic master regulator in cancer. NPJ Syst. Biol. Appl..

[132] Slamon D.J., Clark G.M., Wong S.G., Levin W.J., Ullrich A., McGuire W.L. (1987). Human breast cancer: Correlation of relapse and survival with amplification of the HER-2/neu oncogene. Science.

[133] Slamon D.J., Godolphin W., Jones L.A., Holt J.A., Wong S.G., Keith D.E., Levin W.J., Stuart S.G., Udove J., Ullrich A. (1989). Studies of the HER-2/neu proto-oncogene in human breast and ovarian cancer. Science.

[134] Rauluseviciute I., Drabløs F., Rye M.B. (2020). DNA hypermethylation associated with upregulated gene expression in prostate cancer demonstrates the diversity of epigenetic regulation. BMC Med. Genom..

[135] Chen L., Cao Y., Rong D., Wang Y., Cao Y. (2017). MicroRNA-605 functions as a tumor suppressor by targeting INPP4B in melanoma. Oncol. Rep..

[136] Yan X., Liu X., Wang Z., Cheng Q., Ji G., Yang H., Wan L., Ge C., Zeng Q., Huang H. (2018). MicroRNA-486-5p functions as a tumor suppressor of proliferation and cancer stem-like cell properties by targeting Sirt1 in liver cancer. Oncol. Rep..

[137] Qu X., Gao D., Ren Q., Jiang X., Bai J., Sheng L. (2018). miR-211 inhibits proliferation, invasion and migration of cervical cancer via targeting SPARC. Oncol. Lett..

[138] Gupta R., Malvi P., Parajuli K.R., Janostiak R., Bugide S., Cai G., Zhu L.J., Green M.R., Wajapeyee N. (2020). KLF7 promotes pancreatic cancer growth and metastasis by up-regulating ISG expression and maintaining Golgi complex integrity. Proc. Natl. Acad. Sci. USA.

[139] Lai Q., Li Q., He C., Fang Y., Lin S., Cai J., Ding J., Zhong Q., Zhang Y., Wu C. (2020). CTCF promotes colorectal cancer cell proliferation and chemotherapy resistance to 5-FU via the P53-Hedgehog axis. Aging.

[140] Xu X., Wu Y., Yi K., Hu Y., Ding W., Xing C. (2021). IRF1 regulates the progression of colorectal cancer via interferon-induced proteins. Int. J. Mol. Med..

[141] Sava G., Perissin L., Zorzet S., Piccini P., Giraldi T. (1989). Antimetastatic action of the prostacyclin analog Iloprost in the mouse. Clin. Exp. Metastasis.

[142] Menter D.G., Harkins C., Onoda J., Riorden W., Sloane B.F., Taylor J.D., Honn K.V. (1987). Inhibition of tumor cell induced platelet aggregation by prostacyclin and carbacyclin: An ultrastructural study. Invasion Metastasis.

[143] Kalfert D., Ludvikova M., Pesta M., Ludvik J., Dostalova L., Kholová I. (2020). Multifunctional Roles of miR-34a in Cancer: A Review with the Emphasis on Head and Neck Squamous Cell Carcinoma and Thyroid Cancer with Clinical Implications. Diagnostics.

[144] Li Y.-Q., Ren X., He Q.-M., Xu Y.-F., Tang X.-R., Sun Y., Zeng M.-S., Kang T.-B., Liu N., Ma J. (2015). MiR-34c suppresses tumor growth and metastasis in nasopharyngeal carcinoma by targeting MET. Cell Death Dis..

[145] Sun C., Huang C., Li S., Yang C., Xi Y., Wang L., Zhang F., Fu Y., Li D. (2016). Hsa-miR-326 targets CCND1 and inhibits non-small cell lung cancer development. Oncotarget.

[146] Geng R., Zheng Y., Zhou D., Li Q., Li R., Guo X. (2020). ZBTB7A, a potential biomarker for prognosis and immune infiltrates, inhibits progression of endometrial cancer based on bioinformatics analysis and experiments. Cancer Cell Int..

[147] Ershov P.V., Mezentsev Y.V., Kopylov A.T., Yablokov E.O., Svirid A., Lushchyk A.Y., Kaluzhskiy L.A., Gilep A.A., Usanov S.A., Medvedev A.E. (2019). Affinity Isolation and Mass Spectrometry Identification of Prostacyclin Synthase (PTGIS) Subinteractome. Biology.

[148] Skogastierna C., Björkhem-Bergman L., Bergman P., Eliasson E., Rane A., Ekström L. (2013). Influence of Simvastatin on the Thromboxane and Prostacyclin Pathways, In Vitro and In Vivo. J. Cardiovasc. Pharmacol..

[149] Levine L. (2003). Statins stimulate arachidonic acid release and prostaglandin I2 production in rat liver cells. Lipids Health Dis..

[150] Chiefari E., Mirabelli M., La Vignera S., Tanyolaç S., Foti D.P., Aversa A., Brunetti A. (2021). Insulin Resistance and Cancer: In Search for a Causal Link. Int. J. Mol. Sci..

[151] Kobayashi T., Nakatani Y., Tanioka T., Tsujimoto M., Nakajo S., Nakaya K., Murakami M., Kudo I. (2004). Regulation of cytosolic prostaglandin E synthase by phosphorylation. Biochem. J..

[152] Neuschäfer-Rube F., Hermosilla R., Rehwald M., Rönnstrand L., Schülein R., Wernstedt C., Püschel G.P. (2004). Identification of a Ser/Thr cluster in the C-terminal domain of the human prostaglandin receptor EP4 that is essential for agonist-induced beta-arrestin1 recruitment but differs from the apparent principal phosphorylation site. Biochem. J..

[153] Smyth E.M., Li W.H., FitzGerald G.A. (1998). Phosphorylation of the Prostacyclin Receptor during Homologous Desensitization. J. Biol. Chem..

