# Supplementary material for: Prostanoid Signaling in Cancers: Expression and Regulation Patterns of Enzymes and Receptors
Source: Biology (Basel). 2022 Apr 13;11(4):590. doi: 10.3390/biology11040590 (PMC9029281; doi:10.3390/biology11040590)
Supplement: Supplementary file 1 [file biology-11-00590-s001.zip › biology-1668062-supplementary.pdf]

SUPPLEMENTARY MATERIAL

Prostanoid Signaling in Cancers: Expression and Regulation Patterns of Enzymes and Receptors

Pavel V. Ershov *, Evgeniy O. Yablokov, Leonid A. Kaluzhskiy, Yuri V. Mezentsev and Alexis S. Ivanov

Institute of Biomedical Chemistry, 10 Building 8, Pogodinskaya Street, 119121 Moscow, Russia; evgeniy.yablokov@ibmc.msk.ru (E.O.Y.); leonid.kaluzhskiy@ibmc.msk.ru (L.A.K.); yuri.mezentsev@ibmc.msk.ru (Y.V.M.); alexei.ivanov@ibmc.msk.ru (A.S.I.)

* Correspondence: pavel79@inbox.ru; Tel.: +7-499-246-71-15

Table S1. The associations of prostanoid metabolizing enzymes and prostanoid receptors with neoplastic transformation.

	**Prostanoids metabolizing enzymes**	
**TBXAS1**	**Treatment with glycyrrhizin profoundly reduces expression of *TBXAS1* in human lung adenocarcinoma mice xenografts. The anti-cancer effects of glycyrrhizin are possibly attributable to the suppression of the thromboxane A_2 pathway.**	[1]
TBXAS1	NNK, a smoking carcinogen, promotes the expression of lung cancer stem cells-related β-catenin and Nanog. These effects are decreased by ozagrel, a TBXAS1 blocker, implying a role of TBXAS1 in suppression of NNK-induced lung tumorigenesis.	[2]
TBXAS1	TBXAS1 overexpression is associated with higher levels of VEGF, microvessel density, and apoptosis reduction. TBXAS1 promotes tumor growth which can be mediated through tumor angiogenesis.	[3]
TBXAS1, TBXA2R	TBXAS1 and TBXA2R are highly expressed in human breast tumors and premalignant lesions, but not in normal tissues. Thromboxane A_2 pathway is associated with HER2-positive breast cancer and lymph node metastasis.	[4]
PTGIS	*PTGIS* is frequently down-regulated in cancer and PTGIS overexpression suppressed carcinogenesis. Gene polymorphisms of PTGIS' promoter region is associated with cancer risk.	[5]
PTGIS	Prostacyclin can inhibit lung cancer progression and prostacyclin analogs may serve as novel immunomodulatory agents.	[6]
PTGIS	*PTGIS* can be considered as a candidate biomarker for advanced prostate cancer. *PTGIS* dramatically reduced in all the PCa cell lines compared with the normal prostate cell line.	[7]
PTGIS	*PTGIS* expression level in the colorectal cancer hepatic metastases was noticeably higher than in the primary cancer tissue. There is a correlation between higher *PTGIS* expression levels and shorter overall survival.	[8]
PTGIS, PTGDS	*PTGIS, PTGDS, PTGES, AKR1C3* and *TBXAS1* transcripts were elevated in benign prostatic hyperplasia, whereas in PRAD, a decrease in *PTGIS* and *PTGDS* expression is observed.	[9]
PTGDS	*PTGDS* is downregulated in the endometrial cancer compared with the atypical hyperplasia and normal endometrial. *PTGDS* expression is an independent risk factor for worse cancer prognosis.	[10]
PTGDS	*PTGDS* expression is down-regulated in lung cancer and over-expression of *PTGDS* decreases the invasiveness of lung adenocarcinoma cells.	[11]
PTGDS	High levels of *PTGDS* expression correlated with a reduction of tumor proliferation. PGD_2 treatment significantly inhibited the proliferation and migration of breast cancer cells.	[12]

PTGDS	*PTGDS* promoted tumorigenesis through MYH9-mediated regulation of Wnt-β-catenin-STAT3 signaling. PTGDS inhibition may be a novel therapeutic strategy for diffuse large B-cell lymphoma treatment.	[13]
PTGDS	Systems biology analysis showes that *PTGDS* has abnormal expression and methylation in colorectal cancer. Lowly-expressed and highly-methylated *PTGDS* had a worse prognosis.	[14]
PTGDS, PTGDR2	YAP1 inhibited the expression of lipocalin-type *PTGDS* and *PTGDR2* by gain- and loss-of-function experiments. Overexpression of *PTGDS* and *PTGDR2* suppressed the proliferation and reversed the pro-tumor effect of YAP in vivo.	[15]
AKR1C3	AKR1C3 increases androgen receptor (AR) splice variant 7 (AR-V7) expression in enzalutamide-resistant prostate cancer cells. AKR1C3 reprograms AR signaling and targeting AKR1C3 with indomethacin significantly decreases AR/AR-V7 protein expression.	[16]
AKR1C3	AKR1C3 is implicated in the production of androgens in castration-resistant prostate cancer (CRPC), polycystic ovarian syndrome, production of aromatase substrates in breast cancer and non-hormonal dependent malignancies.	[17]
PTGIS	*PTGIS* was differentially expressed and protein level was reduced in oral squamous cell carcinoma. Due to involvement in oxidative stress, PTGIS could modulate cancer development.	[18]
PTGES	PTGES protein stability is connected with complex formation PTGES / USP9X, which prevented it from ubiquitin-dependent proteasome degradation and USP9X expression was highly correlated with PTGES expression in non-small-cell lung carcinoma.	[19]
PTGES	Up-regulation of *PTGES* was associated with reduced overall and progression-free patient survival. Increased PGE2 synthesis via ADRB2–Nf-kB–PTGS2 axis drives tumor growth and metastasis.	[20]
PTGES	PTGES/PGE2 signaling links immunosuppression and metastasis in an inflammatory lung microenvironment of Gprc5a-ko mouse model.	[21]
PTGES	COX2- and mPGES1-overexpressing carcinoma cells were more efficient at forming tumors thus providing evidence for a role of PTGS2 and PTGES in colon cancer progression.	[22]
PTGES	The increase in PD-L1 expression, infiltration of T-cells and/or dendritic cells into tumors, suppression of tumor growth and response to PD-1 checkpoint inhibitors were observed in PTGES-knockout melanoma mouse model	[23]
PTGES2	miR-155 has dual-regulatory mode to reprogram the PGE_2/PGD_2 balance by up-regulating PTGES/PTGES2 and down-regulating PTGDS in breast cancer patients.	[24]
PTGES2	miR-146a, a modulator of IL-17 responses, suppresses colorectal cancer by targeting PTGES2.	[25]
PTGES2	Increased expression of *PTGES2* associates with age (P=0.0092) and the depth of myometrial invasion (P<0.0001).	[26]
PTGES3	*PTGES3* was identified as a hub gene which is closely related to cholangiocarcinoma development, progression and prognosis.	[27]
PTGES3	*PTGES3* expression was identified as a promising prognostic biomarker for primary breast cancer patients.	[28]
CBR1	Genetic inhibition of *CBR1* suppressed pancreatic cancer cell proliferation by regulating ROS generation. Gemcitabine induced *CBR1* gene expression, which could limit the anti-tumor activity of gemcitabine.	[29]
CBR1	Cell proliferation significantly decreased in *CBR1*-overexpressed cells, the number of metastatic foci was significantly higher in mice injected with *CBR1* siRNA compared to wild-type tumors.	[30]

CBR1	CBR1 plays an important role in metastasis of head and neck squamous cell carcinoma via regulation of ROS-mediated β-catenin activity. CBR1 may be a marker for metastasis progression.	[31]
CBR1	*CBR1* expression was correlated with the N classification (P<0.0001), stage (P=0.0018) and outcome (P=0.0095) as well as overall survival. Suppression of *CBR1* by *CBR1*-siRNA increased cancer cell proliferation and migration.	[32]
CBR3	CBR3-AS1 expression was significantly increased in breast cancer tissues and was closely correlated with poor prognosis. CBR3-AS1 overexpression promoted adriamycin resistance in breast cancer cells.	[33]
CBR3	CBR3-AS1 functions as an oncogene through the CBR3-AS1/miR-409-3p/SOD1 pathway, and may represent a new therapeutic target in non-small-cell lung carcinoma.	[34]
CBR3	*CBR3* 730G>A (*rs1056892*, HR = 2.57, 1.07-6.18) is a new pharmacogenomic biomarker that can be used to predict tumor recurrence in non-muscle-invasive bladder cancer patients receiving intravesical instillations pirarubicin.	[35]
	**Prostanoids receptors**	
PTGDR1, PTGIR, TBXA2R	*PTGDR1* methylation was most highly correlated with recurrence in patients with hypopharyngeal cancer. A similar correlation was observed for *PTGER4* in patients with laryngeal cancer. Methylation of the *PTGIR* and *TBXA2R* promoters was positively correlated with recurrence in oropharyngeal cancer.	[36]
PTGDR1, PTGDR2, PTGIR	*PTGDR1*, *PTGDR2* and *PTGIR* methylation status biomarkers for the clinical management of HPV-associated oropharyngeal cancers.	[37]
TBXA2R	TXA_2/*TBXA2R* signalling act as a neoplastic- and epigenetic-regulator, promoting chromatin remodelling and androgen receptor-mediated transcriptional activation in castration-resistant prostate cancer.	[38]
TBXA2R	Polymorphic variant *TBXA2R* (rs200445019) correlates with cancer metastasis across several cancer types. A potent and selective antagonist of TXA_2 blocks metastasis formation.	[39]
TBXA2R	*TBXA2R* enhances triple negative breast cancer (TNBC) cell migration, invasion and protects TNBC cells from DNA damage thus providing novel treatment opportunities.	[40]
TBXA2R	*TBXA2Rα* and *TBXA2Rβ* isoform expression pattern is differentially regulated via CpG methylation in the benign and the prostate cancer tissue Expression of isoforms have diagnostic significance as a predictor of prostate cancer recurrence.	[41]
PTGDR1, PTGDR2	High expression of *PTGDR2* in tumor tissue negatively correlates with overall survival. TNM stage and metastasis positively correlated with *PTGDR2* expression.	[42]
PTGDR2	*PTGDR2* is statistically associated with patient overall survival or disease-free survival in colorectal cancer.	[43]
PTGFR	$PGF_{2\alpha}$-PTGFR promotes cell proliferation; increases the abundance of cyclins, cyclin-dependent kinases, PCNA and decreases p21.	[44]
PTGFR	*PTGFR* transcripts significantly increase at the critical transition from stage T2 to T3/T4 and remain elevated in advanced prostate cancer stages.	[45]
PTGFR	The expression levels of *PTGFR* are higher in tumor than in normal endothelial cells. PTGFR protein is expressed in human tumor blood vessels.	[46]
PTGFR	Sera from ovarian cancer (OV) patients containing auto-antibodies (AAb) to PTGFR had AUC > 60% (p < 0.01). PTGFR is a potential biomarker for the early detection of OV.	[47]
PTGFR	High *PTGFR* transcript levels are associated with lower survival of OV patients.	[48]

PTGER1	PTGER1 antagonist delayed carcinogenesis and progression of prostate cancer (PC) in knock-in mouse model. Inhibition of PTGER1 pathway may be a novel chemopreventive strategy for PC.	[49]
PTGER2	Tumor growth was reduced in *PTGER2*-knockout tumor-bearing mice. PGE_2 signaling via PTGER2 affected genes involved in tumor progression.	[50]
PTGER3	High expression of *PTGER3* in cervical cancer patients is correlated with poor prognosis.	[51]
PTGER3	*PTGER3* expression is significantly higher in clear-cell ovarian carcinoma ($p < 0.001$) compared to the other histological subtypes. *PTGER3* expression associates with mucin-1 negative OV patients overall survival.	[52]
PTGER4	PTGER4 activity promotes breast cancer cell migration, invasion, angiogenesis, lymphangiogenesis and PTGER4 antagonists may be as promising anticancer drugs.	[53]
PTGER4	*PTGER4* hypermethylation was more frequently observed in plasma samples of patients with lung cancer than in controls ($p = 0.0004$).	[54]
PTGER4	Blood levels of methylated *PTGER4* predict overall survival rate of patients with stage IV lung cancer as well as therapeutic efficacy.	[55]
PTGER4	*PTGER4* is upregulated after demethylation in aromatase inhibitor resistant cells and is essential for estrogen-independent growth. PTGER4 promotes resistance via ligand-independent activation of the ERα-cofactor CARM1.	[56]
PTGER4	*PTGER4* gene amplification frequency in mixed adenoneuroendocrine carcinoma is higher than in adenocarcinoma (10.5% vs 0.3%).	[57]

Table S2. Cell lines dependency on target genes' knockouts and knockdowns.

Gene name	Cancer	Cell line	Gene effect score	Gene expression log2(TPM+1)
TBXAS1	**gallbladder adenocarcinoma**	**OCUG1**	**-0.97**	**6.09**
PTGES	brain cancer	PFSK1	-0.58	3.34
	colorectal cancer	SW948	-0.51	3.17
PTGES2	sarcoma cancer	SC S214	-0.79	6.14
	head and neck cancer	SNU46	-0.67	7.3
PTGES3	**brain cancer**	**ONS76**	**-0.98**	**7.28**
	colorectal cancer	CCK81	-0.71	8.54
	gastric cancer	SCH	-0.7	no data
	kidney cancer	UMRC7	-0.69	5.57
	liver cancer	HUH7	-0.73	8.31
	lung cancer	CHAGOK1	-0.76	7.33
	lymphoma	KIJK	-0.69	7.89
	ovarian cancer	SKOV3	-0.68	8.62
	pancreatic cancer	PACADD137	-0.65	8.92
	neuroblastoma	NB69	-0.57	no data
	skin cancer	COLO679	-0.74	8.04
	thyroid cancer	SW579	-0.69	7.62
CBR3	liposarcoma	95T1000	-0.62	4.29
TBXA2R	breast cancer	SUM52PE	-0.55	0.45
	colorectal cancer	SW948	-0.58	0.06
PTGDR	**lung cancer**	**CORL279**	**-1.04**	**0.03**
PTGER3	leukemia	HEL9217	-0.76	5.24
	cervical cancer	SW954	-0.56	0
	esophageal cancer	KYSE150	-0.52	0.01
	kidney cancer	VMRCRCW	-0.72	0.01
	lymphoma	C8166	-0.56	3.38
	head and neck cancer	H413	-0.58	0
	uterine cancer	JHUEM7	-0.61	0.01
PTGER4	lung cancer	CORL279	-0.79	0.32
	**lymphoma**	**C8166**	**-1.37**	**5.1**

Table S3. Over-representation analysis of genes showing similar expression patterns with target genes involved in prostanoid signaling.

Target genes (cluster)	Tumor	Co-expressed genes	Enrichment with Reactome pathway terms
TBXAS1 (I)	KIRC	71	Fc-gamma receptor (FCGR) dependent phagocytosis<br>Classical antibody-mediated complement activation<br>Toll-like receptor cascades
	LIHC	73	Classical antibody-mediated complement activation<br>Immunoregulatory interactions between a lymphoid and a non-lymphoid cell
	GBM	138	Cross-presentation of particulate exogenous antigens (phagosomes)<br>Classical antibody-mediated complement activation<br>MyD88 deficiency (TLR2/4)
	PCPG	139	Initial triggering of complement<br>Fc-gamma receptor (FCGR) dependent phagocytosis
	OV	134	Fc-gamma receptor (FCGR) dependent phagocytosis<br>Immunoregulatory interactions between a lymphoid and a non-lymphoid cell<br>MyD88 deficiency (TLR2/4)
	ACC	149	Cross-presentation of particulate exogenous antigens (phagosomes)<br>Fcgamma receptor (FCGR) dependent phagocytosis<br>Regulation of complement cascade
	LGG	191	Cross-presentation of particulate exogenous antigens (phagosomes)<br>Classical antibody-mediated complement activation<br>Antigen activates B cell receptor (BCR) leading to generation of second messengers<br>DAP12 signaling<br>FCERI mediated MAPK activation
	UVM	229	Classical antibody-mediated complement activation<br>Endosomal/vacuolar pathway<br>Translocation of ZAP-70 to immunological synapse<br>RHO GTPases activate NADPH oxidases<br>Antigen processing-cross presentation
PTGIS (I)	BLCA	54	Smooth muscle contraction<br>RHO GTPases activate CIT
	CHOL	63	Extracellular matrix organization
	COAD	71	Molecules associated with elastic fibres
	READ	70	Muscle contraction
	PRAD	157	Smooth muscle contraction<br>Cell-extracellular matrix interactions
	TGCT	391	Extracellular matrix organization<br>Collagen chain trimerization<br>MET activates PTK2 signaling<br>Elastic fibre formation
PTGDS (I)	ESCA	115	Phosphorylation of CD3 and TCR zeta chains<br>Signaling by Rho GTPases<br>Immune system
	CHOL	320	Antigen activates B cell receptor (BCR) leading to generation of second messengers<br>Fc-gamma receptor (FCGR) dependent phagocytosis<br>DAP12 signaling<br>Interleukin receptor SHC signaling

	KICH	374	PD-1 signaling<br>Extracellular matrix organization
PTGES3 (I)	THCA	183	Processing of capped intron-containing pre-mRNA<br>mRNA splicing - major pathway
	SKCM	432	rRNA modification in the nucleus and cytosol<br>mRNA splicing - major pathway<br>Nucleotide excision repair
PTGES (II)	ACC	111	Extracellular matrix organization<br>Collagen degradation<br>Collagen formation
TBXA2R (III)	COAD	274	Defective B3GALTL causes Peters-plus syndrome (PpS)<br>Regulation of insulin-like growth factor (IGF) transport and uptake by insulin-like growth factor binding proteins (IGFBPs)<br>Rho GTPase cycle
PTGFR (III)	COAD	148	Collagen formation (degradation)<br>Extracellular matrix organization<br>Cell surface interactions at the vascular wall
	TGCT	69	Extracellular matrix organization<br>ECM proteoglycans<br>Elastic fibre formation
PTGER3 (III)	COAD	118	Collagen chain trimerization<br>Molecules associated with elastic fibres
	LIHC	26	Collagen formation (degradation)<br>NCAM1 interactions
	PAAD	30	Molecules associated with elastic fibres
	READ	50	Elastic fibre formation
	TGCT	85	Smooth muscle contraction<br>ECM proteoglycans<br>Platelet activation, signaling and aggregation<br>RHO GTPases activate CIT<br>RHO GTPases Activate ROCKs
PTGER4 (III)	ACC	134	Collagen formation (degradation)<br>Signaling by Interleukins<br>Immunoregulatory interactions between a lymphoid and a non-lymphoid cell<br>Binding and uptake of ligands by scavenger receptors<br>Innate immune system
	GBM	26	Integrin cell surface interactions<br>Assembly of collagen fibrils and other multimeric structures<br>MET activates PTK2 signaling<br>Anchoring fibril formation
	KIRP	63	Innate immune system<br>Trafficking and processing of endosomal TLR
	MESO	50	Generation of second messenger molecules<br>Immune system<br>Translocation of ZAP-70 to immunological synapse

Table S4. Concordance between changes of gene expression and promoter methylation patterns.

Gene	Tumor	Differential gene expression	Differential gene promoter methylation*
Down-regulated genes and an increase in promoter methylation in tumors			
PTGDR	COAD	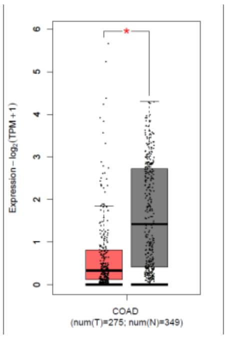	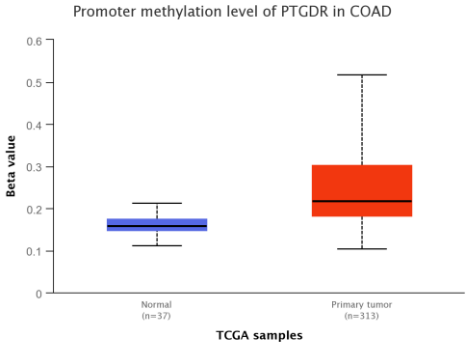
PTGDR	UCES	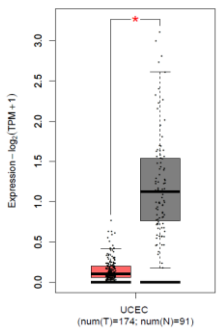	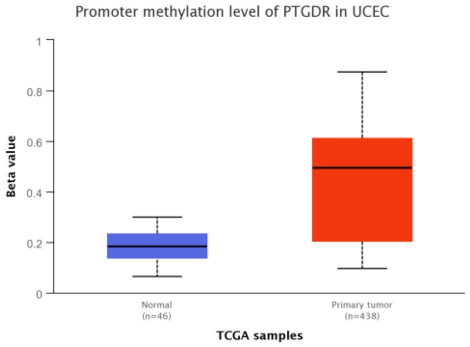
PTGER3	COAD	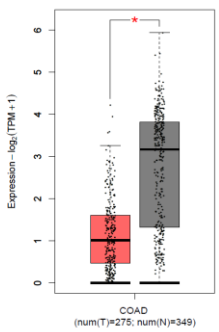	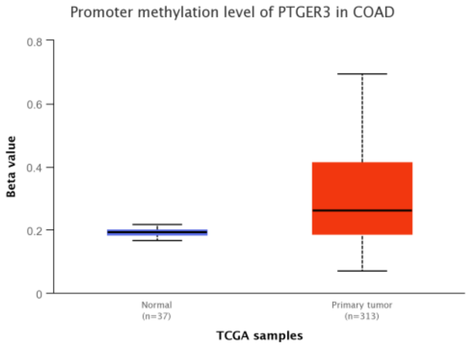
PTGIR	KIRP	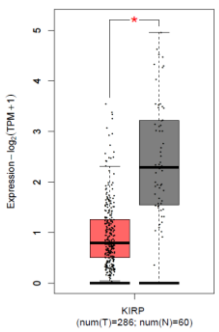	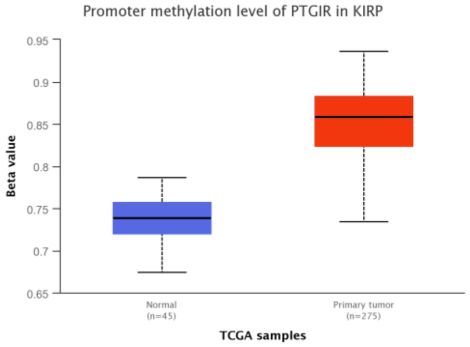

PTGIR	UCES	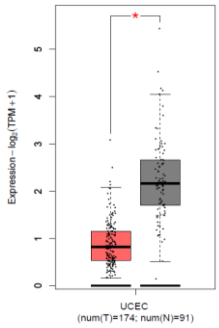	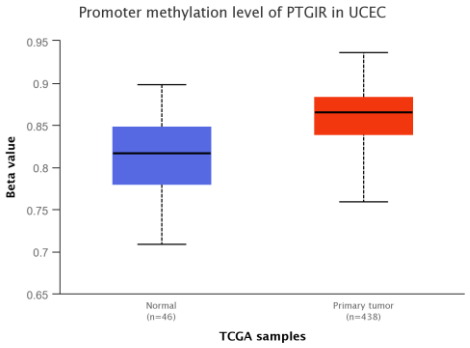
TBXA2R	LUAD	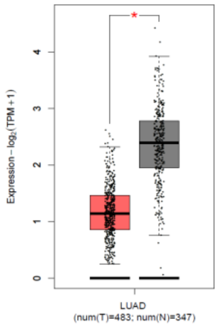	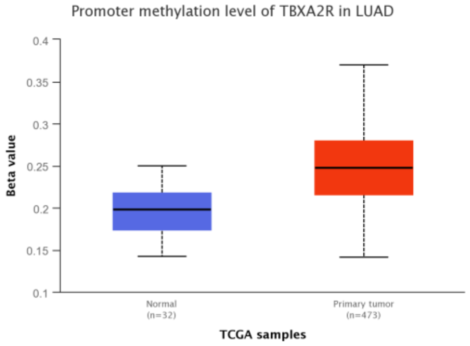
TBXA2R	LUSC	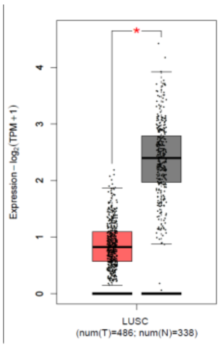	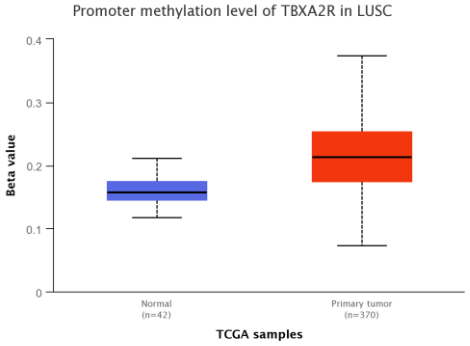

Up-regulated genes and a decrease in promoter methylation in tumors

CBR3	LUSC	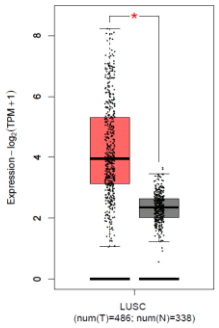	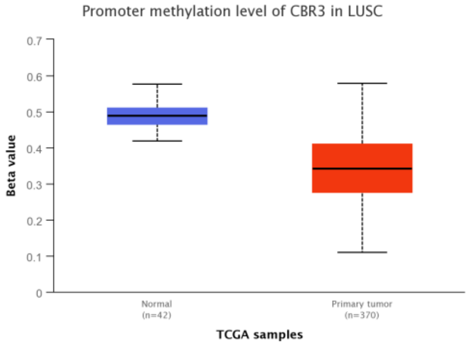
AKR1C3	LUSC	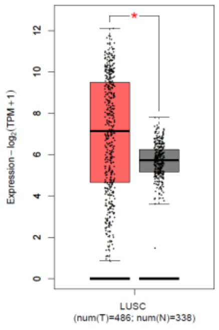	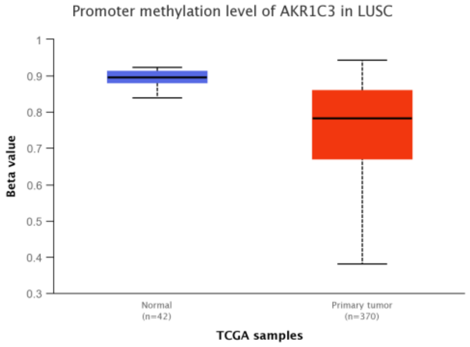

Down-regulated genes and a decrease in promoter methylation in tumors

PTGDS	ESCA	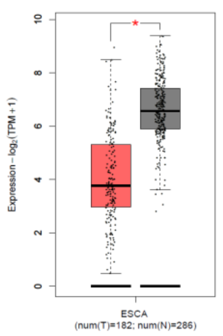	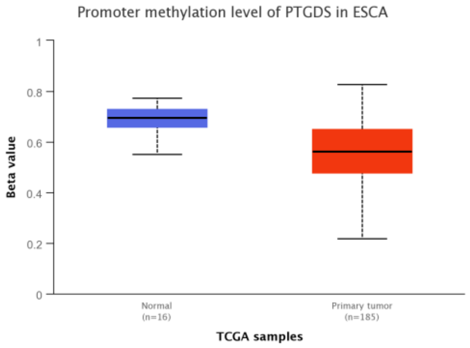
PTGDS	HNSC	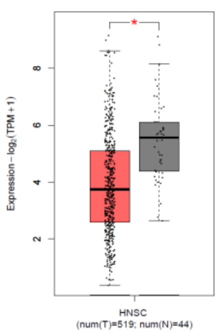	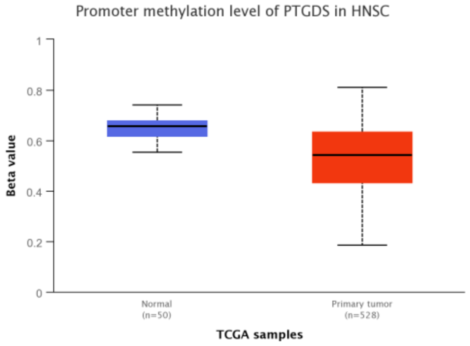

*p-value < 0.05.

Table S5. Over-representation analysis of master regulators using KEGG database as a data source.

Description	Size	Overlap	P-value	Genes
Pathways in cancer	524	6	2.22e-5	*AR; EP300; MAX; RELA; SP1; SPI1*
Transcriptional misregulation in cancer	186	4	7.71e-5	*MAX; RELA; SP1; SPI1*
Prostate cancer	97	3	2.58e-4	*AR; EP300; RELA*
Maturity onset diabetes of the young	26	2	5.43e-4	*FOXA2; HNF4A*
Herpes simplex infection	185	3	0.0017	*EP300; POLR2A; RELA*
Huntington disease	193	3	0.0019	*EP300; POLR2A; SP1*
Mitophagy	65	2	0.0033	*RELA; SP1*
Acute myeloid leukemia	66	2	0.0034	*RELA; SPI1*
Human T-cell leukemia virus 1 infection	255	3	0.0042	*EP300; RELA; SPI1*
TGF-beta signaling pathway	84	2	0.0055	*EP300; SP1*

Table S6. A list of predicted regulatory oncomiRs for target genes involved in prostanoid signalling.

miR name	Score	Gene name
hsa-miR-34b-3p	0.461	*TBXAS1*
hsa-miR-502-5p	0.484	*TBXAS1*
hsa-miR-496	0.539	*PTGIS*
hsa-miR-34a-5p	0.547	*PTGIS*
hsa-miR-449a	0.542	*PTGIS*
hsa-miR-449b-5p	0.542	*PTGIS*
hsa-miR-34c-5p	0.541	*PTGIS*
hsa-miR-888-3p	0.763	*PTGES3*
hsa-let-7a-2-3p	0.706	*PTGES3*
hsa-let-7g-3p	0.655	*PTGES3*
hsa-miR-422a	0.586	*PTGES3*
hsa-miR-378a-3p	0.568	*PTGES3*
hsa-miR-223-5p	0.549	*PTGES3*
hsa-miR-510-5p	0.532	*PTGES3*
hsa-miR-377-3p	0.522	*PTGES3*
hsa-miR-522-3p	0.506	*PTGES3*
hsa-miR-224-3p	0.5	*PTGES3*
hsa-miR-495-3p	0.498	*PTGES3*
hsa-miR-19a-3p	0.491	*PTGES3*
hsa-miR-421	0.482	*PTGES3*
hsa-miR-136-5p	0.474	*PTGES3*
hsa-miR-376c-3p	0.454	*PTGES3*
hsa-miR-338-5p	0.444	*PTGES3*
hsa-miR-605-5p	0.414	*PTGES3*
hsa-miR-519d-3p	0.414	*PTGES3*
hsa-miR-767-3p	0.78	*PRXL2B*
hsa-miR-486-3p	0.52	*PRXL2B*
hsa-miR-211-3p	0.485	*PRXL2B*
hsa-miR-423-5p	0.463	*PRXL2B*
hsa-miR-149-3p	0.411	*PRXL2B*
hsa-miR-149-3p	0.516	*PTGES*
hsa-miR-377-3p	0.474	*PTGES*
hsa-miR-508-5p	0.458	*PTGES*
hsa-miR-296-5p	0.453	*PTGES*
hsa-miR-329-3p	0.424	*PTGES*
hsa-miR-362-3p	0.42	*PTGES*
hsa-miR-335-3p	0.757	*CBR1*
hsa-miR-511-5p	0.563	*AKR1C3*
hsa-miR-184	0.562	*AKR1C3*
hsa-miR-149-5p	0.641	*PTGIR*
hsa-miR-589-5p	0.488	*PTGIR*
hsa-miR-326	0.483	*PTGIR*
hsa-miR-608	0.402	*PTGIR*
hsa-miR-135a-5p	0.641	*PTGER1*
hsa-miR-135b-5p	0.554	*PTGER1*
hsa-miR-31-5p	0.855	*TBXA2R*

hsa-miR-182-5p	0.732	*TBXA2R*
hsa-miR-520a-5p	0.553	*TBXA2R*
hsa-miR-149-3p	0.539	*TBXA2R*
hsa-miR-525-5p	0.537	*TBXA2R*
hsa-miR-485-5p	0.509	*TBXA2R*
hsa-miR-425-5p	0.5	*TBXA2R*
hsa-miR-340-3p	0.467	*TBXA2R*
hsa-miR-657	0.447	*TBXA2R*
hsa-miR-30b-3p	0.433	*TBXA2R*
hsa-miR-508-5p	0.41	*TBXA2R*
hsa-miR-185-5p	0.766	*PTGDR2*
hsa-miR-519e-5p	0.708	*PTGDR2*
hsa-miR-625-5p	0.583	*PTGDR2*
hsa-miR-515-5p	0.581	*PTGDR2*
hsa-miR-661	0.523	*PTGDR2*
hsa-miR-550a-5p	0.497	*PTGDR2*
hsa-miR-499a-5p	0.629	*PTGDR*
hsa-miR-200a-3p	0.564	*PTGDR*
hsa-miR-27a-3p	0.559	*PTGDR*
hsa-miR-27b-3p	0.554	*PTGDR*
hsa-miR-141-3p	0.54	*PTGDR*
hsa-miR-20a-5p	0.539	*PTGDR*
hsa-miR-106b-5p	0.538	*PTGDR*
hsa-miR-140-3p	0.526	*PTGDR*
hsa-miR-511-5p	0.524	*PTGDR*
hsa-miR-362-3p	0.522	*PTGDR*
hsa-miR-106a-5p	0.512	*PTGDR*
hsa-miR-338-5p	0.488	*PTGDR*
hsa-miR-329-3p	0.488	*PTGDR*
hsa-miR-487a-3p	0.435	*PTGDR*
hsa-miR-513a-3p	0.43	*PTGDR*
hsa-miR-130b-3p	0.677	*PTGER2*
hsa-miR-130a-3p	0.629	*PTGER2*
hsa-miR-361-3p	0.598	*PTGER2*
hsa-miR-497-3p	0.566	*PTGER2*
hsa-miR-301a-3p	0.56	*PTGER2*
hsa-miR-454-3p	0.552	*PTGER2*
hsa-miR-515-5p	0.544	*PTGER2*
hsa-miR-19a-3p	0.509	*PTGER2*
hsa-miR-19b-3p	0.509	*PTGER2*
hsa-miR-141-5p	0.471	*PTGER2*
hsa-miR-532-3p	0.471	*PTGER2*
hsa-miR-421	0.439	*PTGER2*
hsa-miR-205-3p	0.436	*PTGER2*
hsa-miR-410-3p	0.436	*PTGER2*
hsa-miR-188-3p	0.433	*PTGER2*
hsa-miR-148b-3p	0.424	*PTGER2*
hsa-miR-452-5p	0.406	*PTGER2*
hsa-let-7f-2-3p	0.821	*PTGFR*
hsa-miR-330-3p	0.669	*PTGFR*
hsa-miR-423-5p	0.624	*PTGFR*

hsa-miR-513a-3p	0.567	*PTGFR*
hsa-miR-590-3p	0.511	*PTGFR*
hsa-miR-145-5p	0.466	*PTGFR*
hsa-miR-150-5p	0.458	*PTGFR*
hsa-miR-520f-3p	0.443	*PTGFR*
hsa-miR-34c-3p	0.435	*PTGFR*
hsa-miR-621	0.405	*PTGFR*
hsa-miR-96-5p	0.714	*PTGER3*
hsa-miR-142-5p	0.622	*PTGER3*
hsa-miR-27b-3p	0.566	*PTGER3*
hsa-miR-27a-3p	0.536	*PTGER3*
hsa-miR-380-3p	0.523	*PTGER3*
hsa-miR-151a-3p	0.49	*PTGER3*
hsa-miR-106a-5p	0.454	*PTGER3*
hsa-miR-106b-5p	0.444	*PTGER3*
hsa-miR-590-3p	0.443	*PTGER3*
hsa-miR-152-3p	0.435	*PTGER3*
hsa-miR-561-3p	0.428	*PTGER3*
hsa-miR-148a-3p	0.424	*PTGER3*
hsa-miR-28-5p	0.422	*PTGER3*
hsa-miR-181b-5p	0.421	*PTGER3*
hsa-miR-148b-3p	0.416	*PTGER3*
hsa-miR-20a-5p	0.413	*PTGER3*
hsa-miR-181d-5p	0.407	*PTGER3*
hsa-miR-708-5p	0.402	*PTGER3*
hsa-miR-484	0.804	*PTGER4*
hsa-miR-590-3p	0.657	*PTGER4*
hsa-miR-515-5p	0.634	*PTGER4*
hsa-miR-92a-3p	0.632	*PTGER4*
hsa-miR-32-5p	0.632	*PTGER4*
hsa-miR-92b-3p	0.632	*PTGER4*
hsa-miR-367-3p	0.605	*PTGER4*
hsa-miR-130a-5p	0.601	*PTGER4*
hsa-miR-25-3p	0.593	*PTGER4*
hsa-miR-1226-3p	0.592	*PTGER4*
hsa-miR-24-3p	0.586	*PTGER4*
hsa-miR-372-3p	0.557	*PTGER4*
hsa-miR-520c-3p	0.546	*PTGER4*
hsa-miR-520b	0.543	*PTGER4*
hsa-miR-520e	0.543	*PTGER4*
hsa-miR-34c-3p	0.541	*PTGER4*
hsa-miR-23a-3p	0.531	*PTGER4*
hsa-miR-23b-3p	0.531	*PTGER4*
hsa-miR-302c-3p	0.518	*PTGER4*
hsa-miR-302d-3p	0.518	*PTGER4*
hsa-miR-363-3p	0.516	*PTGER4*
hsa-miR-302a-3p	0.515	*PTGER4*
hsa-miR-302b-3p	0.515	*PTGER4*
hsa-miR-373-3p	0.482	*PTGER4*
hsa-miR-129-2-3p	0.452	*PTGER4*
hsa-miR-520a-3p	0.438	*PTGER4*
hsa-miR-20b-5p	0.435	*PTGER4*

hsa-miR-20a-5p	0.426	*PTGER4*
hsa-miR-520d-3p	0.425	*PTGER4*
hsa-miR-17-5p	0.423	*PTGER4*
hsa-miR-93-5p	0.407	*PTGER4*
hsa-miR-106b-5p	0.401	*PTGER4*

Table S7. Expression ratio (tumor/normal) of predicted master oncomiRs that can regulate mRNAs of genes involved in prostanoid signaling.

Tumors	BRCA	COAD	ESCA	KIRP	LIHC	LUAD	PAAD	PRAD	SKCM	STAD	UCEC
oncomiRs*											
hsa-miR-148b-3p	2.23										3.37
hsa-miR-149-3p		0.05									
hsa-miR-19a-3p						3.71	0.34	2.68		3.97	3.51
hsa-miR-20a-5p			2.71			3.16		4.2		4.24	3.38
hsa-miR-27a-3p		3.48	2.36								2.54
hsa-miR-27b-3p											
hsa-miR-338-5p				0.36							
hsa-miR-34c-3p										2.63	
hsa-miR-377-3p	0.48	0.39									
hsa-miR-421	2.03									3.5	5.16
hsa-miR-423-5p						0.43		2.49			
hsa-miR-508-5p											0.13
hsa-miR-511-5p											
hsa-miR-513a-3p											
hsa-miR-515-5p											
hsa-miR-590-3p	2.14					3.3	0.39				2.82

*molecular targets of human oncomiRs were predicted using CSmiRTar web-based tool (http://cosbi4.ee.ncku.edu.tw/CSmiRTar/) and average normalized score (ANS) above 0.4 was considered significant: miR-106a-5p (PTGDR 0.512, PTGER3 0.454); miR-106b-5p (PTGDR 0.538, PTGER3 0.444, PTGER4 0.401); miR-148b-3p (PTGER2 0.424, PTGER3 0.416); miR-149-3p (PRXL2B 0.411, PTGES 0.516, TBXA2R 0.539); miR-19a-3p (PTGES3 0.491, PTGER2 0.509); miR-20a-5p (PTGDR 0.539, PTGER3 0.413, PTGER4 0.426); miR-27a-3p (PTGDR 0.559, PTGER3 0.536); miR-27b-3p (PTGDR 0.554, PTGER3 0.506); miR-338-5p (PTGES3 0.444, PTGDR 0.488); miR-34c-3p (PTGFR 0.435, PTGER4 0.541); miR-377-3p (PTGES3 0.522, PTGES 0.474); miR-421 (PTGES3 0.482, PTGER2 0.439); miR-423-5p (PRXL2B 0.463, PTGFR 0.624); miR-508-5p (PTGES 0.458, TBXA2R 0.41); miR-511-5p (AKR1C3, 0.563, PTGDR 0.524); miR-513a-3p (PTGDR 0.43, PTGFR 0.567); miR-515-5p (PTGDR2 0.581, PTGER2 0.544, PTGER4 0.634); miR-590-3p (PTGFR 0.511, PTGER3 0.443, PTGER4 0.657).

Table S8. Antibody staining of proteins involved in prostanoid signaling according to the Human Proteome Atlas portal.

Legend

(dark blue)	High antibody staning
(medium blue)	Medium antibody staining
(light blue)	Low antibody staining
(white)	Not detected
(grey)	No data

Protein	Colorectal cancer	Pancreatic cancer	Prostate cancer	Stomach cancer	Lymphoma	Ovarian cancer	Endometrial cancer	Melanoma	Glioma	Thyroid cancer	Lung cancer	Head and neck cancer	Liver cancer	Carcinoid	Renal cancer	Urothelial cancer	Testis cancer	Breast cancer	Cervical cancer	Skin cancer	Antibody
TBXAS1	Medium	Low	Not detected	Not detected	Not detected	Not detected	Not detected	Not detected	Not detected	Not detected	Not detected	Not detected	Not detected	Not detected	Not detected	Not detected	Not detected	Not detected	Not detected	Not detected	HPA031257
PTGIS	Medium	Medium	Medium	Medium	Not detected	Low	Not detected	Medium	Medium	Medium	Low	Low	Medium	Not detected	Medium	Medium	Medium	Medium	Low	Not detected	CAB009517
PTGDS	Medium	Medium	Low	Low	Low	Low	Medium	Low	Low	Low	Medium	Medium	Low	Medium	Medium	Medium	Medium	Medium	Medium	Low	CAB009916
PTGES	Not detected	Not detected	Not detected	Not detected	Not detected	Not detected	Not detected	Not detected	Not detected	Not detected	Not detected	Not detected	Not detected	Not detected	Not detected	Not detected	Not detected	Not detected	Not detected	Not detected	HPA045064
PTGES2	High	Medium	Medium	Medium	Medium	Low	Medium	Medium	Not detected	Low	Medium	Medium	High	Medium	Medium	Medium	Medium	Medium	Low	Low	HPA020733
PTGES3	Medium	Medium	Medium	Low	Low	Low	Low	Low	Low	Not detected	Not detected	Medium	Medium	Medium	Low	Medium	Medium	Medium	Low	Not detected	HPA038672
PRXL2B	Medium	Medium	Medium	Medium	Medium	Medium	Medium	Medium	Medium	Medium	Low	Medium	High	Medium	Medium	Medium	Medium	Medium	Medium	Low	HPA006403
AKR1C3	High	High	Not detected	High	Not detected	Not detected	Not detected	Not detected	Medium	Not detected	Not detected	Not detected	High	Not detected	High	Medium	Not detected	Not detected	Not detected	Not detected	CAB010874
CBR1	Not detected	Not detected	High	Low	Medium	Low	Not detected	Medium	High	High	Low	Not detected	High	High	High	High	Not detected	Not detected	Not detected	Medium	CAB034291
CBR3	Medium	Medium	Medium	Not detected	Medium	Medium	Medium	High	Medium	High	Low	High	Medium	Medium	Medium	Medium	Medium	Medium	Medium	Medium	HPA018434
TBXA2R	No data	No data	No data	No data	No data	No data	No data	No data	No data	No data	No data	No data	No data	No data	No data	No data	No data	No data	No data	No data	no
PTGIR	No data	No data	No data	No data	No data	No data	No data	No data	No data	No data	No data	No data	No data	No data	No data	No data	No data	No data	No data	No data	no
PTGDR	Not detected	Not detected	Not detected	Not detected	Not detected	Not detected	Not detected	Not detected	Not detected	Not detected	Not detected	Not detected	Not detected	Not detected	Not detected	Not detected	Not detected	Not detected	Not detected	Not detected	HPA049668
PTGER1	No data	No data	No data	No data	No data	No data	No data	No data	No data	No data	No data	No data	No data	No data	No data	No data	No data	No data	No data	No data	no
PTGFR	No data	No data	No data	No data	No data	No data	No data	No data	No data	No data	No data	No data	No data	No data	No data	No data	No data	No data	No data	No data	no
PTGDR2	Not detected	Not detected	Not detected	Not detected	Not detected	Not detected	Not detected	Not detected	Not detected	Not detected	Not detected	Not detected	Not detected	Not detected	Not detected	Not detected	Not detected	Not detected	Not detected	Not detected	HPA014259
PTGER2	No data	No data	No data	No data	No data	No data	No data	No data	No data	No data	No data	No data	No data	No data	No data	No data	No data	No data	No data	No data	no
PTGER3	Low	Not detected	Not detected	Not detected	Not detected	Not detected	Medium	Not detected	Not detected	Not detected	Low	Not detected	Not detected	Not detected	Not detected	Low	Medium	Low	High	Not detected	HPA010689
PTGER4	Medium	Medium	Medium	Medium	Not detected	Medium	Medium	Medium	High	Medium	Medium	High	Low	Medium	Medium	Medium	Medium	Medium	Medium	Medium	HPA012756

Table S9. Prediction of genes, which expression patterns can be associated with genetic polymorphism in target genes in melanoma tumors.

Gene Expression	Mean mutant (*n*=45)	Mean wild (*n*=420)	FC (mutant/wild)	Direction	*p*-value	Group #
			Group #1 (TBXAS1, PTGIS, AKR1C3)			
NYNRIN	295.62	600.46	2.04	down	6.40e-04	1
SVEP1	120.02	244.67	2.04	down	5.30e-03	1
RAPGEF4	156.36	319.1	2.04	down	6.80e-03	1
STUM	105.47	214.06	2.04	down	1.77e-02	1
MAOB	318.89	650.08	2.04	down	2.64e-02	1
H2AFY2	104.91	217.34	2.08	down	2.45e-02	1
FAM19A5	248.42	529.88	2.13	down	1.37e-02	1
SPON1	271.09	585.87	2.17	down	2.53e-02	1
PP7080	234.67	522.83	2.22	down	1.75e-02	1
CNTN4	110.93	251.88	2.27	down	6.62e-03	1
SORL1	740.89	1739.45	2.33	down	9.71e-04	1
ELN	256.58	590.5	2.33	down	4.57e-03	1
LPAR1	185.09	428.7	2.33	down	5.03e-03	1
EYA1	265.31	620.51	2.33	down	1.82e-02	1
GPX3	1495.11	3494.13	2.33	down	2.83e-02	1
SORCS2	179.58	421.82	2.33	down	2.90e-02	1
SLC26A4	109.31	260.83	2.38	down	9.35e-03	1
MYO5B	231.2	551.79	2.38	down	1.67e-02	1
FGFBP2	163.29	397.78	2.44	down	2.82e-02	1
PCSK6	125.11	313.13	2.5	down	5.37e-03	1
DGKI	198.69	492.13	2.5	down	1.69e-02	1
TKTL1	127.73	322.78	2.5	down	4.30e-02	1
FRZB	182.00	463.27	2.56	down	1.68e-02	1
SDK2	128.78	328.1	2.56	down	1.81e-02	1
BCAN	2972.58	7976.38	2.7	down	2.75e-02	1
XIST	257.44	712.28	2.78	down	8.21e-04	1
BMP7	236.89	654.16	2.78	down	2.05e-02	1
SYNPO2	216.67	594.82	2.78	down	2.70e-02	1
SFRP4	261.6	729.68	2.78	down	4.34e-02	1
HOXB13	106.13	335.41	3.12	down	1.72e-04	1
PI15	510.27	1713.52	3.33	down	1.65e-03	1
SCUBE3	167.89	572.22	3.45	down	4.11e-02	1
FMOD	730.64	2705.39	3.7	down	4.09e-03	1
CHRDL1	124.73	647.37	5.26	down	4.36e-03	1
THBS4	187.42	997.69	5.26	down	1.41e-02	1
F5	133.82	704.83	5.26	down	2.96e-02	1
RELN	110.00	620.7	5.56	down	3.83e-02	1
PROM1	42.36	243.96	5.88	down	1.77e-02	1
ADAMTS14	314.00	158.76	1.98	up	4.78e-02	1
RN7SK	266.42	112.7	2.36	up	4.91e-02	1
RGS5	11231.76	2918.47	3.85	up	2.98e-02	1
			Group #2 (PTGDR, PTGFR, PTGER3)			
SFN	232.94	3647.73	16.67	down	4.79e-02	2
ROBO2	120.28	249.21	2.08	down	2.15e-02	2
ATP1B2	438.06	925.65	2.13	down	1.20e-02	2
LRRC61	125.21	270.09	2.17	down	1.41e-02	2

SDC1	1104.57	2440.85	2.22	down	1.49e-02	2
GLB1L2	162.74	374.09	2.27	down	9.10e-03	2
FGFBP2	171.96	401.22	2.33	down	1.38e-02	2
LINC00504	187.49	462.6	2.44	down	2.76e-02	2
TTYH1	216.75	568.64	2.63	down	7.99e-03	2
LYNX1	188.13	576.44	3.03	down	1.84e-05	2
FLG	334.04	1101.29	3.33	down	5.14e-03	2
RHCG	120.83	414.58	3.45	down	3.10e-02	2
PKP1	237.62	1770.05	7.69	down	8.43e-03	2
ILDR2	202.45	100.3	2.02	up	2.91e-02	2
CDH2	1731.6	844.24	2.05	up	3.66e-02	2
RNF128	513.87	248.71	2.07	up	6.90e-03	2
GBP1	5171.89	2471.26	2.09	up	7.98e-04	2
PDCD1LG2	248.79	106.21	2.34	up	9.06e-03	2
ALDH1A2	439.96	113.84	3.86	up	4.04e-02	2

Table S10. Cancer-specific expression and regulation patterns of prostanoid-metabolizing enzymes and receptors.

Genes	Gene expression tumor/normal	Gene copy number variations frequency, %	Differential expression tumor/normal		
			Promoter methylation status	miR expression	Master regulators expression
			Co-expressed genes in lung squamous cell carcinoma (LUSC)		
AKR1C3	↑	3.78 (del)	Hypermethylation ↓	miR-511 ↓; miR-184 ↔	FOXA2 ↓; LMNB1 ↑; MAX ↔; POLR2A ↔; SPI1 ↓
CBR1	↑	< cut-off	Unmethylated	miR-335 ↔	
CBR3	↑	< cut-off	Hypomethylation ↓	n/d	
			Co-expressed genes in esophageal carcinoma (ESCA)		
CBR1	↓	< cut-off	Unmethylated	miR-335 ↑	KDM4C ↔
CBR3	↓	< cut-off	Hypomethylation ↓	n/d	
			Signature of eight up-regulated genes in pancreatic adenocarcinoma		
TBXAS1	↑	< cut-off	Unmethylated	n/d	POLR2A ↔; CTCF ↑; IRF1 ↑; KLF4 ↑; STAG1 ↔
PTGIS	↑	6.42 (ampl)	Hypomethylation	n/d	
PTGDS	↑	13.76 (ampl)	Hypermethylation	n/d	
PTGES	↑	5.5 (ampl)	Unmethylated	n/d	
PTGES2	↑	9.17 (ampl)	Hypomethylation	n/d	
PTGES3	↑	4.59 (ampl)	Unmethylated	miR-223 ↓; miR-19a ↓; miR-605 ↓	
PRXL2B	↑	8.26 (ampl)<br>8.26 (del)	n/d	miR-486 ↓; miR-211 ↓; miR-423 ↓	
AKR1C3	↑	< cut-off	Hypermethylation	n/d	
CBR1	↑	< cut-off	Unmethylated	n/d	
CBR3	↑	< cut-off	Hypermethylation	n/d	
			Down-regulation of *PTGIS* and *PTGIR* genes in seven tumors		
			KICH		
PTGIS	↓	< cut-off	n/d	n/d	RNF2 ↔; SPI1 ↔; ZNF263 ↔
PTGIR	↓	< cut-off	n/d	miR-149 ↓; miR-326 ↓	
			KIRP		
PTGIS	↓	< cut-off	Hypomethylation	miR-496 ↓; miR-34a ↑	AR ↑; RNF2 ↔; SPI1 ↑; ZNF263 ↔
PTGIR	↓	< cut-off	Hypermethylation ↑	miR-589 ↑	
			LUAD		
PTGIS	↓	< cut-off	Hypomethylation	miR-34a ↑	SPI1 ↓; RELA ↔; RCOR1 ↔; MAZ ↔; CEBPB ↓; FOXA2 ↔ LMNB1 ↑
PTGIR	↓	< cut-off	Hypermethylation ↑	miR-326 ↓	
			LUSC		
PTGIS	↓	< cut-off	Hypomethylation	miR-34c ↓	SPI1 ↓; RELA ↔; RCOR1 ↔; MAZ ↑;

					CEBPB ↓ ; FOXA2 ↓ ; LMNB1 ↑
PTGIR	↓	< cut-off	Hypermethylation ↑	miR-326 ↓ ; miR-149 ↑	
			THCA		
PTGIS	↓	< cut-off	Hypomethylation	miR-34a ↑	n/d
PTGIR	↓	< cut-off	Hypermethylation ↑	n/d	n/d
			UCEC		
PTGIS	↓	< cut-off	Hypomethylation	miR-449a ↑ ; miR-449b ↑ ; miR-34a ↑	ZBTB7A ↓
PTGIR	↓	< cut-off	Hypermethylation ↑	miR-326 ↑ ; miR-589 ↑ ; miR-149 ↑	

Notes: ↑ or ↓ - statistical significant increase or decrease of gene expression or methylation status, respectively; ↔ no statistical significant changes (tumor/normal). Gene expression cut-off level |log2FoldChange| tumor/normal = 1, p-value < 0.01. Comparative analysis of gene expression was performed using TCGA datasets.

Cancer-specific copy number variations frequency (%) values of aimed genes were retrieved from cBioPortal database at cut-off values = 3%. Gene alterations are amplification (ampl) and deletion (del). Copy number variations in pancreatic cancer were evaluated with UTSW Nat Commun 2015 cBioPortal dataset. n/d – not determined.

Table S11. Gene expression changes of modifying proteins retrieved from the Biogrid database and physically interacting with the proteins involved in prostanoid signaling.

Target proteins	Post-translational modifications			
	**Ubiquitination / deubiquitination**	**Phosphorylation / dephosphorylation**	Glycosylation	Neddylation
	Gene expression in lung squamous cell carcinoma (LUSC)			
AKR1C3	UBE2W ↔, SIAH2 ↑	CDK9 ↔ , CSK ↔	n/d	n/d
CBR1	MARCH2 ↔, MARCH3 ↔, MARCH4 ↔, OTUB1 ↔, VHL ↔, WWP2 ↔	MAPK13↔ , PINK1 ↔ PRKAB1 ↔	B3GNT2 ↔ , OGT ↓	UBE2M ↔
CBR3	ARIH2 ↔	NEK4 ↔, PINK1 ↔ , RIOK1 ↔	n/d	n/d
	Gene expression in esophageal carcinoma (ESCA)			
CBR1	MARCH3 ↑ , MARCH2 ↔ , OTUB1 ↔ ,VHL ↔, WWP2 ↔	MAPK13 ↔ PINK1 ↔ , PRKAB1 ↔	B3GNT2 ↔ , OGT ↔	UBE2M ↔
CBR3	ARIH2 ↔	NEK4 ↔, PINK1 ↔ , RIOK1 ↔	n/d	n/d
	Signature of eight up-regulated genes in pancreatic adenocarcinoma			
TBXAS1	n/d	n/d	n/d	n/d
PTGIS	n/d	n/d	n/d	n/d
PTGDS	n/d	n/d	n/d	n/d
PTGES	n/d	n/d	n/d	n/d
PTGES2	n/d	STK24 ↑	n/d	n/d
PTGES3	n/d	MAP4K1 ↑ , MAP4K4 ↑ , PRKCD ↑ , PRKAA2 ↔	n/d	UBE2M ↑
PRXL2B	n/d	PDPK1 ↔	n/d	n/d
AKR1C3	UBE2W ↑ , SIAH2 ↔	CDK9 ↔, CSK ↑	n/d	n/d
CBR1	MARCH2 ↑ , MARCH3 ↑ , MARCH4 ↔, OTUB1 ↑ , VHL ↑ , WWP2 ↔	MAPK13 ↔, PINK1 ↑ , PRKAB1 ↑	B3GNT2 ↑ , OGT ↓	UBE2M ↑
CBR3	ARIH2 ↔	NEK4 ↔, PINK1 ↑ , RIOK1 ↔	n/d	n/d
	Down-regulation of *PTGIS* and *PTGIR* genes in seven tumors			
	KICH			
PTGIS	n/d	n/d	n/d	n/d
PTGIR	LNX1 ↔	MAP2K7 ↔, PRKCA↔, STK39 ↑	n/d	n/d
	KIRP			
PTGIS	n/d	n/d	n/d	n/d
PTGIR	LNX1 ↓	MAP2K7 ↔, PRKCA ↔, STK39 ↔	n/d	n/d
	LUAD			
PTGIS	n/d	n/d	n/d	n/d
PTGIR	LNX1↔	MAP2K7↔, PRKCA↔, STK39 ↑	n/d	n/d
	LUSC			
PTGIS	n/d	n/d	n/d	n/d
PTGIR	LNX1↔	MAP2K7↔, PRKCA↔, STK39 ↔	n/d	n/d
	THCA			
PTGIS	n/d	n/d	n/d	n/d
PTGIR	LNX1↔	MAP2K7↔, PRKCA↔, STK39 ↔	n/d	n/d
	UCEC			
PTGIS	n/d	n/d	n/d	n/d
PTGIR	LNX1↔	MAP2K7 ↓ , PRKCA ↓ , STK39 ↔	n/d	n/d

Notes: ↑ or ↓ - statistical significant increase or decrease of gene expression, respectively; ↔ no statistical significant changes (tumor/normal); n/d – not determined.

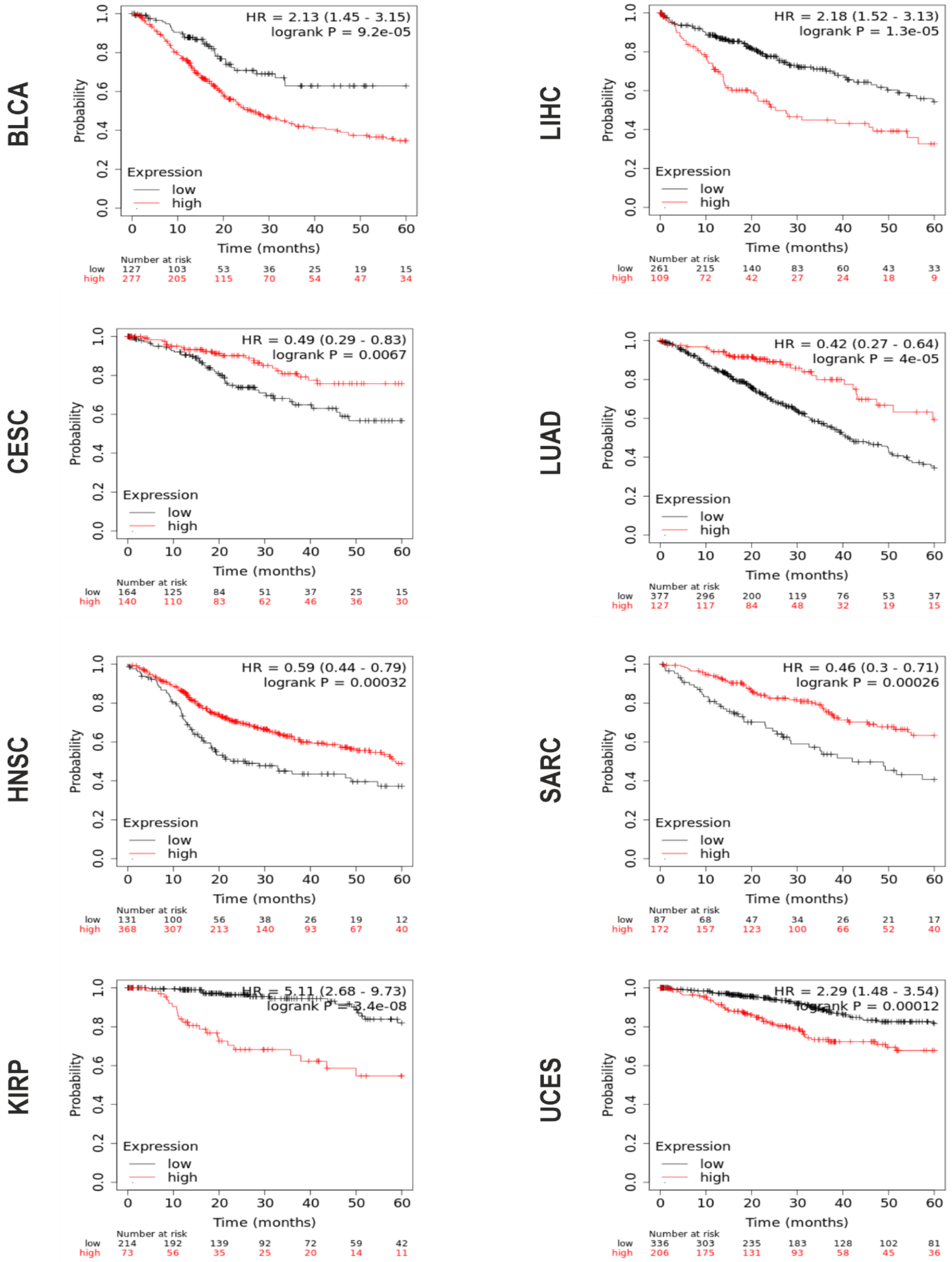

Figure S1. Kaplan-Meier plots: overall survival vs expression levels of target genes in several tumors.

Tumors abbreviations and gene expression signatures are presented below:

BLCA - Bladder Urothelial Carcinoma (*PTGIS, PTGDS, PTGFR* and *PTGER3*);

CESC - Cervical squamous cell carcinoma and endocervical adenocarcinoma (*PTGDS, AKR1C3, CBR1, CBR3, PTGDR, PTGDR2, PTGER2* and *PTGER4*);

HNSC - Head and Neck squamous cell carcinoma (*PTGDS, CBR3, PTGIR, PTGFR, PTGDR2* and *PTGER3*);

KIRP - Kidney renal papillary cell carcinoma (PTGIS, PTGDS, PTGES3, AKR1C3, CBR3, TBXA2R, PTGDR, PTGER1, PTGFR and PTGER3);

LIHC - Liver hepatocellular carcinoma (*PTGES2, PTGES3, PRXL2B, AKR1C3* and *PTGES*); LUAD - Lung adenocarcinoma (*PTGDS* and *PTGDR2*);

SARC - Sarcoma (TBXAS1, PTGDS, AKR1C3, PTGIS, CBR3, TBXA2R, PTGDR, PTGFR, PTGER3 and PTGER4);

UCES - Uterine Corpus Endometrial Carcinoma (*PTGER1, PTGER3* and *PTGER4*).

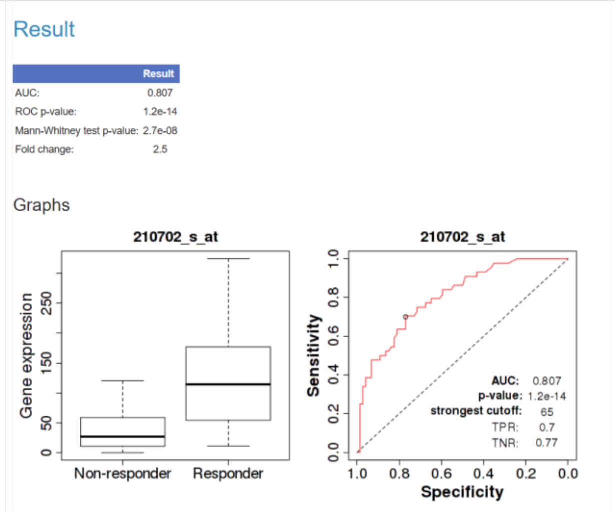

Settings: PTGIS (210702_s_at); pathological complete response sample; fluorouracil, epirubicin and cyclophosphamide treatment; histology: HER2+; AUC = 0.81; fold change = 2.5. 74 non-responders and 44 responders

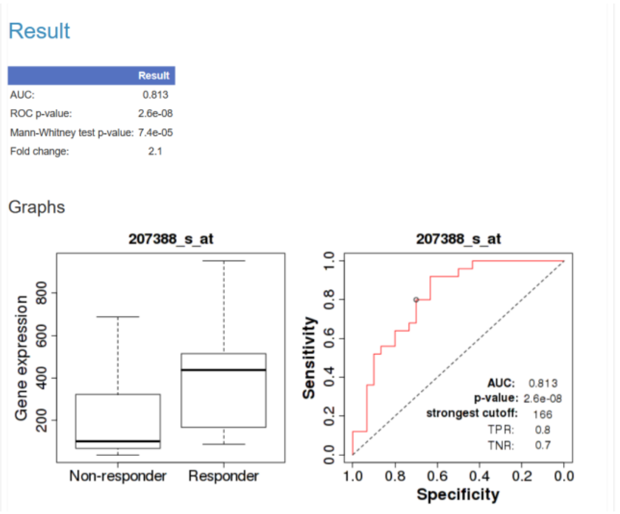

Settings: PTGES (207399_s_at); pathological complete response sample; fluorouracil, epirubicin and cyclophosphamide treatment; histology: HER2+ and ER-; AUC = 0.81; fold change = 2.1. 30 non-responders and 25 responders.

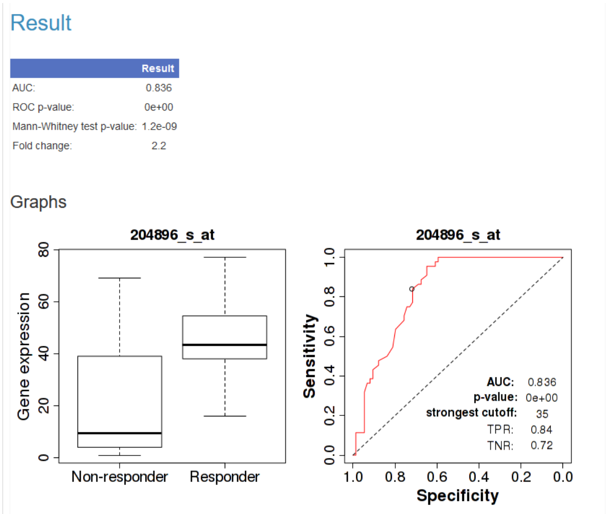

Settings: PTGER4 (204896_s_at); pathological complete response sample; fluorouracil, epirubicin and cyclophosphamide treatment; histology: HER2+;

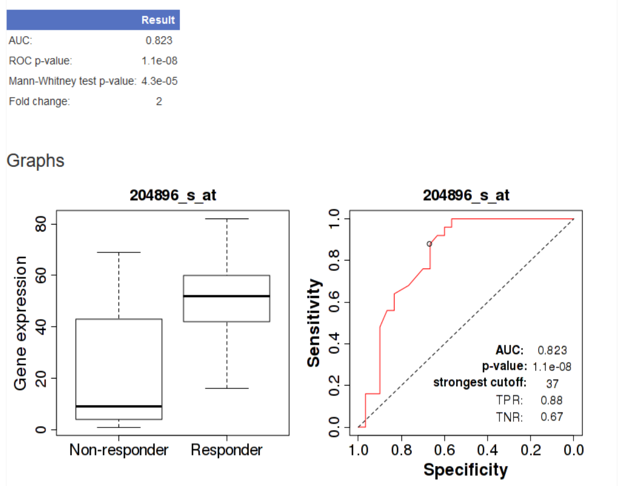

Settings: PTGER4 (204896_s_at); pathological complete response sample; fluorouracil, epirubicin and cyclophosphamide treatment; histology: HER2+;

AUC = 0.84; fold change = 2.2. 74 non-responders and 44 responders	AUC = 0.82; fold change = 2.0. 30 non-responders and 25 responders.

Figure S2. A. Predictive value of gene expression of prostanoid-metabolizing enzymes and prostanoid receptor in breast cancer.

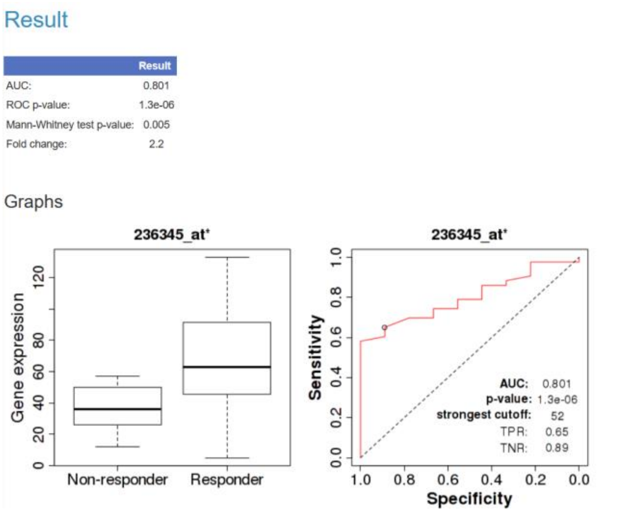 Settings: TBXAS1 (236345_at), pathological response sample; taxane treatment; histology: serous; grade: G3; debulk optimal; AUC=0.80; fold change = 2.2. 268 responders, 35 non-responders.	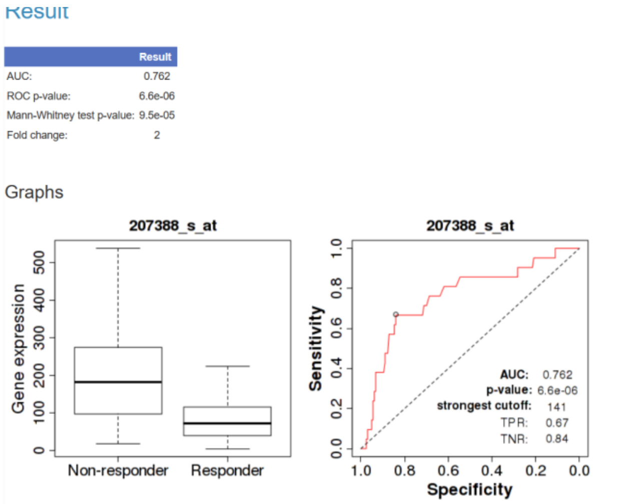 Settings: PTGES (207388_s_at), RFS (6 months) sample; taxane treatment; histology: serous; grade: G3; debulk suboptimal; AUC=0.76; fold change = 2.0. 163 responders, 21 non-responders.
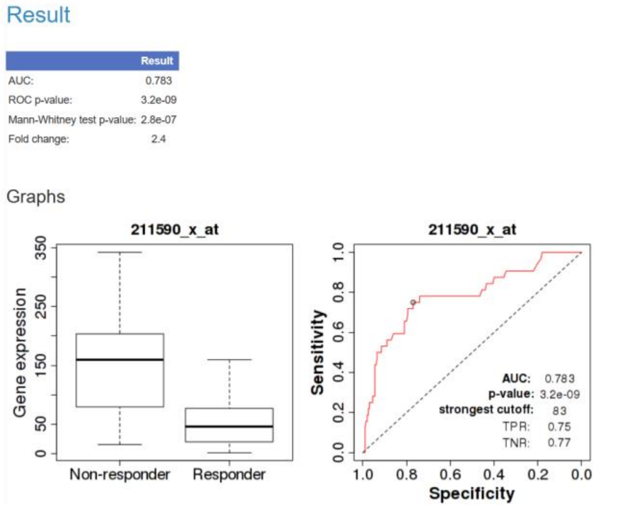 Settings: TBXA2R (211590_x_at), RFS (6 months) sample; platin treatment; histology: serous; grade: G3; debulk suboptimal; AUC=0.78; fold change = 2.4. 200 responders, 32 non-responders.	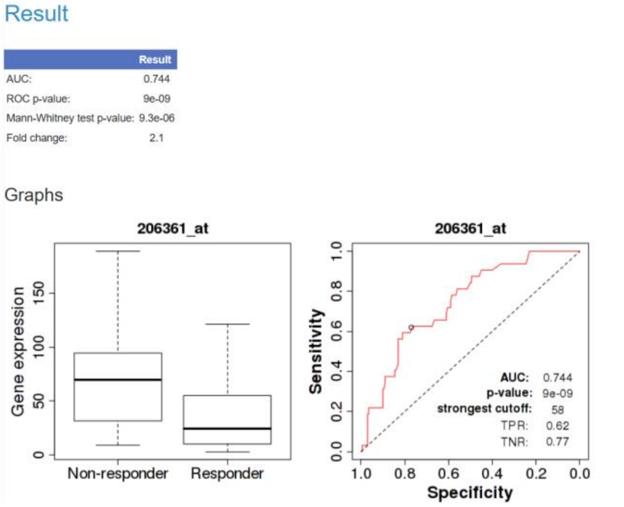 Settings: PTGDR2 (206361_at), RFS (6 months) sample; platin treatment; histology: serous; grade: G3; debulk suboptimal; AUC=0.74; fold change = 2.1. 200 responders, 32 non-responders.

Figure S2. B. Predictive value of gene expression of prostanoid-metabolizing enzymes and prostanoid receptor in ovarian cancer.

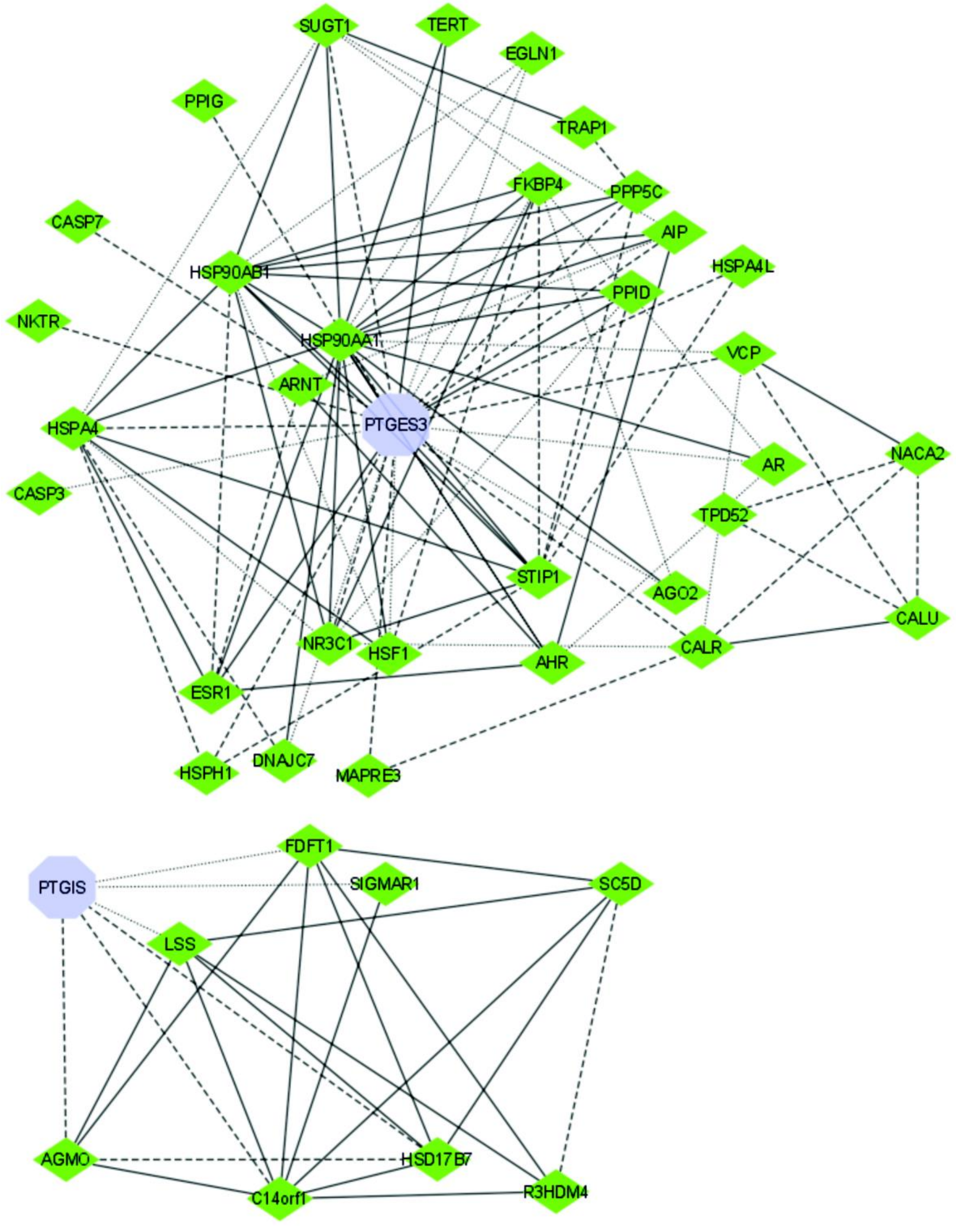

Figure S3. Protein-protein interaction subnetworks with PTGIS and PTGES3 and their physically interacting protein partners, sharing similar subcellular localization pattern with target proteins. Edges, selected with dotted, dashed and solid lines, indicate 0.4-0.5, 0.5-0.7 and 0.7-0.99 STRINGdb combined scores (experimentally_determined_interactions), respectively.

References

1. Deng, Q.-P.; Wang, M.-J.; Zeng, X.; Chen, G.G.; Huang, R.-Y. Effects of Glycyrrhizin in a Mouse Model of Lung Adenocarcinoma. *Cell Physiol Biochem* **2017**, *41*, 1383–1392, doi:10.1159/000467897.
2. Liu, Y.; Yang, S.; Li, M.-Y.; Huang, R.; Ng, C.S.H.; Wan, I.Y.P.; Long, X.; Wu, J.; Wu, B.; Du, J.; et al. Tumorigenesis of Smoking Carcinogen 4-(Methylnitrosamino)-1-(3-Pyridyl)-1-Butanone Is Related to Its Ability to Stimulate Thromboxane Synthase and Enhance Stemness of Non-Small Cell Lung Cancer Stem Cells. *Cancer Lett* **2016**, *370*, 198–206, doi:10.1016/j.canlet.2015.10.017.

3. Cathcart, M.C.; Gately, K.; Cummins, R.; Drakeford, C.; Kay, E.W.; O'Byrne, K.J.; Pidgeon, G.P. Thromboxane Synthase Expression and Correlation with VEGF and Angiogenesis in Non-Small Cell Lung Cancer. *Biochim Biophys Acta* **2014**, *1842*, 747–755, doi:10.1016/j.bbadis.2014.01.011.
4. Li, H.; Lee, M.-H.; Liu, K.; Wang, T.; Song, M.; Han, Y.; Yao, K.; Xie, H.; Zhu, F.; Grossmann, M.; et al. Inhibiting Breast Cancer by Targeting the Thromboxane A2 Pathway. *NPJ Precis Oncol* **2017**, *1*, 8, doi:10.1038/s41698-017-0011-4.
5. Sasaki, Y.; Ochiai, T.; Takamura, M.; Kondo, Y.; Yokoyama, C.; Hara, S. Role of Prostacyclin Synthase in Carcinogenesis. *Prostaglandins Other Lipid Mediat.* **2017**, *133*, 49–52, doi:10.1016/j.prostaglandins.2017.05.001.
6. Li, H.Y.; McSharry, M.; Walker, D.; Johnson, A.; Kwak, J.; Bullock, B.; Neuwelt, A.; Poczobutt, J.M.; Sippel, T.R.; Keith, R.L.; et al. Targeted Overexpression of Prostacyclin Synthase Inhibits Lung Tumor Progression by Recruiting CD4+ T Lymphocytes in Tumors That Express MHC Class II. *Oncoimmunology* **2018**, *7*, e1423182, doi:10.1080/2162402X.2017.1423182.
7. Kwon, O.K.; Ha, Y.-S.; Na, A.-Y.; Chun, S.Y.; Kwon, T.G.; Lee, J.N.; Lee, S. Identification of Novel Prognosis and Prediction Markers in Advanced Prostate Cancer Tissues Based on Quantitative Proteomics. *Cancer Genomics Proteomics* **2020**, *17*, 195–208, doi:10.21873/cgp.20180.
8. Lichao, S.; Liang, P.; Chunguang, G.; Fang, L.; Zhihua, Y.; Yuliang, R. Overexpression of PTGIS Could Predict Liver Metastasis and Is Correlated with Poor Prognosis in Colon Cancer Patients. *Pathol Oncol Res* **2012**, *18*, 563–569, doi:10.1007/s12253-011-9478-4.
9. Jin, R.; Strand, D.W.; Forbes, C.M.; Case, T.; Cates, J.M.M.; Liu, Q.; Ramirez-Solano, M.; Milne, G.L.; Sanchez, S.; Wang, Z.Y.; et al. The Prostaglandin Pathway Is Activated in Patients Who Fail Medical Therapy for Benign Prostatic Hyperplasia with Lower Urinary Tract Symptoms. *Prostate* **2021**, *81*, 944–955, doi:10.1002/pros.24190.
10. Zou, R.; Zheng, M.; Tan, M.; Xu, H.; Luan, N.; Zhu, L. Decreased PTGDS Expression Predicting Poor Survival of Endometrial Cancer by Integrating Weighted Gene Co-Expression Network Analysis and Immunohistochemical Validation. *Cancer Manag Res* **2020**, *12*, 5057–5075, doi:10.2147/CMAR.S255753.
11. He, L.P.; Chen, Y.F.; Yang, J. [Investigation on the role and mechanism of prostagland in D2 synthase in non-small cell lung cancer]. *Zhonghua Yi Xue Za Zhi* **2017**, *97*, 3022–3027, doi:10.3760/cma.j.issn.0376-2491.2017.38.016.
12. Pan, J.; Zhang, L.; Huang, J. Prostaglandin D2 Synthase/Prostaglandin D2/TWIST2 Signaling Inhibits Breast Cancer Proliferation. *Anticancer Drugs* **2021**, *32*, 1029–1037, doi:10.1097/CAD.0000000000001111.
13. Hu, S.; Ren, S.; Cai, Y.; Liu, J.; Han, Y.; Zhao, Y.; Yang, J.; Zhou, X.; Wang, X. Glycoprotein PTGDS Promotes Tumorigenesis of Diffuse Large B-Cell Lymphoma by MYH9-Mediated Regulation of Wnt-β-Catenin-STAT3 Signaling. *Cell Death Differ* **2022**, *29*, 642–656, doi:10.1038/s41418-021-00880-2.
14. Jiang, P.; Cao, Y.; Gao, F.; Sun, W.; Liu, J.; Ma, Z.; Xie, M.; Fu, S. SNX10 and PTGDS Are Associated with the Progression and Prognosis of Cervical Squamous Cell Carcinoma. *BMC Cancer* **2021**, *21*, 694, doi:10.1186/s12885-021-08212-w.
15. Bie, Q.; Li, X.; Liu, S.; Yang, X.; Qian, Z.; Zhao, R.; Zhang, X.; Zhang, B. YAP Promotes Self-Renewal of Gastric Cancer Cells by Inhibiting Expression of L-PTGDS and PTGDR2. *Int J Clin Oncol* **2020**, *25*, 2055–2065, doi:10.1007/s10147-020-01771-1.
16. Liu, C.; Yang, J.C.; Armstrong, C.M.; Lou, W.; Liu, L.; Qiu, X.; Zou, B.; Lombard, A.P.; D'Abronzo, L.S.; Evans, C.P.; et al. AKR1C3 Promotes AR-V7 Protein Stabilization and Confers Resistance to AR-Targeted Therapies in Advanced Prostate Cancer. *Mol Cancer Ther* **2019**, *18*, 1875–1886, doi:10.1158/1535-7163.MCT-18-1322.
17. Penning, T.M. AKR1C3 (Type 5 17β-Hydroxysteroid Dehydrogenase/Prostaglandin F Synthase): Roles in Malignancy and Endocrine Disorders. *Mol Cell Endocrinol* **2019**, *489*, 82–91, doi:10.1016/j.mce.2018.07.002.
18. Russo, A.; Biselli-Chicote, P.M.; Kawasaki-Oyama, R.S.; Castanhole-Nunes, M.M.U.; Maniglia, J.V.; de Santi Neto, D.; Pavarino, É.C.; Goloni-Bertollo, E.M. Differential Expression of Prostaglandin I2 Synthase Associated with Arachidonic Acid Pathway in the Oral Squamous Cell Carcinoma. *J Oncol* **2018**, *2018*, 6301980, doi:10.1155/2018/6301980.
19. Wang, T.; Jing, B.; Sun, B.; Liao, Y.; Song, H.; Xu, D.; Guo, W.; Li, K.; Hu, M.; Liu, S.; et al. Stabilization of PTGES by Deubiquitinase USP9X Promotes Metastatic Features of Lung Cancer via PGE2 Signaling. *Am J Cancer Res* **2019**, *9*, 1145–1160.
20. Nagaraja, A.S.; Dorniak, P.L.; Sadaoui, N.C.; Kang, Y.; Lin, T.; Armaiz-Pena, G.; Wu, S.Y.; Rupaimoole, R.; Allen, J.K.; Gharpure, K.M.; et al. Sustained Adrenergic Signaling Leads to Increased Metastasis in Ovarian Cancer via Increased PGE2 Synthesis. *Oncogene* **2016**, *35*, 2390–2397, doi:10.1038/onc.2015.302.
21. Wang, T.; Jing, B.; Xu, D.; Liao, Y.; Song, H.; Sun, B.; Guo, W.; Xu, J.; Li, K.; Hu, M.; et al. PTGES/PGE2 Signaling Links Immunosuppression and Lung Metastasis in Gprc5a-Knockout Mouse Model. *Oncogene* **2020**, *39*, 3179–3194, doi:10.1038/s41388-020-1207-6.

22. Stamatakis, K.; Jimenez-Martinez, M.; Jimenez-Segovia, A.; Chico-Calero, I.; Conde, E.; Galán-Martínez, J.; Ruiz, J.; Pascual, A.; Barrocal, B.; López-Pérez, R.; et al. Prostaglandins Induce Early Growth Response 1 Transcription Factor Mediated Microsomal Prostaglandin E2 Synthase Up-Regulation for Colorectal Cancer Progression. *Oncotarget* **2015**, *6*, 39941–39959, doi:10.18632/oncotarget.5402.
23. Kim, S.-H.; Roszik, J.; Cho, S.-N.; Ogata, D.; Milton, D.R.; Peng, W.; Menter, D.G.; Ekmekcioglu, S.; Grimm, E.A. The COX2 Effector Microsomal PGE2 Synthase 1 Is a Regulator of Immunosuppression in Cutaneous Melanoma. *Clin Cancer Res* **2019**, *25*, 1650–1663, doi:10.1158/1078-0432.CCR-18-1163.
24. Kim, S.; Lee, E.S.; Lee, E.J.; Jung, J.Y.; Lee, S.B.; Lee, H.J.; Kim, J.; Kim, H.J.; Lee, J.W.; Son, B.H.; et al. Targeted Eicosanoids Profiling Reveals a Prostaglandin Reprogramming in Breast Cancer by MicroRNA-155. *J Exp Clin Cancer Res* **2021**, *40*, 43, doi:10.1186/s13046-021-01839-4.
25. Garo, L.P.; Ajay, A.K.; Fujiwara, M.; Gabriely, G.; Raheja, R.; Kuhn, C.; Kenyon, B.; Skillin, N.; Kadowaki-Saga, R.; Saxena, S.; et al. MicroRNA-146a Limits Tumorigenic Inflammation in Colorectal Cancer. *Nat Commun* **2021**, *12*, 2419, doi:10.1038/s41467-021-22641-y.
26. Ke, J.; Shen, Z.; Li, M.; Peng, C.; Xu, P.; Wang, M.; Zhu, Y.; Zhang, X.; Wu, D. Prostaglandin E2 Triggers Cytochrome P450 17α Hydroxylase Overexpression via Signal Transducer and Activator of Transcription 3 Phosphorylation and Promotes Invasion in Endometrial Cancer. *Oncol Lett* **2018**, *16*, 4577–4585, doi:10.3892/ol.2018.9165.
27. Kong, J.; Shen, S.; Zhang, Z.; Wang, W. Identification of Hub Genes and Pathways in Cholangiocarcinoma by Coexpression Analysis. *Cancer Biomark* **2020**, *27*, 505–517, doi:10.3233/CBM-190038.
28. Gu, Y.; Chen, G.; Du, Y. Screening of Prognosis-Related Genes in Primary Breast Carcinoma Using Genomic Expression Data. *J Comput Biol* **2020**, *27*, 1030–1040, doi:10.1089/cmb.2019.0131.
29. Zhou, L.; Yang, C.; Zhong, W.; Wang, Q.; Zhang, D.; Zhang, J.; Xie, S.; Xu, M. Chrysin Induces Autophagy-Dependent Ferroptosis to Increase Chemosensitivity to Gemcitabine by Targeting CBR1 in Pancreatic Cancer Cells. *Biochem Pharmacol* **2021**, *193*, 114813, doi:10.1016/j.bcp.2021.114813.
30. Osawa, Y.; Yokoyama, Y.; Shigeto, T.; Futagami, M.; Mizunuma, H. Decreased Expression of Carbonyl Reductase 1 Promotes Ovarian Cancer Growth and Proliferation. *Int J Oncol* **2015**, *46*, 1252–1258, doi:10.3892/ijo.2014.2810.
31. Yun, M.; Choi, A.J.; Woo, S.R.; Noh, J.K.; Sung, J.-Y.; Lee, J.-W.; Eun, Y.-G. Inhibition of Carbonyl Reductase 1 Enhances Metastasis of Head and Neck Squamous Cell Carcinoma through β-Catenin-Mediated Epithelial-Mesenchymal Transition. *J Cancer* **2020**, *11*, 533–541, doi:10.7150/jca.34303.
32. Yamanouchi, R.; Harada, K.; Ferdous, T.; Ueyama, Y. Low Carbonyl Reductase 1 Expression Is Associated with Poor Prognosis in Patients with Oral Squamous Cell Carcinoma. *Mol Clin Oncol* **2018**, *8*, 400–406, doi:10.3892/mco.2018.1548.
33. Zhang, M.; Wang, Y.; Jiang, L.; Song, X.; Zheng, A.; Gao, H.; Wei, M.; Zhao, L. LncRNA CBR3-AS1 Regulates of Breast Cancer Drug Sensitivity as a Competing Endogenous RNA through the JNK1/MEK4-Mediated MAPK Signal Pathway. *J Exp Clin Cancer Res* **2021**, *40*, 41, doi:10.1186/s13046-021-01844-7.
34. Liu, S.; Zhan, N.; Gao, C.; Xu, P.; Wang, H.; Wang, S.; Piao, S.; Jing, S. Long Noncoding RNA CBR3-AS1 Mediates Tumorigenesis and Radiosensitivity of Non-Small Cell Lung Cancer through Redox and DNA Repair by CBR3-AS1 /MiR-409-3p/SOD1 Axis. *Cancer Lett* **2022**, *526*, 1–11, doi:10.1016/j.canlet.2021.11.009.
35. Zhou, Q.; Ding, W.; Weng, Y.; Ding, G.; Xia, G.; Xu, J.; Xu, K.; Ding, Q. NOS3 895G>T and CBR3 730G>A Are Associated with Recurrence Risk in Non-Muscle-Invasive Bladder Cancer with Intravesical Instillations of THP. *Chemotherapy* **2018**, *63*, 191–197, doi:10.1159/000489402.
36. Misawa, K.; Mima, M.; Satoshi, Y.; Imai, A.; Mochizuki, D.; Ishikawa, R.; Kita, J.; Yamaguchi, Y.; Endo, S.; Misawa, Y.; et al. Prostanoid Receptor Genes Confer Poor Prognosis in Head and Neck Squamous Cell Carcinoma via Epigenetic Inactivation. *J Transl Med* **2020**, *18*, 31, doi:10.1186/s12967-020-02214-1.
37. Misawa, K.; Imai, A.; Kanazawa, T.; Mima, M.; Yamada, S.; Mochizuki, D.; Yamada, T.; Shinmura, D.; Ishikawa, R.; Kita, J.; et al. G Protein-Coupled Receptor Genes, PTGDR1, PTGDR2, and PTGIR, Are Candidate Epigenetic Biomarkers and Predictors for Treated Patients with HPV-Associated Oropharyngeal Cancer. *Microorganisms* **2020**, *8*, E1504, doi:10.3390/microorganisms8101504.
38. O'Sullivan, A.G.; Mulvaney, E.P.; Kinsella, B.T. Regulation of Protein Kinase C-Related Kinase (PRK) Signalling by the TPα and TPβ Isoforms of the Human Thromboxane A2 Receptor: Implications for Thromboxane- and Androgen- Dependent Neoplastic and Epigenetic Responses in Prostate Cancer. *Biochim Biophys Acta Mol Basis Dis* **2017**, *1863*, 838–856, doi:10.1016/j.bbadis.2017.01.011.
39. Werfel, T.A.; Hicks, D.J.; Rahman, B.; Bendeman, W.E.; Duvernay, M.T.; Maeng, J.G.; Hamm, H.; Lavieri, R.R.; Joly, M.M.; Pulley, J.M.; et al. Repurposing of a Thromboxane Receptor Inhibitor Based on a Novel Role in Metastasis Identified by Phenome-Wide Association Study. *Mol Cancer Ther* **2020**, *19*, 2454–2464, doi:10.1158/1535-7163.MCT-19-1106.

40. Orr, K.; Buckley, N.E.; Haddock, P.; James, C.; Parent, J.-L.; McQuaid, S.; Mullan, P.B. Thromboxane A2 Receptor (TBXA2R) Is a Potent Survival Factor for Triple Negative Breast Cancers (TNBCs). *Oncotarget* **2016**, *7*, 55458–55472, doi:10.18632/oncotarget.10969.
41. Mulvaney, E.P.; Shilling, C.; Eivers, S.B.; Perry, A.S.; Bjartell, A.; Kay, E.W.; Watson, R.W.; Kinsella, B.T. Expression of the TPα and TPβ Isoforms of the Thromboxane Prostanoid Receptor (TP) in Prostate Cancer: Clinical Significance and Diagnostic Potential. *Oncotarget* **2016**, *7*, 73171–73187, doi:10.18632/oncotarget.12256.
42. Dash, P.; Ghatak, S.; Topi, G.; Satapathy, S.R.; Ek, F.; Hellman, K.; Olsson, R.; Mehdawi, L.M.; Sjölander, A. High PGD2 Receptor 2 Levels Are Associated with Poor Prognosis in Colorectal Cancer Patients and Induce VEGF Expression in Colon Cancer Cells and Migration in a Zebrafish Xenograft Model. *Br J Cancer* **2022**, *126*, 586–597, doi:10.1038/s41416-021-01595-4.
43. Lv, J.; Li, L. Hub Genes and Key Pathway Identification in Colorectal Cancer Based on Bioinformatic Analysis. *Biomed Res Int* **2019**, *2019*, 1545680, doi:10.1155/2019/1545680.
44. Fu, C.; Mao, W.; Gao, R.; Deng, Y.; Gao, L.; Wu, J.; Zhang, S.; Shen, Y.; Liu, K.; Li, Q.; et al. Prostaglandin F2α-PTGFR Signaling Promotes Proliferation of Endometrial Epithelial Cells of Cattle through Cell Cycle Regulation. *Anim Reprod Sci* **2020**, *213*, 106276, doi:10.1016/j.anireprosci.2020.106276.
45. Alkhateeb, A.; Rezaeian, I.; Singireddy, S.; Cavallo-Medved, D.; Porter, L.A.; Rueda, L. Transcriptomics Signature from Next-Generation Sequencing Data Reveals New Transcriptomic Biomarkers Related to Prostate Cancer. *Cancer Inform* **2019**, *18*, 1176935119835522, doi:10.1177/1176935119835522.
46. Akiyama, K.; Ohga, N.; Maishi, N.; Hida, Y.; Kitayama, K.; Kawamoto, T.; Osawa, T.; Suzuki, Y.; Shinohara, N.; Nonomura, K.; et al. The F-Prostaglandin Receptor Is a Novel Marker for Tumor Endothelial Cells in Renal Cell Carcinoma. *Pathol Int* **2013**, *63*, 37–44, doi:10.1111/pin.12031.
47. Anderson, K.S.; Cramer, D.W.; Sibani, S.; Wallstrom, G.; Wong, J.; Park, J.; Qiu, J.; Vitonis, A.; LaBaer, J. Autoantibody Signature for the Serologic Detection of Ovarian Cancer. *J Proteome Res* **2015**, *14*, 578–586, doi:10.1021/pr500908n.
48. Jiménez-Segovia, A.; Mota, A.; Rojo-Sebastián, A.; Barrocal, B.; Rynne-Vidal, A.; García-Bermejo, M.-L.; Gómez-Bris, R.; Hawinkels, L.J.A.C.; Sandoval, P.; Garcia-Escudero, R.; et al. Prostaglandin F2α-Induced Prostate Transmembrane Protein, Androgen Induced 1 Mediates Ovarian Cancer Progression Increasing Epithelial Plasticity. *Neoplasia* **2019**, *21*, 1073–1084, doi:10.1016/j.neo.2019.10.001.
49. Masato, M.; Miyata, Y.; Kurata, H.; Ito, H.; Mitsunari, K.; Asai, A.; Nakamura, Y.; Araki, K.; Mukae, Y.; Matsuda, T.; et al. Oral Administration of E-Type Prostanoid (EP) 1 Receptor Antagonist Suppresses Carcinogenesis and Development of Prostate Cancer via Upregulation of Apoptosis in an Animal Model. *Sci Rep* **2021**, *11*, 20279, doi:10.1038/s41598-021-99694-y.
50. Asting, A.G.; Iresjö, B.-M.; Nilsberth, C.; Smedh, U.; Lundholm, K. Host Knockout of E-Prostanoid 2 Receptors Reduces Tumor Growth and Causes Major Alterations of Gene Expression in Prostaglandin E2-Producing Tumors. *Oncol Lett* **2017**, *13*, 476–482, doi:10.3892/ol.2016.5448.
51. Heidegger, H.; Dietlmeier, S.; Ye, Y.; Kuhn, C.; Vattai, A.; Aberl, C.; Jeschke, U.; Mahner, S.; Kost, B. The Prostaglandin EP3 Receptor Is an Independent Negative Prognostic Factor for Cervical Cancer Patients. *Int J Mol Sci* **2017**, *18*, E1571, doi:10.3390/ijms18071571.
52. Czogalla, B.; Kuhn, C.; Heublein, S.; Schmöckel, E.; Mayr, D.; Kolben, T.; Trillsch, F.; Burges, A.; Mahner, S.; Jeschke, U.; et al. EP3 Receptor Is a Prognostic Factor in TA-MUC1-Negative Ovarian Cancer. *J Cancer Res Clin Oncol* **2019**, *145*, 2519–2527, doi:10.1007/s00432-019-03017-8.
53. De Paz Linares, G.A.; Opperman, R.M.; Majumder, M.; Lala, P.K. Prostaglandin E2 Receptor 4 (EP4) as a Therapeutic Target to Impede Breast Cancer-Associated Angiogenesis and Lymphangiogenesis. *Cancers (Basel)* **2021**, *13*, 942, doi:10.3390/cancers13050942.
54. Schotten, L.M.; Darwiche, K.; Seweryn, M.; Yildiz, V.; Kneuertz, P.J.; Eberhardt, W.E.E.; Eisenmann, S.; Welter, S.; Sisson, B.E.; Pietrzak, M.; et al. DNA Methylation of PTGER4 in Peripheral Blood Plasma Helps to Distinguish between Lung Cancer, Benign Pulmonary Nodules and Chronic Obstructive Pulmonary Disease Patients. *Eur J Cancer* **2021**, *147*, 142–150, doi:10.1016/j.ejca.2021.01.032.
55. Zhang, Y.; Huang, J.; Zou, Q.; Che, J.; Yang, K.; Fan, Q.; Qian, D.; Wu, J.; Bao, E.; Song, L.; et al. Methylated PTGER4 Is Better than CA125, CEA, Cyfra211 and NSE as a Therapeutic Response Assessment Marker in Stage IV Lung Cancer. *Oncol Lett* **2020**, *19*, 3229–3238, doi:10.3892/ol.2020.11434.
56. Hiken, J.F.; McDonald, J.I.; Decker, K.F.; Sanchez, C.; Hoog, J.; VanderKraats, N.D.; Jung, K.L.; Akinhanmi, M.; Rois, L.E.; Ellis, M.J.; et al. Epigenetic Activation of the Prostaglandin Receptor EP4 Promotes Resistance to Endocrine Therapy for Breast Cancer. *Oncogene* **2017**, *36*, 2319–2327, doi:10.1038/onc.2016.397.

57. Sinha, N.; Gaston, D.; Manders, D.; Goudie, M.; Matsuoka, M.; Xie, T.; Huang, W.-Y. Characterization of Genome-Wide Copy Number Aberrations in Colonic Mixed Adenoneuroendocrine Carcinoma and Neuroendocrine Carcinoma Reveals Recurrent Amplification of PTGER4 and MYC Genes. *Hum Pathol* **2018**, *73*, 16–25, doi:10.1016/j.humpath.2017.08.036.
